# Development of Self-Assembling *bis*-1,4-Dihydropyridines: Detailed Studies of Bromination of Four Methyl Groups and Bromine Nucleophilic Substitution

**DOI:** 10.3390/molecules29010161

**Published:** 2023-12-27

**Authors:** Martins Kaukulis, Martins Rucins, Davis Lacis, Aiva Plotniece, Arkadij Sobolev

**Affiliations:** 1 Latvian Institute of Organic Synthesis, Aizkraukles Str. 21, LV-1006 Riga, Latvia; rucins@osi.lv (M.R.); davis.lacis@osi.lv (D.L.); aiva@osi.lv (A.P.); arkady@osi.lv (A.S.); 2 Faculty of Materials Science and Applied Chemistry, Riga Technical University, P. Valdena Str. 3, LV-1048 Riga, Latvia; 3 Department of Pharmaceutical Chemistry, Faculty of Pharmacy, Riga Stradiņš University, Konsula 21, LV-1007 Riga, Latvia

**Keywords:** *bis*-1,4-dihydropyridine, bromination, pyridinium bromide–perbromide, pyridinium amphiphile, self-assembling properties, DLS

## Abstract

One of the most important steps in the synthesis of 1,4-dihydropyridine (1,4-DHP) amphiphiles is the bromination of methyl groups in positions 2 and 6 of the entire ring. However, up to now, only N-bromosuccinimide was mainly used for bromination 1,4-DHPs. In this work, the synthesis of *bis*-1,4-DHP derivatives with ethyl and dodecyl ester groups attached to 1,4-DHP ring at positions 3 and 5 was performed by Hantzsch synthesis. The experimental studies were carried out to find out the best conditions and the agent for the tetra bromination of *bis*-1,4-DHP methyl groups at positions 2 and 6. Four different brominating agents were screened. The use of pyridinium bromide–perbromide in ethyl acetate was found to be optimal for the bromination of methyl groups. The bromination reaction was followed by the synthesis of cationic pyridine moiety containing amphiphilic *bis*-1,4-DHP derivatives. By nucleophilic substitution of bromine with various substituted pyridines, 12 new amphiphilic *bis*-1,4-DHP derivatives were obtained. Evaluation of self-assembling properties of tetracationic *bis*-1,4-dihydropyridine derivatives by dynamic light scattering (DLS) measurements was also performed.

## 1. Introduction

Due to their unique properties, 1,4-Dihydropyridines (1,4-DHP) are an important family of pharmacologically active substances [1,2,3]. Many drugs to treat hypertension are based on a 1,4-DHP scaffold [4,5]. In addition to calcium channel blocking activity, 1,4-DHPs have been reported to possess a wide range of biological activities [6,7,8].

Two decades ago, it was revealed that single- and double-charged amphiphiles on the base of a 1,4-DHP scaffold were able to introduce plasmid DNA into various cell lines in vitro. The most optimal transfection efficiency has been achieved by 1,4-DHPs containing dodecyl alkyl ester chains at positions 3 and 5 and polar pyridinium groups at positions 2 and 6 of the 1,4-DHP ring [9]. The latter studies revealed that modification of amphiphilic 1,4-DHP structures may lead to significant changes in delivery activity and in the properties of nanoparticles formed by these compounds. Thus, modification of the cationic head-group demonstrated the influence of the electronic nature of the substituents on the ability of compounds to bind and transfer pDNA into the cells [9]. Introducing a methylene group spacer between the cationic moiety and the linker in 1,4-DHP molecules also influenced delivery activity and demonstrated the cell type specificity of the tested compounds [10,11]. While the synthesis of 1,4-dihydropyridines has advanced greatly since its discovery by Arthur Hantzsch in 1881, the synthesis and study of bis-1,4-dihydropyridine (*bis*-1,4-DHP) derivatives and compounds with a linking group still remains unexplored [12].

Generally, linkage of *bis*-1,4-DHPs is constructed in Hantzsch synthesis using various dialdehydes, thus, the use of 1,4- or 1,3-benzenedicarboxaldehydes [13,14,15], 4,4′-biphenyldicarbaldehyde [15], terphenyldicarboxaldehyde [16] and more complex bis(aldehydes) containing ether linkages [14,17,18,19] was reported. Also, tris, tetrakis and hexakis 1,4-DHP derivatives were synthesized via Hantzsch synthesis starting from the corresponding polyaldehydes [20]. Another approach to construct bis-, tris- and tetrakis-1,4-DHP derivatives starts with the synthesis of 4-(4-hydroxyphenyl)-1,4-DHP derivatives, followed by their reaction with dibromoxylene or pyridine or dibromomethyl, trisbromomethyl and tetrakisbromomethyl-substituted aromatic cycles [21].

Several studies have revealed the biological importance of *bis*-1,4-DHPs [11,22,23].

Previously, our research group synthesized and studied tetracationic *bis*-1,4-DHP derivative with one phenyl ring as a linker [24]. This compound can be considered as a gemini cationic lipid. Gemini cationic lipids and surfactants have been intensively studied for more than two decades [25,26,27]. These compounds have a great potential due to their superior surface characteristics, such as lower critical micelle concentration, higher surface activity and beneficial viscoelastic qualities when compared to their monomeric counterparts [28,29].

Bromination of the C–H bond is the first choice to transform it into a flexible synthetic tool, allowing broad reactions through cross-coupling and substitution [30,31,32]. 

**Figure 1:**
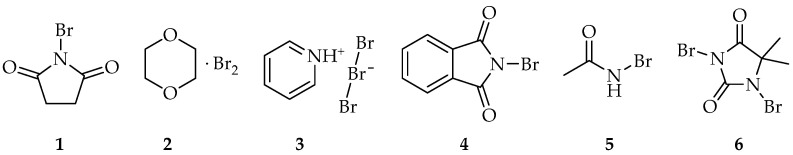
Commonly used brominating agents for bromination of 2- and 6-methyl groups of 1,4-DHPs.

One of the key steps in the preparation of cationic 1,4-DHPs is the bromination of methyl groups at positions 2 and 6 of its ring [33]. The most commonly used reagent is N-bromosuccinimide (NBS) (**1**, Figure 1). For 1,4-DHP-3,5-dicarboxylates with long alkyl chains, bromination requires long reaction times (20–24 h) with low to moderate yields of the products (up to 75%) [34]. Also, bromination of methyl groups of tetradodecyl 4,4′-(1,4-phenylene)*bis*(2,6-dimethyl-1,4-dihydropyridine-3,5-dicarboxylate) was performed by NBS in methanol at r.t. for 12 h, giving 1,4-phenylene-bis(2,6-bis(bromomethyl)-1,4-dihydropyridine with a yield of 43% [24]. One of the first reported selective methods for bromination of methyl groups at the 2 and 6 positions of the 1,4-DHP ring was the use of bromine in chloroform or dioxane dibromide (**2**, Figure 1) in dioxane [35]. Pyridinium bromide–perbromide (PBPB) (**3**, Figure 1) was also used to produce mono- or dibromomethyl 1,4-DHPs. The reaction was reported by two research groups, but no new papers using this reagent have been published since then [36,37]. N-bromophthalimide (**4**, Figure 1), N-bromoacetamide (**5**, Figure 1) and 1,3-dibromo-5,5-dimethylhydantoin (DBDMH) (**6**, Figure 1) were also utilized as bromine sources for chiral phosphoric acid catalyzed enantioselective desymmetrization of 1,4-DHPs via selective bromination of methyl groups [32].

As a part of our studies towards the development of novel self-assembling non-viral delivery systems and establishing the recommendations of structure–activity relationships, we continued the study and development of the original cationic amphiphilic *bis*-1,4-DHPs connected using [1,1′-biphenyl]-4,4′-dicarbaldehyde in Hantzsch synthesis to create a conjunction between two 1,4-DHP rings. The main part of this work was dedicated to elaboration of the most suitable method for the bromination of four methyl groups of *bis*-1,4-DHP aiming to find the best brominating agent and the optimal conditions. The last step of synthesis of cationic 1,4-DHPs was bromine nucleophilic substitution using various pyridine derivatives. Evaluation of self-assembling properties of tetracationic *bis*-1,4-dihydropyridine derivatives was also performed.

## 2. Results and Discussion

### 2.1. Synthesis of bis-1,4-Dihydropyrdines

**Scheme 1:**
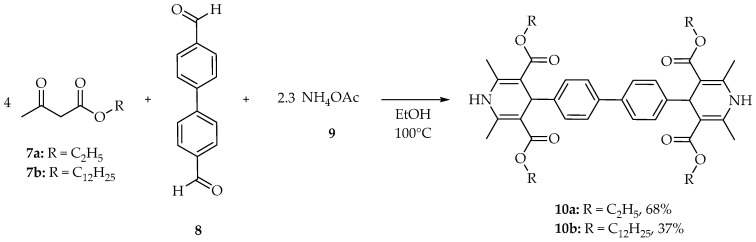
Synthesis of *bis*-1,4-dihydropyridines **10a**,**b** via Hantzsch synthesis.

The starting *bis*-1,4-dihydropyridines were synthesized via a classical Hantzsch synthesis (Scheme 1) from the corresponding 3-oxobutanoate ester **7a**,**b**, [1,1′-biphenyl]-4,4′-dicarbaldehyde (**8**) and ammonium acetate (**9**). Dodecyl ester **7b** was chosen based on the previously reported data that only 1,4-DHP containing long alkyl ester chains may form particles [9], and ethyl ester **7a** was chosen to evaluate the influence of the length of ester moieties on the self-assembly properties. Depending on the length of the ester, the reaction mixture was refluxed in a pressure vessel using ethanol as a solvent at 100 °C for two or three days to give *bis*-1,4-dihydropiridines **10a**,**b** in 68% and 37% yields, respectively. The yield of compound **10a** is comparable to that previously reported in the literature (72%), however, for the synthesis reported in the literature, a 25 wt % ammonia–water solution was used as the nitrogen source instead of ammonium acetate, and the reaction mixture was refluxed at 80 °C for 96 h [16]. The structure of compound **10a** was confirmed by the ^1^H NMR spectra, which is in agreement with what has been previously reported in the literature [16]. The structure of the compound **10b** was established and confirmed based on ^1^H, ^13^C NMR (Figures S2 and S3, Supplementary Materials) spectral and elemental analysis data.

### 2.2. Bromination of Methyl Groups at Positions 2 and 6 of 1,4-Dihydropyridine

**Scheme 2:**
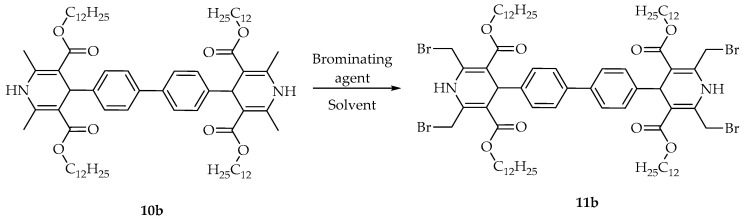
The desired tetrabrominated product **11b** of bis-1,4-dihydropyridine **10b**.

As mentioned above, since bromination of the methyl groups is one of the most important steps in the synthesis of 1,4-dihydropyridine-based amphiphiles, particular attention was dedicated to the optimization of this reaction conditions and search for the best brominating agent. In view of the fact that amphiphilic properties are more characteristic for C_12_ ester containing 1,4-dihydropyridines, *bis*-1,4-dihydropyridine **10b** was used as a model compound in our current studies (Scheme 2). 

Bromination experiments were started by screening of the most commonly used brominating agents for 1,4-dihydropyrdines—NBS (**1**), and also a structurally similar brominating agent—1,3-dibromo-5,5-dimethylhydantoin (**6**, DBDMH). The use of DBDMH (**6**) would reduce the necessary amount of brominating reagent required since this molecule contains two bromine atoms compared to NBS (**1**), which only has one bromine atom. Additionally other brominating agents—pyridinium bromide–perbromide (**3**) and structurally related trimethylphenylammonium bromide–perbromide (PTAB) (**12**)—were tested. Results are presented in Table 1.

Briefly, all the experiments were performed on a 50 mg (0.04 mmol) scale of compound **10b**. In all experiments, the brominating agent was dissolved in 25 mL of solvent and added portion-wise to the reaction mixture and stirred at r.t. for the time indicated in Table 1. The total amount of the solvent used is given in Table 1. The reaction dynamics were monitored by TLC and after the disappearance of the signal of the starting 1,4-DHP **10b**, the reaction was quenched and the products were isolated and purified by column chromatography. 

Bromination of 1,4-dihydropyridines with NBS (**1**) is usually carried out in methanol [9]. Starting *bis*-1,4-dihydropyridines **10a**,**b** have rather low solubility in MeOH, therefore, a large volume of the solvent is necessary (Table 1, entries 1 and 6). The reaction took 48 h to complete (for both reagents) and the reaction yields were quite low—41% (Table 1, entry 1) using NBS (**1**) and 22% (Table 1, entry 6) using DBDMH (**6**). The formation of tetrabromide **11b** was confirmed by ^1^H and ^13^C NMR spectra. In ^1^H, the NMR spectra characteristic proton signal of the methyl groups as a singlet at 2.34 ppm was not observed, but a new signal as an AB-system at 4.63 and 4.93 ppm was registered (Figure S6, Supplementary Materials). The analysis of the experiments led to the conclusion that it was necessary to increase the yield of the reaction and also to decrease the required amount of a solvent and the reaction time. 

Bromination of methyl groups of 1,4-DHPs containing phenyl or methyl substituents at position 4 and a fluorine-rich dodecyl ester chain in chloroform with a 69% yield has been reported in the literature [38]. After stirring in CHCl_3_ for two hours, the reaction of compound **10b** with DBDMH (**6**) (Table 1, entry 7), did not lead to the formation of the desired product **11b**. Based on the literature data [39], it can be proposed that partial or full lactonization could happen, resulting in the formation of furopyridines with various degrees of lactonization. We were not able to separate and confirm the structures of individual side-products. In the reaction with NBS (**1**) afforded the desired product along with several side products that were either partially brominated or over brominated, according to ^1^H-NMR spectra data. It was demonstrated, that use of chloroform as a solvent in the case of bromination with NBS not only reduced the amount of the required solvent, but also reduced the reaction time to 3 h and increased the isolated yield by 13% from 41% to 54% (Table 1, entries 1 and 2).

In the cases where the solubility of starting compounds in methanol is low, reactions in a mixture of methanol and chloroform are also reported [40]. Since the use of DBDMH (**6**, Table 1, entry 7) for bromination of comp. **10b** in chloroform resulted only in the formation of a mixture of furopyridines, further experiments with this reagent in a mixture of methanol and chloroform were not performed. The use of NBS (**1**) in a mixture of chloroform and methanol in volumetric ratio of 1:3 (Table 1, entry 3) significantly reduced the amount of the solvent required compared to the reaction in MeOH from 175 mL to 35 mL and also reduced the reaction time for a half, although the isolated yield of 48% was slightly higher than that obtained in MeOH (Table 1, entry 3 versus entry 1), and the yield was lower than that for the reaction carried out in chloroform (Table 1, entry 3 versus entry 2).

An exchange of CHCl_3_ to ethyl acetate in the solvent mixture EtOAc/MeOH (1:3) for the reaction with NBS (**1**) (Table 1, entry 4) gave a comparable isolated yield to the reaction carried out in chloroform, but the reaction time was 20 h. This could be explained by the presence of methanol and its role in the reaction mechanism. It has been proposed that after NBS addition to the double bond of 1,4-DHP, followed by succinimide elimination, methoxide addition to the second position of 1,4-DHP takes place. Such an intermediate has been identified by NMR [41]. The reaction with DBDMH for 20 h in the same solvent system—EtOAc/MeOH (1:3) (**6**, Table 1, entry 8)—gave product **11b,** with a yield of 48%.

Use of NBS in ethyl acetate for bromination of **10b** allowed us to increase the reaction yield to 73% (Table 1, entry 5) which is the highest yield among the tested conditions (Table 1, entries 1 to 5).

Based on these results, we can conclude that the use of a less polar solvent afforded higher isolated yields of product **11b,** together with shorter reaction times. However, the reaction is highly dependent on the brominating agent, which can either lead to the formation of the desired product or promote the side reactions—formation of partially brominated products or a mixture of furopyridines.

By varying the amount of NBS from 4.1 to 5.0 equivalents, using a mixture of EtOAC/MeOH in volumetric ratio of 1:3 as a solvent, no significant changes in the course of the reaction were observed. Since the use of other brominating agents in the bromination of 1,4-dihydropyridine is not widely reported in the literature, other brominating agents such as pyridinium bromide–perbromide (**3**) and trimethylphenylammonium bromide–perbromide (**12**) were tested. Initially, the reactions were carried out in a mixture of ethyl acetate and methanol at ratio 1:3. After stirring for 30 min, reaction with 4.1 equivalents of pyridinium bromide–perbromide (**3**) afforded the desired reaction product **11b** in a 50% yield (Table 1, entry 9).

To understand the effect of time on the reaction rate, the reaction was carried out for 2 and 4 h (Table 1, entries 10 and 11). If the reaction time exceeds 4 h, the yield of fully brominated product decreases significantly. Increasing the amount of this brominating agent **3** also resulted in a significant decrease in the yield of the product (Table 1, entry 12). In the previous experiments with NBS (Table 1, entries 1,3 and 4), it was observed that the presence of MeOH in the reaction mixture could affect the reaction rate and the yield of the product. The reaction with brominating agent **3** was performed in ethyl acetate as a solvent (Table 1, entry 13), which resulted in a significant increase of isolated yield to 85% in a short reaction time of only 30 min.

Another bromide–perbromide reagent **12** was tested under conditions which were previously found to be optimal. This reagent gave quite a similarly isolated yield (59%, Table 1, entry 14) compared to NBS (**1**, 56%, Table 1, entry 4). However, the yield of the reaction with PTAB **12** was significantly lower than that for the PBPB **3** (Table 1, entries 13 and 14).

**Scheme 3:**
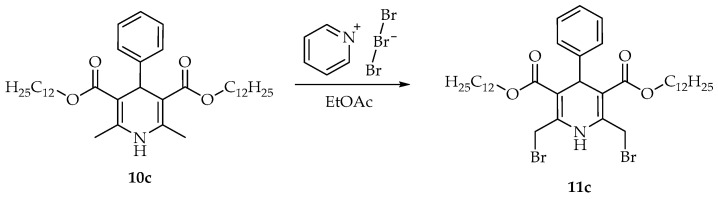
Bromination of didodecyl 2,6-dimethyl-4-phenyl-1,4-dihydropyridine-3,5-dicarboxylate (**10c**).

Application of the best conditions found for bromination of *bis*-1,4-DHP **10b** to the bromination of didodecyl 2,6-dimethyl-4-phenyl-1,4-dihydropyridine-3,5-dicarboxylate (**10c**) was performed to prove generality of the method (Scheme 3). Compound **10c** is a key intermediate in the synthesis of synthetic cationic lipids based on 1,4-dihydropyridine core. Overall, 1,4-DHP **10c** was obtained using three-component Hantzsch synthesis starting from dodecyl acetoacetate, benzaldehyde and ammonium acetate in ethanol under reflux according to the reported method [9]. Bromination was carried out on a 50 mg (0.08 mmol) scale using ethyl acetate as a solvent, 2.1 eq. of pyridinium bromide–perbromide as brominating agent and the reaction time was 30 min. The yield of bromination product—2,6-*bis*(bromomethyl)-1,4-dihydropyridine **11c**—was higher than the previously reported one based on use of NBS (**1**) as the brominating agent [34] (87% vs. 75%), together with a shorter reaction time (0.5 h vs. 24 h).

In order to test the conditions found on a larger scale, two experiments were carried out on a 0.5 g (0.41 mmol) scale. For bromination with NBS (**1**), it was found that the best results are obtained when it is added dropwise [42]. Therefore, the addition method was also tested in these two experiments. 

Dropwise addition of an EtOAc solution of pyridinium bromide–perbromide (**3**) (100 mL) required 40 min to complete, followed by stirring for an additional 30 min. The isolated yield of **11c** was 77%, which is not a significant decrease compared to the 50 mg scale experiments (87% yield). When the brominating reagent was added in portions, after 30 min of stirring, the isolated yield of **11c** was only 61%. Since the addition time was significantly shorter than in the first case, it might be worth increasing the reaction time to 1 h if the reagent is added in portions. Even if dropwise addition is chosen, the reaction is significantly shorter than bromination with NBS (**1**) reported in the literature [9,34,42].

We selected dropwise addition of the EtOAc solution of pyridinium bromide–perbromide (**3**) as optimal for bromination of *bis*-1,4-dihydropyrdines **10**.

Using these conditions for the bromination of tetraethyl *bis*-1,4-dihydropyridine **10a**, the isolated yield was significantly lower—only 38% when compared to bromination of tetradodecyl *bis*-1,4-dihydropyridine **10b**. To determine whether this is due to the reaction conditions or the substrate, additional experiments using NBS (**1**) were performed. The isolated yield of tetra(bromomethyl)-1,4-DHP **11a** in the reaction with NBS (**1**) reached only 23%. The main difference between compounds **10a** and **10b** is the length of the ester chains. During work-up, it was observed that in the case of compound **10a**, the number of by-products was increased. It can be concluded that the sterical hindrance of long alkyl ester chains could play a significant role in the bromination of *bis*-1,4-dihydropyridines with pyridinium bromide–perbromide (**3**).

### 2.3. Synthesis of bis-1,4-Dihydropyridine Amphiphiles

**Scheme 4:**
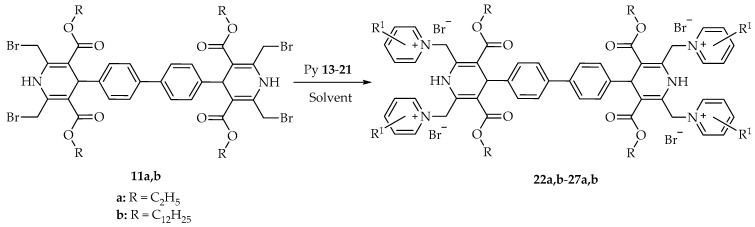
The synthesis of *bis*-1,4-dihydropyridine amphiphiles **22a**,**b**–**27a**,**b**.

Previously, our research group has demonstrated that various cationic 3,5-bis(dodecyloxycarbonyl)-1,4-DHP derivatives possessed self-assembling properties and showed significance for the development of gene delivery agents [9,34]. As a result of this study, a series of bis-1,4-dihydropyridine amphiphiles were synthesized to complement the compound group for estimation of structure–activity relationships and evaluation of the influence of fragment duplication in the molecule. Previously mentioned bis-1,4-dihydropyridine amphiphiles were prepared from the obtained tetra(bromomethyl)-1,4-dihydropyridine **11a**,**b** using different pyridines in bromine nucleophilic substitution reactions (Scheme 4). Nine different pyridines were used, including an unsubstituted pyridine **13**, an electron-donating group (EDG) containing pyridines **14**–**16**, 3-bromopyridine **17**, bulky substituted pyridines **18**–**20** and an electron-withdrawing group (EWG) containing pyridine **21**. Pyridine derivatives, condition of reactions and product yields are mentioned in Table 2.

Initially, the experiments were performed as follows: to a solution of bromide **11b** (0.06 mmol) in acetone (4 mL), 4.5 equivalents of pyridine were added (Table 2, entry 2) according to the synthetic procedure proposed for the preparation of monomeric cationic 1,4-DHP amphiphiles [9]. After stirring for 2 h, a precipitate was observed in the reaction mixture. According to the ^1^H-NMR spectrum, in the precipitate derivatives of *bis*-1,4-DHP with partially substituted bromine in the 2,6-bromomethyl groups were identified. This precipitate was dissolved in methanol, and 4.5 equivalents of pyridine were added additionally. After 1 day of stirring in the reaction mixture, only partially substituted bromine *bis*-1,4-DHP derivatives were observed.

Several experimental and theoretical studies of quaternization reactions of substituted pyridines have revealed that the solvent and the electronic nature of the substituents of pyridine have a strong influence on the reaction rate [43,44,45].

In order to improve the solubility of partially substituted intermediates and reach complete nucleophilic substitution, an experiment with an excess of pyridine, which was also used as a solvent, was performed. *Bis*(2,6-*bis*(bromomethyl)-1,4-dihydropyridine **11b** (0.06 mol) was dissolved in pyridine (20 mL), and after stirring for 30 min, some precipitate formed, but only a few drops of methanol allowed us to obtain a homogenous reaction mixture. The reaction mixture was stirred for 2 days after then concentrated under reduced pressure. The residual crude product was suspended in acetone and the resulting precipitate was filtered off and washed with cold diethyl ether to give a pure desired product **22b** in a 54% yield (Table 2, entry 2). In the ^1^H-NMR spectra, the AB-system was shifted to the downfield (from 4.63 and 4.93 ppm to 5.65 and 6.16 ppm), which confirmed the presence of quaternized nitrogen of pyridinium substituents (Figure S11, Supplementary Materials) The presence of the AB-system in the ^1^H-NMR spectra also confirmed the diastereotopic properties of -CH_2_- protons for amphiphilic *bis*-1,4-DHP **22b**. The diastereotopic properties of -CH_2_- protons were also demonstrated for monomeric 1,4-DHP amphiphiles in the literature [9].

Using this procedure to obtain cationic compound **22a** from bromide **11a** in its pure form failed in our hands (Table 2, entry 1). The search for a suitable chromatographic method to purify the product ended up with no result. A specific eluent system for normal-phase chromatography of compounds with cationic moiety was found in the literature. This eluent system consisted of a saturated aqueous solution of KNO_3_, water and acetone [46]. A solution consisting of saturated KNO_3_, water and acetone in the volumetric ratio of 1:4:25 allowed us to obtain a good separation of product **22a** from the reaction mixture. Product **22a** was obtained in a 69% yield.

Based on this data, other reactions with pyridine derivatives **15**–**17** and **18**,**20** were carried out according to the same principle by dissolving bromides **11a**,**b** in the corresponding pyridines. To achieve complete dissolution of tetra(bromomethyl)-1,4-DHP **11a** in the reaction mixture, DMF (up to 10 mL) was added additionally as a solvent (Table 2, entries 3,5,7,11,13,15).

In the reaction of **11a** with 4-*n*-propylpyridine (**15**), product **24a** was obtained in a good yield of 80%, however, its dodecyl ester substituted analogue **24b** was obtained in a 16% yield only, which might be due to the sterical hindrance of long alkyl ester chain (Table 2, entries 5 and 6). In the case of 4-(pentan-3-yl)pyridine (**16**), product **25b** was obtained in a higher yield than ethyl ester **25a** (Table 2, entries 7 and 8). Tetra(bromomethyl)-1,4-DHP **11a** readily reacted with pyridine **17,** giving product **26a** in an 80% yield after stirring for 2 days, but tetra(bromomethyl)-1,4-DHP **11b** afforded a lower yield (62%) of the product **26b** even after stirring for 4 days (Table 2, entries 9 and 10). Experiments with pyridine **21** (Table 2, entries 17 and 18) containing strong EWG were unsuccessful. With prolonged stirring time and even at elevated temperatures, formation of the desired product was not observed. 

Reactions with sterically hindered pyridines **18**–**20** yielded the desired tetrasubstituted product only in the case of 4-(3-phenylpropyl)pyridine (**18**), giving cationic derivatives **27a**,**b** with 71% and 57% yields, respectively (Table 2, entries 11 and 12). Experiments with pyridines **18** and **20** were carried out as previously mentioned, but reaction with pyridine **19**, which is a solid, was performed by dissolving tetra(bromomethyl)-1,4-DHPs **11a**,**b** in a small amount of DMF, followed by addition of 4.1 eq. of pyridine **19**. The reaction mixture was stirred for 4–12 days and monitored by thin-layer chromatography (TLC).

*N,N*-Dimethylpyridin-4-amine (Table 2, entries 3 and 4) was chosen as the electron-donating group containing pyridine. Tetra(bromomethyl)-1,4-DHP **11a** had very low solubility in acetone, EtOAc, MeOH and MeCN, therefore, DMF was chosen as the solvent. Product **23a** was purified similarly to compound **22a**. Tetra(bromomethyl)-1,4-DHP **11b** had excellent solubility in acetone so the reaction was carried out in it. After 1 day of stirring, precipitation was observed. The reaction mixture was further stirred for an additional day and the resulting precipitate was filtered off and washed with cold diethyl ether to obtain pure product **23b** with a 60% yield (Table 2, entry 4).

The structures of the compounds **22a**,**b**–**27a**,**b** were established and confirmed by ^1^H, ^13^C NMR and HRMS data (Figures S8–S41, Supplementary Materials).

From the obtained results it can be concluded that reaction conditions for bromination of the methyl groups of *bis*-1,4-dihydropyridine are optimized. Another limiting step for synthesis of corresponding amphiphiles is bromine nucleophilic substitution. Tetra(bromomethyl)-1,4-DHPs have relatively low solubility. Generally, short alkyl ester chain-containing derivatives react more easily than long alkyl ester chain-containing ones. Comparing the obtained yields of pyridine and *N,N*-dimethylaminopyridine-derived amphiphiles to the yields of analogous monomeric 1,4-DHP derivatives in this reaction, it can be concluded that not only did the reaction time increase at least two times, but also the reaction yields decreased by approximately 20% [9]. The important influence of the nature of substituent at pyridine ring was also observed. Pyridines containing strong EWG and sterically hindered substituents did not react under the tested conditions.

### 2.4. Estimation of Self-Assembling Properties

A characteristic feature of synthetic lipid-like molecules, including amphiphilic 1,4-DHPs, is their self-assembling properties. Using the dynamic light scattering (DLS) approach, it was possible to measure the hydrodynamic average diameter (Z_av_), polydispersity index (PDI), zeta-potential (Z_pot_) and evaluate the stability of a nanoparticle generated by *bis*-1,4-DHPs **22**–**27** in an aqueous medium. By dissolving a lipid-like compound in an organic solvent and then dispersing the lipid solution into water under vigorous stirring, the ethanol injection method was used to produce nanoparticles. The final concentration of a sample solution was 0.5 mM. A hand-held, small-scale extruder with a 100 nm pore size was used to homogenize liposome dispersion in water. The self-assembling properties of eleven bis-1,4-DHP were tested. The DLS measurements were performed for both samples—extruded (Table 3) and non-extruded (Table S1, Supplementary Materials). The DLS measurements were performed on the freshly prepared samples, additionally, hydrodynamic average diameter (Z_av_) and polydispersity index (PDI) were measured after sample storage at room temperature for 3, 7 and 14 days.

According to obtained data, the average diameter of freshly prepared nanoparticles of *bis*-1,4-DHP amphiphiles in an aqueous medium ranged from 61 to 3111 nm for non-extruded samples (Table S1) and ranged from 73 to 1039 nm for extruded samples (Table 3), respectively. The structure of the amphiphilic moiety influenced the values of the nanoparticles’ average diameter. Extrusion for the sample formed by compound **22a** was not performed because at the beginning particles with an acceptable size were already obtained. In almost all cases, an average diameter of extruded samples was smaller than the non-extruded ones, except for comp. **E-25b** and **25b** (Table 3, entry 7 vs. Table S1, entry 7) and **E-26b** and **26b** (Table 3, entry 9 vs. Table S1, entry 9), where both diameter sizes were comparable. Mainly, the compounds with ethyl ester formed particles with a smaller average diameter, except compounds **26**, where the average diameters of the extruded particles of comp **E-26a** were larger for non-extruded samples **26a** (Table 3, entry 8 vs. Table S1, entry 8). The diameters of the nanoparticles obtained from *bis*-1,4-DHP amphiphiles **E-22b** and **E-23b** are 363 nm and 897 nm, respectively. These values are significantly larger than for the corresponding monomers, 113 nm and 100 nm, respectively [9]. The average diameter of the nanoparticles of *bis*-1,4-DHP amphiphiles after 3 days storage at r.t. ranged from 100 to 1098 nm for non-extruded samples (Table S1) and from 140 to 596 nm for extruded samples (Table 3), after 7 days of storage it ranged from 70 to 2102 nm for non-extruded samples (Table S1) and from 26 to 618 nm for extruded samples (Table 3) and after 14 days storage it ranged from 71 to 465 nm for non-extruded samples (Table S1) and from 37 to 585 nm for extruded samples (Table 3), respectively. Examples of the obtained DLS measurement graphs are presented in Supplementary Materials, Figures S42–S46. It was demonstrated in our previous works that the self-assembling properties and the ability to form nanoparticles by the amphiphilic 1,4-DHPs are highly dependent on the substituents of 1,4-DHP cycle [47]. Also, in this study, the influence of the substituents of the rings on the parameters of the nanoparticles is demonstrated. From the obtained data, it follows that amphiphilic *bis*-1,4-DHP derivatives substituted by dodecyl esters seem to be less stable than the ones with ethyl esters. Thus, nanoparticles formed by comp. **E-23b** after 7 days of storage formed samples with a PDI value of 1, which confirms a high level of sample heterogeneity; comp. **E-27b** after 15 days of storage undergoes aggregation, and the sample becomes cloudy; PDI values of the samples prepared from other *bis*-1,4-DHP amphiphiles comprising dodecylester moieties are higher than for the corresponding ethyl derivatives. It could be proposed that introduction of four dodecyl moieties in amphiphile molecule is excessive for formation of an optimal sample of nanoparticles, as these systems are not stable over the time. It was mentioned that according to the literature, the homogeneity of samples and size of vesicles also depend on the preparation method, for example, the main drawback of the ethanol injection method is connected with the final formation of a heterogeneous population of liposomes [48].

PDI represents the distribution of size populations in a tested sample. In the case of drug delivery system for the development using lipid-based carriers, a PDI of 0.3 and below is preferable as it indicates a homogenous population of vesicles [49]. Although, also depending on the lipid structure, PDI values below 0.4 were confirmed to have a narrow size distribution [50,51]. PDI values below 0.4 indicated a fair homogeneity for the freshly prepared samples of comp. **22a**, **E**-**22b**, **E-23a**, **E-27a** and **E-27b**. An increase in PDI values in time confirms a decrease in the system’s homogeneity due to possible formation of particles with different size and particle aggregation. The heavy aggregation of particles was observed for particles formed by comp. **E-27b**, when, after 2-week storage, large particles were observed in a solution. There are also similar conclusions regarding a sample of comp. **E-23b** when the PDI value reaches the value of 1 after storage for 7 days. PDI values of the samples formed by other compounds demonstrate high ±SD values, confirming the heterogeneity of the size of the particles. 

The surface charges of obtained nanoparticles were positive, with intervals between 9.7–31 mV. The surface potential determines the strength of intra-particle interactions and particle stability. It was underlined in the literature that particles with low zeta potential values can stick together and form aggregates. The general dividing line between stable and unstable suspensions is usually taken at +30 mV or −30 mV. Particles with zeta potentials more positive than +30 mV or more negative than −30 mV are usually considered stable [52,53,54]. We can suggest that particles formed by *bis*-1,4-DHP amphiphiles are less stable due to low zeta potential values.

## 3. Materials and Methods

All reagents were purchased from Acros Organics (Geel, Belgium), Sigma-Aldrich (St. Louis, MO, USA), BLDpharm (Karlsruhe, Germany) or Merck KGaA (Darmstadt, Germany) in >97% purity and used without further purification. TLC was performed on silica gel 60 F_254_ aluminium sheets 20 × 20 cm (Merck KGaA, Darmstadt, Germany). ^1^H-NMR spectra were recorded with a Bruker Fourier (300 MHz), a Bruker Avance Neo (400 MHz) or Bruker Avance Neo (600 MHz) spectrometer, but ^13^C-NMR spectra were recorded with a Bruker Avance Neo (101 MHz) or Bruker Avance Neo (151 MHz) spectrometer (Bruker Biospin Gmbh, Rheinstetten, Germany). The coupling constants *J* are expressed in Hertz (Hz). The chemical shifts of the hydrogen and carbon atoms are presented in parts per million (ppm) and referred to the residual signals of the non-deuterated CDCl_3_ (δ: 7.26) or partially deuterated DMSO-*d_6_* (δ: 2.50) solvent for ^1^H-NMR spectra and CDCl_3_ (δ: 77.2) or DMSO-*d_6_* (δ: 39.5) solvent for ^13^C-NMR, respectively. Multiplicities are abbreviated as s = singlet; bs = broad singlet; d = doublet; t = triplet; sext = sextet; m = multiplet. Low-resolution mass spectra (MS) were determined on an Acquity UPLC system (Waters, Milford, MA, USA) connected to a Waters SQ Detector-2 operating in the electrospray ionization (ESI) positive on mode on a Waters Acquity UPLC^®^ BEH C18 column (1.7 µm, 2.1 mm × 50 mm, using gradient elution with acetonitrile (0.01% formic acid) in water (0.01% formic acid). High resolution mass spectra (HRMS) were determined on an Acquity UPLC system (Waters, Milford, MA, USA) connected to a Waters Synapt GII Q-ToF operating in the ESI positive ion mode on a Waters Acquity UPLC^®^ BEH C18 column (1.7 µm, 2.1 mm × 50 mm, using gradient elution with acetonitrile (0.01% formic acid) in water (0.01% formic acid). Melting points (m.p.) were determined on an OptiMelt (Stanford Research Systems, Sunnyvale, CA, Canada) and are uncorrected. Elemental analyses were determined on an Elemental Combustion System ECS 4010 (Costech International S.p.A., Milano, Italy) at the Laboratory of Chromatography of Latvian Institute of Organic Synthesis. The DLS measurements of the aqueous solutions of nanoparticles were carried out on a Zetasizer Nano ZSP (Malvern Panalytical Ltd., Malvern, UK) instrument with Malvern Instruments Ltd. Software 8.01.4906.

### 3.1. Synthesis of bis-1,4-Dihydropyridines ***10a**,**b***

*Tetraethyl 4,4′-([1,1′-biphenyl]-4,4′-diyl)bis(2,6-dimethyl-1,4-dihydropyridine-3,5-dicarboxylate)* (**10a**): Ethyl 3-oxobutanoate (**7a**, 1.25 g, 9.6 mmol), [1,1′-biphenyl]-4,4′-dicarbaldehyde (**8**, 500 mg, 2.4 mmol) and ammonium acetate (**9**, 426 mg, 5.5 mmol) were dissolved in ethanol (70 mL) and the resulting mixture was refluxed in a pressure vessel for 24 h at 100 °C. The reaction mixture was cooled to 4 °C. Resulting precipitate was filtered off, washed with cold methanol and dried under vacuum to give the desired product **10a** as a yellowish powder (1.08 g, 68%). ^1^H-NMR (300 MHz, DMSO-*d_6_*) δ 8.82 (bs, 2H), 7.43 (d, *J* = 8.4 Hz, 4H), 7.19 (d, *J* = 8.4 Hz, 4H), 4.88 (s, 2H), 4.05–3.94 (m, 8H), 2.27 (s, 12H), 1.14 (t, *J* = 7.1 Hz, 12H) ppm. ^1^H-NMR spectra data were in agreement with data reported in the literature [16].

*Tetradodecyl 4,4′-([1,1′-biphenyl]-4,4′-diyl)bis(2,6-dimethyl-1,4-dihydropyridine-3,5-dicarboxylate)* (**10b**): Dodecyl 3-oxobutanoate (**7b**, 5.19 g, 19.2 mmol), [1,1′-biphenyl]-4,4′-dicarbaldehyde (**8**, 1.00 g, 4.8 mmol) and ammonium acetate (**9**, 0.85 g, 11.0 mmol) were dissolved in ethanol (70 mL) and the resulting mixture was refluxed in a pressure vessel for 72 h at 100 °C. The reaction mixture was concentrated under reduced pressure and the crude product was purified by flash column chromatography with EtOAc/PE, (0:100 to 50:50%) as eluent to give the desired product **10b** as a yellowish solid (2.15 g, 37%). m.p. 133–135 °C. Rf = 0.39 (EtOAc:PE, 1:2). ^1^H-NMR (400 MHz, CDCl_3_) δ 7.41–7.33 (m, 4H), 7.31–7.27 (m, 4H), 5.58 (bs, 2H), 5.02 (s, 2H), 4.03 (t, *J* = 6.7 Hz, 8H), 2.35 (s, 12H), 1.65–1.55 (m, 8H), 1.30–1.23 (m, 72H), 0.90–0.85 (m, 12H) ppm. ^13^C-NMR (101 MHz, CDCl_3_) δ 167.8, 146.5, 144.0, 139.2, 128.3, 126.7, 104.3, 64.1, 39.3, 32.1, 29.9, 29.8, 29.7, 29.6, 29.5, 29.4, 28.9, 26.2, 22.8, 19.8, 14.3 ppm. Anal. calc. for C_78_H_124_N_2_O_8_: C, 76.93; H, 10.26; N, 2.30 found: C, 76.85; H, 10.24; N, 2.47.

### 3.2. Synthesis of Bis(2,6-bis(bromomethyl)-1,4-dihydropyridines ***11a**,**b***

Experimental procedure for the optimization of bromination procedure described in Table 1: *bis*-1,4-DHP **10b** (50 mg, 0.04 mmol) was dissolved in the solvent of choice according to Table 1. The appropriate amount of corresponding brominating agent (**1**, **3**, **6** or **12**) was dissolved in the solvent (25 mL) and added portion-wise to the reaction. The reaction mixture was stirred at r.t. for the time indicated in Table 1. The reaction rate was monitored by TLC and the products were isolated and purified by flash column chromatography using gradient elution with EtOAc/PE, (from 0:100 to 25:75%) to give the desired product **11b.**

*Tetraethyl 4,4′-([1,1′-biphenyl]-4,4′-diyl)bis(2,6-bis(bromomethyl)-1,4-dihydropyridine-3,5-dicarboxylate)* (**11a**): To a stirred solution of compound **10a** (296 mg, 0.45 mmol) in EtOAc (200 mL), a solution of pyridinium bromide–perbromide (**3**, 620 mg, 1.84 mmol) in EtOAc (60 mL) was added dropwise and the resulting reaction mixture was stirred for 30 min at r.t. and monitored by TLC. The reaction mixture was concentrated under reduced pressure and the crude product was purified by flash column chromatography with EtOAc/PE, (0:100 to 40:60%) as eluent to give the desired product **11a** as a yellow solid (161 mg, 38%). m.p. 197–199 °C (decomp.). Rf = 0.36 (EtOAc:PE, 1:3). ^1^H-NMR (400 MHz, DMSO-*d_6_*) δ 9.63 (bs, 2H), 7.50–7.46 (m, 4H), 7.23–7.19 (m, 4H), 4.94 (s, 2H), 4.72 and 4.57 (AB-system, 2d, *J* = 9.7 Hz, 4H and 4H), 4.11–4.05 (m, 8H), 1.18 (t, *J* = 7.1 Hz, 12H) ppm. ^13^C-NMR (101 MHz, DMSO-*d_6_*) δ 179.4, 165.5, 144.2, 138.2, 127.9, 126.4, 103.2, 60.0, 29.5, 27.0, 14.0 ppm. Anal. calc. for C_38_H_40_Br_4_N_2_O_8_: C, 46.94; H, 4.15; N, 2.88 found: C, 46.66; H, 4.13; N, 3.25.

*Tetradodecyl 4,4′-([1,1′-biphenyl]-4,4′-diyl)bis(2,6-bis(bromomethyl)-1,4-dihydropyridine-3,5-dicarboxylate)* (**11b**): To a stirred solution of compound **10b** (500 mg, 0.41 mmol) in EtOAc (200 mL), a solution of pyridinium bromide–perbromide (**3**, 567 mg, 1.68 mmol) in EtOAc (100 mL) was added dropwise and the resulting reaction mixture was stirred for 30 min at r.t. The reaction rate was monitored by TLC. The reaction mixture was concentrated under reduced pressure and the crude product was purified by flash column chromatography with EtOAc/PE, (0:100 to 25:75%) as eluent to give the desired product **11b** as a yellow oil (483 mg, 77%). m.p. 30–32 °C (decomp.). Rf = 0.66 (EtOAc:PE, 1:4). ^1^H-NMR (400 MHz, CDCl_3_) δ 7.42–7.38 (m, 4H), 7.31–7.27 (m, 4H), 6.52 (bs, 2H), 5.06 (s, 2H), 4.93 and 4.63 (AB-system, 2d, *J* = 11.5 Hz, 4H and 4H), 4.09 (t, *J* = 6.7 Hz, 8H), 1.65–1.60 (m, 8H), 1.31–1.24 (m, 72H), 0.91–0.83 (m, 12H) ppm. ^13^C-NMR (101 MHz, CDCl_3_) δ 166.3, 144.5, 141.7, 139.5, 128.3, 126.9, 106.0, 64.9, 39.8, 31.9, 31.9, 29.7, 29.6, 29.5, 29.4, 29.3, 28.6, 27.4, 26.1, 22.7, 14.1 ppm. Anal. calc. for C_78_H_120_Br_4_N_2_O_8_ (with 0.6% hexane): C, 61.22; H, 7.93; N, 1.82 found: C, 61.59; H, 8.07; N, 1.87.

### 3.3. Synthesis of bis-1,4-Dihydropyridine Amphiphiles—General Procedure

Method A: *Bis*(2,6-*b*is(bromomethyl)-1,4-dihydropyridine **11a** or **11b** (1.0 eq.) was dissolved in the corresponding pyridine **13**; **15**–**17** or **18**–**20** and small amounts of solvent were added if needed. The reaction mixture was stirred at r. t. and monitored by TLC. After evaporation the crude product was suspended in acetone and the resulting precipitate was filtered off and washed with cold diethyl ether (compounds **22b**, **24a**,**b**, **25b**, **26a**,**b**, **27a**,**b**) or the crude product was purified by flash column chromatography with saturated (sat.) KNO_3_/H_2_O/Acetone as an eluent (compounds **22a**, **25a**).

Method B: *Bis*(2,6-*b*is(bromomethyl)-1,4-dihydropyridine **11a** or **11b** (1.0 eq.) and pyridine **14** or **21** (4.5 eq.) were dissolved in DMF or acetone. The reaction mixture was stirred at r.t. and monitored by TLC. After evaporation, the crude product was suspended in acetone and the resulting precipitate was filtered off and washed with cold diethyl ether (compound **23b**) or the crude product was purified by flash column chromatography with sat. KNO_3_/H_2_O/Acetone as an eluent (compound **23a**).

*Diethyl 4-[4-[4-[3,5-bis(ethoxycarbonyl)-2,6-bis(pyridin-1-ium-1-ylmethyl)-1,4-dihydropyridin-4-yl]phenyl]phenyl]-2,6-bis(pyridin-1-ium-1-ylmethyl)-1,4-dihydropyridine-3,5-dicarboxylate tetrabromide* (**22a**): Synthesized according to method A. Bromide **11a** (100 mg, 0.10 mmol), pyridine (**13**, 15 mL) and MeOH (6 mL). The reaction mixture was stirred for 2 days. The crude product was purified by flash column chromatography with sat. KNO_3_/H_2_O/Acetone, (1:4:25) as an eluent to give the desired product **22a** as a yellow powder (89 mg, 69%), m.p. 192–194 °C (decomp.). Rf = 0.59 (sat. KNO_3_:H_2_O:Acetone, 1:4:10). MS (+ESI) m/z (relative intensity) 242 (([M − 4Br]/4, 29%). ^1^H-NMR (400 MHz, DMSO-*d_6_*) δ 10.04 (bs, 2H), 8.98–8.92 (m, 8H), 8.59 (t, *J* = 7.7 Hz, 4H), 8.11 (t, *J* = 7.7 Hz, 8H), 7.55 (d, *J* = 8.2 Hz, 4H), 7.38 (d, *J* = 8.2 Hz, 4H), 6.06 and 5.56 (AB-system, 2d, *J* = 15.0 Hz, 4H and 4H), 5.06 (s, 2H), 4.06 (q, *J* = 7.1 Hz, 8H), 1.11 (t, *J* = 7.1 Hz, 12H) ppm. ^13^C-NMR (101 MHz, DMSO-*d_6_*) δ 165.6, 146.4, 144.5, 138.6, 128.3, 128.0, 126.6, 68.7, 60.7, 56.7, 13.9 ppm. HRMS (TOF MS ES+): Calculated [C_58_H_57_N_6_O_8_ − 4H + H]^+^ 965.4238; found: 965.4232.

*Didodecyl 4-[4-[4-[3,5-bis(dodecoxycarbonyl)-2,6-bis(pyridin-1-ium-1-ylmethyl)-1,4-dihydro-pyridin-4-yl]phenyl]phenyl]-2,6-bis(pyridin-1-ium-1-ylmethyl)-1,4-dihydropyridine-3,5-dicarboxylate tetrabromide* (**22b**): Synthesized according to method A. Bromide **11b** (114 mg, 0.06 mmol), pyridine (**13**, 20 mL), MeOH (1 mL). The reaction mixture was stirred for 4 days. The crude product was suspended in acetone and the resulting precipitate was filtered and washed with cold diethyl ether to give the desired product **22b** as a dark yellow solid (75 mg, 54%), m.p. 184–186 °C. Rf = 0.12 (sat. KNO_3_:H_2_O:Acetone, 1:6:10). MS (+ESI) m/z (relative intensity) 382 ([M − 4Br]^+^/4, 100%). ^1^H-NMR (400 MHz, DMSO-*d_6_*) δ 10.57 (bs, 2H), 9.03 (d, *J* = 5.5 Hz, 8H), 8.59 (t, *J* = 7.6 Hz, 4H), 8.12 (t, *J* = 7.6 Hz, 8H), 7.49 (d, *J* = 8.2 Hz, 4H), 7.37 (d, *J* = 8.2 Hz, 4H), 6.16 and 5.65 (AB-system, 2d, *J* = 14.7 Hz, 4H and 4H), 5.06 (s, 2H), 4.01 (t, *J* = 6.1 Hz, 8H), 1.56–1.48 (m, 8H), 1.24–1.20 (m, 72H), 0.85–0.81 (m, 12H) ppm. ^13^C-NMR (101 MHz, DMSO-*d_6_*) δ 165.6, 146.3, 144.7, 138.7, 138.6, 128.2, 128.0, 126.5, 108.3, 64.6, 57.4, 31.3, 29.0, 28.9, 28.7, 28.6, 28.0, 25.6, 22.1, 13.9 ppm. HRMS (TOF MS ES+): Calculated [C_98_H_140_N_6_O_8_]^4+^ 382.2678; found: 382.2668.

*Diethyl 4-[4-[4-[2,6-bis[[4-(dimethylamino)pyridin-1-ium-1-yl]methyl]-3,5-bis(ethoxycarbonyl)-1,4-dihydropyridin-4-yl]phenyl]phenyl]-2,6-bis[[4-(dimethylamino)pyridin-1-ium-1-yl]methyl]-1,4-dihydropyridine-3,5-dicarboxylate tetrabromide* (**23a**): Synthesized according to method B. Bromide **11a** (97 mg, 0.10 mmol), *N,N*-dimethylpyridin-4-amine (**14**, 55 mg, 0.45 mmol), DMF (10 mL). The reaction mixture was stirred for 3 days and monitored by TLC. The crude product was purified by flash column chromatography with sat. KNO_3_/H_2_O/Acetone, (1:4:25) as an eluent to give the desired product **23a** as a yellowish powder (84 mg, 58%), m.p. 195–197 °C (decomp.). Rf = 0.41 (sat. KNO_3_:H_2_O:Acetone, 2:8:25). MS (+ESI) m/z (relative intensity) 282 ([M − 4Br − 2H]^+^/4, 100%). ^1^H-NMR (400 MHz, DMSO-*d_6_*) δ 9.87 (bs, 2H), 8.13 (d, *J* = 7.8 Hz, 8H), 7.54 (d, *J* = 8.3 Hz, 4H), 7.31 (d, *J* = 8.3 Hz, 4H), 6.99 (d, *J* = 7.8 Hz, 8H), 5.56 and 5.13 (AB-system, 2d, *J* = 14.5 Hz, 4H and 4H), 5.02 (s, 2H), 4.08 (q, *J* = 7.1 Hz, 8H), 3.18 (s, 24H), 1.15 (t, *J* = 7.1 Hz, 12H) ppm. ^13^C-NMR (101 MHz, DMSO-*d_6_*) δ 165.7, 155.9, 144.9, 141.6, 140.2, 138.5, 128.2, 126.6, 107.5, 107.1, 60.5, 54.3, 14.0 ppm. HRMS (TOF MS ES+): Calculated [C_66_H_76_N_10_O_8_ − 4H + 2H]^2+^ 569.3002; found: 569.2992.

*Didodecyl 4-[4-[4-[2,6-bis[[4-(dimethylamino)pyridin-1-ium-1-yl]methyl]-3,5-bis(dodecoxy-carbonyl)-1,4-dihydropyridin-4-yl]phenyl]phenyl]-2,6-bis[[4-(dimethylamino)-pyridin-1-ium-1-yl]methyl]-1,4-dihydropyridine-3,5-dicarboxylate tetrabromide* (**23b**): Synthesized according to method B. Bromide **11b** (101 mg, 0.07 mmol), *N,N*-dimethylpyridin-4-amine (**14**, 36 mg, 0.30 mmol), acetone (4 mL). The reaction mixture was stirred for 2 days. The crude product was suspended in acetone and the resulting precipitate was filtered off and washed with cold diethyl ether to give the desired product **23b** as a yellow solid (81 mg, 60%), m.p. 183–185 °C. Rf = 0.68 (sat. KNO_3_:H_2_O:Acetone, 1:4:10). MS (+ESI) m/z (relative intensity) 425 ([M − 4Br]^+^/4, 100%). ^1^H-NMR (400 MHz, DMSO-*d_6_*) δ 10.39 (bs, 2H), 8.25 (d, *J* = 7.7 Hz, 8H), 7.48 (d, *J* = 8.2 Hz, 4H), 7.30 (d, *J* = 8.2 Hz, 4H), 6.98 (d, *J* = 7.7 Hz, 8H), 5.69 and 5.19 (AB-system, 2d, *J* = 14.3 Hz, 4H and 4H), 5.02 (s, 2H), 4.01 (t, *J* = 6.1 Hz, 8H), 3.18 (s, 24H), 1.58–1.49 (m, 8H), 1.25–1.19 (m, 72H), 0.86–0.80 (m, 12H) ppm. ^13^C-NMR (101 MHz, DMSO-*d_6_*) δ 165.7, 155.9, 144.9, 141.7, 140.6, 138.5, 128.0, 126.4, 107.5, 107.0, 64.4, 53.4, 31.3, 29.1, 29.0, 28.8, 28.7, 28.0, 25.7, 22.1, 13.9 ppm. HRMS (TOF MS ES+): Calculated [C_106_H_160_N_10_O_8_]^4+^ 425.3081; found: 425.3100.

*Diethyl 4-[4-[4-[3,5-bis(ethoxycarbonyl)-2,6-bis[(4-propylpyridin-1-ium-1-yl)methyl]-1,4-dihydropyridin-4-yl]phenyl]phenyl]-2,6-bis[(4-propylpyridin-1-ium-1-yl)methyl]-1,4-dihydro-pyridine-3,5-dicarboxylate tetrabromide* (**24a**): Synthesized according to method A. Bromide **11a** (60 mg, 0.06 mmol), pyridine **15** (3 mL), DMF (2 mL). The reaction mixture was stirred for 4 days. The crude product was suspended in acetone and the resulting precipitate was filtered off and washed with cold diethyl ether to give the desired product **24a** as a yellow solid (72 mg, 80%), m.p. 147–149 °C. Rf = 0.46 (sat. KNO_3_:H_2_O:Acetone, 1:4:35). MS (+ESI) m/z (relative intensity) 284 ([M − 4Br]^+^/4, 100%). ^1^H-NMR (400 MHz, DMSO-*d_6_*) δ 10.43 (bs, 2H), 8.89 (d, *J* = 6.3 Hz, 8H), 7.99 (d, *J* = 6.3 Hz, 8H), 7.55 (d, *J* = 7.9 Hz, 4H), 7.37 (d, *J* = 7.9 Hz, 4H), 6.05 and 5.59 (AB-system, 2d, *J* = 14.7 Hz, 4H and 4H), 5.05 (s, 2H), 4.12–4.02 (m, 8H), 2.86 (t, *J* = 7.6 Hz, 8H), 1.66 (sext, *J* = 7.6 Hz, 8H), 1.11 (t, *J* = 7.6 Hz, 12H), 0.89 (t, *J* = 7.0 Hz, 12H) ppm. ^13^C-NMR (101 MHz, DMSO-*d_6_*) δ 165.6, 163.2, 144.6, 143.8, 138.6, 128.3, 127.5, 126.6, 60.6, 57.0, 36.5, 22.6, 13.9, 13.3 ppm. HRMS (TOF MS ES+): Calculated [C_70_H_84_N_6_O_8_]^4+^ 284.1582; found: 284.1596.

*Didodecyl 4-[4-[4-[3,5-bis(dodecoxycarbonyl)-2,6-bis[(4-propylpyridin-1-ium-1-yl)methyl]-1,4-dihydropyridin-4-yl]phenyl]phenyl]-2,6-bis[(4-propylpyridin-1-ium-1-yl)methyl]-1,4-dihydro-pyridine-3,5-dicarboxylate tetrabromide* (**24b**): Synthesized according to method A. Bromide **11b** (40 mg, 0.03 mmol), pyridine **15** (2 mL). The reaction mixture was stirred for 4 days. The crude product was suspended in acetone and the resulting precipitate was filtered off and washed with cold diethyl ether to give the desired product **24b** as a yellow solid (8 mg, 16%), m.p. 183–185 °C (decomp.). Rf = 0.55 (sat. KNO_3_:H_2_O:Acetone, 1:4:35). MS (+ESI) m/z (relative intensity) 424 ([M − 4Br]^+^/4, 100%). ^1^H-NMR (400 MHz, DMSO-*d_6_*) δ 10.60 (bs, 2H), 8.87 (d, *J* = 6.1 Hz, 8H), 7.97 (d, *J* = 6.1 Hz, 8H), 7.48 (d, *J* = 7.9 Hz, 4H), 7.36 (d, *J* = 7.9 Hz, 4H), 6.07 and 5.54 (AB-system, 2d, *J* = 14.5 Hz, 4H and 4H), 5.06 (s, 2H), 4.06–3.95 (m, 8H), 2.85 (t, *J* = 7.5 Hz, 8H), 1.66 (sext, *J* = 7.5 Hz, 8H), 1.55–1.48 (m, 8H), 1.28–1.16 (m, 72H), 0.90 (t, *J* = 7.5 Hz, 12H), 0.85–0.81 (m, 12H) ppm. ^13^C-NMR (101 MHz, DMSO-*d_6_*) δ 165.7, 163.1, 143.8, 128.2, 127.4, 126.4, 64.4, 36.5, 31.3, 29.0, 28.9, 28.7, 28.6, 28.0, 25.6, 22.6, 22.1, 13.9, 13.3 ppm. HRMS (TOF MS ES+): Calculated [C_110_H_164_N_6_O_8_]^4+^ 424.3147; found: 424.3134.

*Diethyl 4-[4-[4-[3,5-bis(ethoxycarbonyl)-2,6-bis[[4-(1-ethylpropyl)pyridin-1-ium-1-yl]-methyl]-1,4-dihydropyridin-4-yl]phenyl]phenyl]-2,6-bis[[4-(1-ethylpropyl)pyridin-1-ium-1-yl]methyl]-1,4-dihydropyridine-3,5-dicarboxylate tetrabromide* (**25a**): Synthesized according to method A. Bromide **11a** (60 mg, 0.06 mmol), pyridine **16** (3 mL), DMF (2 mL). The reaction mixture was stirred for 4 days. The crude product was purified by flash column chromatography with sat. KNO_3_/H_2_O/Acetone (1:4:60) as an eluent to give the desired product **25a** as a yellow solid (58 mg, 58%), m.p. 159–161 °C (decomp.). Rf = 0.38 (sat. KNO_3_:H_2_O:Acetone, 1:4:60). MS (+ESI) m/z (relative intensity) 312 ([M − 4Br]^+^/4, 100%). ^1^H-NMR (400 MHz, DMSO-*d_6_*) δ 10.56 (bs, 2H), 8.90 (d, *J* = 6.3 Hz, 8H), 8.01 (d, *J* = 6.3 Hz, 8H), 7.49 (d, *J* = 7.9 Hz, 4H), 7.31 (d, *J* = 7.9 Hz, 4H), 5.96 and 5.61 (AB-system, 2d, *J* = 14.8 Hz, 4H and 4H), 5.02 (s, 2H), 4.11–3.95 (m, 8H), 2.80–2.74 (m, 4H), 1.80–1.71 (m, 8H), 1.64–1.53 (m, 8H), 1.07 (t, *J* = 7.1 Hz, 12H), 0.70 (m, 24H) ppm. ^13^C-NMR (101 MHz, DMSO-*d_6_*) δ 166.4, 143.9, 128.1, 126.7, 126.4, 60.3, 48.3, 27.4, 27.3, 13.9, 11.6, 11.5 ppm. HRMS (TOF MS ES+): Calculated [C_78_H_100_N_6_O_8_]^4+^ 312.1895; found: 312.1894.

*Didodecyl 4-[4-[4-[3,5-bis(dodecoxycarbonyl)-2,6-bis[[4-(1-ethylpropyl)pyridin-1-ium-1-yl]-methyl]-1,4-dihydropyridin-4-yl]phenyl]phenyl]-2,6-bis[[4-(1-ethylpropyl)pyridin-1-ium-1-yl]methyl]-1,4-dihydropyridine-3,5-dicarboxylate* (**25b**): Synthesized according to method A. Bromide **11b** (47 mg, 0.03 mmol), pyridine **16** (2 mL). The reaction mixture was stirred for 4 days. The crude product was suspended in acetone and the resulting precipitate was filtered off and washed with cold diethyl ether to give the desired product **25b** as a yellow solid (41 mg, 64%), m.p. 152–154 °C (decomp.). Rf = 0.59 (sat. KNO_3_:H_2_O:Acetone, 1:4:60). MS (+ESI) m/z (relative intensity) 452 ([M − 4Br]^+^/4, 100%). ^1^H-NMR (400 MHz, DMSO-*d_6_*) δ 10.50 (bs, 2H), 8.94 (d, *J* = 6.2 Hz, 8H), 8.00 (d, *J* = 6.2 Hz, 8H), 7.45 (d, *J* = 7.9 Hz, 4H), 7.32 (d, *J* = 7.9 Hz, 4H), 6.01 and 5.63 (AB-system, 2d, *J* = 14.6 Hz, 4H and 4H), 5.04 (s, 2H), 4.05–3.91 (m, 8H), 2.83–2.71 (m, 4H), 1.81–1.72 (m, 8H), 1.63–1.55 (m, 8H), 1.52–1.44 (m, 8H), 1.24–1.18 (m, 72H), 0.86–0.80 (m, 12H), 0.71 (t, *J* = 7.3 Hz, 24H) ppm. ^13^C-NMR (101 MHz, DMSO-*d_6_*) δ 166.5, 165.7, 144.0, 128.1, 126.7, 126.3, 64.4, 48.2, 31.3, 29.0, 28.7, 28.0, 27.4, 27.3, 25.6, 22.1, 13.9, 11.6, 11.5 ppm. HRMS (TOF MS ES+): Calculated [C_118_H_180_N_6_O_8_]^4+^ 452.3460; found: 452.3460.

*Diethyl 4-[4-[4-[2,6-bis[(3-bromopyridin-1-ium-1-yl)methyl]-3,5-bis(ethoxycarbonyl)-1,4-dihydropyridin-4-yl]phenyl]phenyl]-2,6-bis[(3-bromopyridin-1-ium-1-yl)methyl]-1,4-dihydro-pyridine-3,5-dicarboxylate tetrabromide* (**26a**): Synthesized according to method A. Bromide **11a** (60 mg, 0.06 mmol), pyridine **17** (2 mL). The reaction mixture was stirred for 2 days. The crude product was suspended in acetone and the resulting precipitate was filtered off and washed with cold diethyl ether to give the desired product **26a** as a yellow solid (79 mg, 80%), m.p. 190–192 °C (decomp.). Rf = 0.22 (sat. KNO_3_:H_2_O:Acetone, 1:4:35). MS (+ESI) m/z (relative intensity) 321 ([M − 4Br]^+^/4, 44%). ^1^H-NMR (400 MHz, DMSO-*d_6_*) δ 10.31 (bs, 2H), 9.40 (s, 4H), 9.13–8.98 (m, 4H), 8.97–8.80 (m, 4H), 8.21–7.97 (m, 4H), 7.58 (d, *J* = 8.3 Hz, 4H), 7.41 (d, *J* = 8.3 Hz, 4H), 6.16 and 5.64 (AB-system, 2d, *J* = 15.0 Hz, 4H and 4H), 5.05 (s, 2H), 4.09 (q, *J* = 7.1 Hz, 8H), 1.15 (t, *J* = 7.1 Hz, 12H) ppm. ^13^C-NMR (101 MHz, DMSO-*d_6_*) δ 165.6, 148.8, 146.2, 144.6, 143.3, 138.6, 137.8, 128.7, 128.4, 126.7, 121.7, 108.5, 60.7, 57.7, 13.9 ppm. Anal. calc. for C_58_H_56_Br_8_N_6_O_8_: C, 43.34; H, 3.70; N, 5.23 found: C, 43.09; H, 4.01; N, 4.96.

*Didodecyl 4-[4-[4-[2,6-bis[(3-bromopyridin-1-ium-1-yl)methyl]-3,5-bis(dodecoxycarbonyl)-1,4-dihydropyridin-4-yl]phenyl]phenyl]-2,6-bis[(3-bromopyridin-1-ium-1-yl)methyl]-1,4-dihydro-pyridine-3,5-dicarboxylate tetrabromide* (**26b**): Synthesized according to method A. Bromide **11b** (53 mg, 0.03 mmol), pyridine **17** (2 mL). The reaction mixture was stirred for 4 days. The crude product was suspended in acetone and the resulting precipitate was filtered off and washed with cold diethyl ether to give the desired product **26b** as a yellow solid (45 mg, 62%), m.p. 156–158 °C (decomp.). Rf = 0.21 (sat. KNO_3_:H_2_O:Acetone, 1:4:35). MS (+ESI) m/z (relative intensity) 461 ([M − 4Br]^+^/4, 23%). ^1^H-NMR (400 MHz, DMSO-*d_6_*) δ 10.32 (bs, 2H), 9.37 (s, 4H), 9.01 (d, *J* = 6.0 Hz, 4H), 8.89 (d, *J* = 8.3 Hz, 4H), 8.10–8.02 (m, 4H), 7.51 (d, *J* = 7.8 Hz, 4H), 7.39 (d, *J* = 7.8 Hz, 4H), 6.16 and 5.60 (AB-system, 2d, *J* = 14.7 Hz, 4H and 4H), 5.04 (s, 2H), 4.07–3.96 (m, 8H), 1.56–1.49 (m, 8H), 1.24–1.18 (m, 72H), 0.87–0.79 (m, 12H) ppm. ^13^C-NMR (101 MHz, DMSO-*d_6_*) δ 165.7, 148.8, 146.1, 144.6, 143.4, 138.6, 128.7, 128.3, 126.4, 121.7, 108.5, 64.6, 57.6, 31.3, 29.0, 29.0, 28.7, 28.6, 28.0, 25.6, 22.1, 13.9 ppm. Anal. calc. for C_98_H_136_Br_8_N_6_O_8_ (with 1.2% water): C, 53.69; H, 6.39; N, 3.83 found: C, 53.33; H, 6.39; N, 3.81.

*Diethyl 4-[4-[4-[3,5-bis(ethoxycarbonyl)-2,6-bis[[4-(3-phenylpropyl)pyridin-1-ium-1-yl]methyl]-1,4-dihydropyridin-4-yl]phenyl]phenyl]-2,6-bis[[4-(3-phenylpropyl)pyridin-1-ium-1-yl]methyl]-1,4-dihydropyridine-3,5-dicarboxylate tetrabromide* (**27a**): Synthesized according to method A. Bromide **11a** (31 mg, 0.03 mmol), pyridine **18** (3 mL). The reaction mixture was stirred for 4 days. The crude product was suspended in hexane and the resulting precipitate was filtered off and washed with cold diethyl ether to give the desired product **27a** as a yellowish solid (40 mg, 71%), m.p. 169–171 °C (decomp.). Rf = 0.57 (sat. KNO_3_:H_2_O:Acetone, 1:4:50). MS (+ESI) m/z (relative intensity) 360 ([M − 4Br]^+^/4, 100%). ^1^H-NMR (600 MHz, DMSO-*d_6_*) δ 10.43 (bs, 2H), 8.89 (d, *J* = 6.3 Hz, 8H), 7.99 (d, *J* = 6.3 Hz, 8H), 7.54 (d, *J* = 7.7 Hz, 4H), 7.37 (d, *J* = 7.7 Hz, 4H), 7.30–7.24 (m, 8H), 7.21–7.15 (m, 12H), 6.05 and 5.60 (AB-system, 2d, *J* = 14.7 Hz, 4H and 4H), 5.05 (s, 2H), 4.09–4.03 (m, 8H), 2.90–2.81 (m, 8H), 2.60–2.55 (m, 8H), 1.97–1.88 (m, 8H), 1.11 (t, *J* = 7.1 Hz, 12H) ppm. ^13^C-NMR (151 MHz, DMSO-*d_6_*) δ 165.5, 163.0, 149.5, 144.6, 143.8, 141.1, 138.4, 128.4, 128.3, 128.2, 127.5, 126.7, 126.0, 123.9, 108.3, 60.6, 56.9, 34.5, 34.4, 30.7, 13.9 ppm. HRMS (TOF MS ES+): Calculated [C_94_H_100_N_6_O_8_]^4+^ 360.1895; found: 360.1902.

*Didodecyl 4-[4-[4-[3,5-bis(dodecoxycarbonyl)-2,6-bis[[4-(3-phenylpropyl)pyridin-1-ium-1-yl]methyl]-1,4-dihydropyridin-4-yl]phenyl]phenyl]-2,6-bis[[4-(3-phenylpropyl)pyridin-1-ium-1-yl]methyl]-1,4-dihydropyridine-3,5-dicarboxylate tetrabromide* (**27b**): Synthesized according to method A. Bromide **11b** (54 mg, 0.04 mmol), pyridine **18** (3 mL). The reaction mixture was stirred for 4 days. The crude product was suspended in acetone and the resulting precipitate was filtered off and washed with cold diethyl ether to give the desired product **27b** as a yellowish solid (47 mg, 57%), m.p. 215–217 °C (decomp.). Rf = 0.40 (sat. KNO_3_:H_2_O:Acetone, 1:4:60). MS (+ESI) m/z (relative intensity) 500 ([M − 4Br]^+^/4, 53%). ^1^H-NMR (400 MHz, DMSO-*d_6_*) δ 10.51 (bs, 2H), 8.89 (d, *J* = 6.2 Hz, 8H), 7.98 (d, *J* = 6.2 Hz, 8H), 7.49 (d, *J* = 7.9 Hz, 4H), 7.37 (d, *J* = 7.9 Hz, 4H), 7.31–7.24 (m, 8H), 7.22–7.15 (m, 12H), 6.07 and 5.58 (AB-system, 2d, *J* = 14.7 Hz, 4H and 4H), 5.07 (s, 2H), 4.06–3.95 (m, 8H), 2.89–2.82 (m, 8H), 2.62–2.55 (m, 8H), 1.93 (p, *J* = 7.8 Hz, 8H), 1.55–1.46 (m, 8H), 1.24–1.18 (m, 72H), 0.86–0.81 (m, 12H) ppm. ^13^C-NMR (101 MHz, DMSO-*d_6_*) δ 165.6, 162.9, 143.8, 141.1, 128.3, 128.2, 127.4, 126.4, 126.0, 64.5, 34.5, 34.4, 31.3, 30.7, 29.1, 29.0, 28.7, 28.6, 28.0, 25.6, 22.1, 13.9 ppm. HRMS (TOF MS ES+): Calculated [C_134_H_180_N_6_O_8_]^4+^ 500.3460; found: 500.3468.

### 3.4. Self-Assembling Properties of Compounds by Dynamic Light Scattering Measurements

The self-assembling properties of amphiphilic bis-1,4-DHPs **22**–**27** were determined according to a previously published procedure with minor modifications [9,34]. Briefly, samples were prepared by making stock solutions of compounds **22**–**27** in EtOH at a concentration of 4 mM. Stock solutions of the compounds (250 μL, 4 mM in EtOH 96%) were injected into deionized water (1.75 mL) with vigorous stirring (IKA Vortex 2 (IKA, Staufen, Germany)) to produce samples with a final compound concentration of 0.5 mM. In the case of compound **26a,** DMSO was used as a solvent for stock solution and stock solutions of compounds **26b** and **27a**,**b** were prepared at concentration of 2 mM. Liposomes dispersed in water were homogenized using 20 passes in a hand-held, small-scale extruder (Avestin Europe GmbH, Mannheim, Germany) with polycarbonate nucleopore filters (100 nm, Whatman, Frisenette, Knebel, Denmark). The DLS measurements of the prepared aqueous solutions of the nanoparticles were carried out on a Zetasizer Nano ZSP (Malvern Panalytical Ltd., Malvern, UK) instrument with Malvern Instruments Ltd. Software 8.01.4906, using the following specifications: medium: water; refractive index: 1.33; viscosity: 0.8872 cP; temperature: 25 °C; dielectric constant: 78.5; nanoparticles: liposomes; refractive index of materials: 1.60; detection angle: 173°; wavelength: 633 nm. Data were analyzed using the multimodal number distribution software that was included with the instrument. The measurements were performed in quadruplicate in order to check their reproducibility.

## 4. Conclusions

The synthesis of cationic *bis*-1,4-dihydropyridine derivatives was achieved using Hantzsch synthesis followed by bromination and nucleophilic substitution of bromine with various pyridines. The reaction conditions for the bromination of the methyl groups of *bis*-1,4-dihydropyridines were optimized. The highest yields were obtained using pyridinium bromide–perbromide in ethyl acetate. However, the limiting step for the synthesis of corresponding amphiphiles from *bis*-1,4-DHPs is not a bromination step, as it was in the case of 1,4-DHPs, but bromine nucleophilic substitution due to the relatively low solubility of tetra(bromomethyl)-1,4-DHPs. By nucleophilic substitution of bromine with various pyridine derivatives, 12 original amphiphilic *bis*-1,4-DHPs were obtained. *Bis*-1,4-DHP derivatives possessing short alkyl ester chains at positions 3 and 5 react more readily than ones containing lengthy alkyl ester chains. It was demonstrated that under the studied conditions, nucleophilic substitution of bromine was not observed when it was carried out with pyridines containing electron-withdrawing or sterically hindered substituents. 

The self-assembling properties of the synthesized amphiphilic *bis*-1,4-DHP were analyzed, and it was determined that the average diameter of freshly prepared nanoparticles was in a range from 61 to 3111 nm for non-extruded samples and from 73 to 1039 nm for extruded samples. However, based on the obtained surface charge values, it can be suggested that stability of particles formed by *bis*-1,4-DHP amphiphiles was dependent on the structure of amphiphile.

## Figures and Tables

**Table 1:** Studies of conditions for bromination of *bis*-1,4-dihydropyridine **10b** varying brominating agents and solvents.

Entry	Reagent	Eq.	Solvent	Concentration, mM ^1^	Reaction Time, h	Yield, % ^2^
1	NBS (**1**)	4.1	MeOH	0.23	48	41
2			CHCl_3_	1.17	3	54
3			CHCl_3_/MeOH (1:3)	1.17	24	48
4			EtOAc/MeOH (1:3)	0.68	20	56
5			EtOAc	0.68	24	73
6	DBDMH (**6**)	2.1	MeOH	0.23	48	22
7			CHCl_3_	1.17	2	-
8			EtOAc/MeOH (1:3)	0.68	20	48
9	PBPB (**3**)	4.1	EtOAc/MeOH (1:3)	0.68	0.5	50
10		4.1	EtOAc/MeOH (1:3)	0.68	2	48
11		4.1	EtOAc/MeOH (1:3)	0.68	4	35
12		5.0	EtOAc/MeOH (1:3)	0.68	2	33
13		4.1	EtOAc	0.68	0.5	85
14	PTAB (**12**)	4.1	EtOAc	0.68	0.5	59

^1^ *bis*-1,4-DHP concentration in solution; ^2^ Isolated yield; - Product was not obtained.

**Table 2:** Reagents and reaction conditions for the synthesis of *bis*-1,4-dihydropyridine amphiphile **22a**,**b**–**27a**,**b**.

Entry	R ^1^	Comp.	Eq.	R	Solvent	Reaction Time, Days	Product	Yield, % ^1^
1	H	**13**	Excess	C_2_H_5_	MeOH	2	**22a**	69
2				C_12_H_25_		2	**22b**	54
3	4-N(CH_3_)_2_	**14**	4.5	C_2_H_5_	DMF	2	**23a**	58
4				C_12_H_25_	Acetone	2	**23b**	60
5	4-C_3_H_7_-n	**15**	Excess	C_2_H_5_	DMF	4	**24a**	80
6				C_12_H_25_	-	4	**24b**	16
7	4-CH(C_2_H_5_)_2_	**16**	Excess	C_2_H_5_	DMF	4	**25a**	58
8				C_12_H_25_	-	4	**25b**	64
9	3-Br	**17**	Excess	C_2_H_5_	-	2	**26a**	80
10				C_12_H_25_		4	**26b**	62
11	4-(CH_2_)_3_Ph	**18**	Excess	C_2_H_5_	DMF	4	**27a**	71
12				C_12_H_25_	-	4	**27b**	57
13	4-Ph	**19**	4.1	C_2_H_5_	DMF	6	-	-
14				C_12_H_25_		8	-	-
15	4-CH_2_Ph	**20**	Excess	C_2_H_5_	DMF	4	-	-
16				C_12_H_25_	-	4	-	-
17	4-CONH_2_	**21**	4.1	C_2_H_5_	DMF	6	-	-
18				C_12_H_25_		12	-	-

^1^ Isolated yields are given; - Product was not obtained.

**Table 3:** Values of the hydrodynamic average diameter (Z_av_), polydispersity index (PDI) and zeta-potential (Z_pot_) of nanoparticles formed by pyridinium salts **22**–**27** in an aqueous solution obtained by DLS measurements.

Entry	Comp.	Freshly Prepared		3 Days			7 Days		14 Days	
		Z_av_ D_h_, nm	PDI	Z_av_ D_h_, nm	PDI	Z-Potential, mV	Z_av_ D_h_, nm	PDI	Z_av_ D_h_, nm	PDI
1 *	**22a**	61 ± 1	0.205 ± 0.014	100 ± 1	0.368 ± 0.013	24.6 ± 0.5	70 ± 1	0.263 ± 0.014	71 ± 1	0.269 ± 0.029
2	**E-22b**	353 ± 66	0.384 ± 0.019	293 ± 63	0.488 ± 0.150	31.0 ± 1.4	264 ± 29	0.410 ± 0.337	249 ± 36	0.476 ± 0.096
3	**E-23a**	73 ± 8	0.391 ± 0.062	140 ± 6	0.612 ± 0.016	24.8 ± 0.5	205 ± 10	0.639 ± 0.051	326 ± 10	0.481 ± 0.090
4	**E-23b**	897 ± 186	0.691 ± 0.114	261 ± 75	0.358 ± 0.089	18.0 ± 1.9	26 ± 4	1 ± 0	37 ± 8	1 ± 0
5	**E-24a**	1096 ± 224	0.784 ± 0.194	596 ± 139	0.397 ± 0.187	10.9 ± 0.4	618 ± 72	0.546 ± 0.212	585 ± 130	0.707 ± 0.065
6	**E-25a**	302 ± 1	0.478 ± 0.035	502 ± 41	0.277 ± 0.084	9.7 ± 0.8	757 ± 65	0.647 ± 0.546	510 ± 199	0.342 ± 0.067
7	**E-25b**	653 ± 130	0.542 ± 0.089	495 ± 133	0.486 ± 0.095	24.9 ± 1.1	436 ± 143	0.669 ± 0.276	324 ± 248	0.742 ± 0.312
8	**E-26a**	557 ± 27	0.546 ± 0.030	332 ± 80	0.439 ± 0.075	23.4 ± 1.1	766 ± 102	0.645 ± 0.084	305 ± 28	0.601 ± 0.331
9	**E-26b**	655 ± 218	0.928 ± 0.074	236 ± 50	0.555 ± 0.293	11.9 ± 0.9	185 ± 28	0.527 ± 0.323	102 ± 17	0.789 ± 0.123
10	**E-27a**	380 ± 36	0.372 ± 0.114	401 ± 16	0.151 ± 0.114	20.9 ± 1.0	321 ± 29	0.334 ± 0.048	259 ± 7	0.269 ± 0.026
11	**E-27b**	427 ± 26	0.395 ± 0.052	434 ± 3	0.357 ± 0.126	23.8 ± 0.2	444 ± 5	0.289 ± 0.052	- #	- #

* non-extruded sample; - measurement was not performed; # aggregation.

## Data Availability

The data presented in this study are available in this article.

## Supplementary Materials

The following supporting information can be downloaded at: https://www.mdpi.com/article/10.3390/molecules29010161/s1, Characterization data for products **10a**,**b**, **11a**,**b**, **22a**,**b**–**27a**,**b**; including ^1^H- and ^13^C-NMR spectra and HRMS. Figure S1: for comp. **10a**; Figures S2 and S3: for comp. **10b**; Figures S4 and S5: for comp. **11a**; Figures S6 and S7: for comp. **11b**; Figures S8–S10 for comp. **22a**; Figure S11–S13 for comp. **22b**; Figures S14–S16 for comp. **23a**; Figures S17–S19 for comp. **23b**; Figures S20–S22 for com. **24a**; Figures S23–S25 for comp. **24b**; Figures S26–S28 for com. **25a**; Figures S29–S31 for comp. **25b**; Figures S32 and S33 for comp. **26a**; Figures S34 and S35 for comp. **26b**; Figures S36–S38 for comp. **27a**; Figures S39–S41 for comp. **27b**; Table S1: dynamic light scattering measurement results of non-extruded samples of compounds **22a**,**b**–**27a**,**b**; Figures S42–S46: graphs of DLS measurements for comp. **22a**. Other raw DLS measurement data is added as a separate Zetasizer measurement file.
