# Supplementary material for: Development of Self-Assembling bis-1,4-Dihydropyridines: Detailed Studies of Bromination of Four Methyl Groups and Bromine Nucleophilic Substitution
Source: Molecules. 2023 Dec 27;29(1):161. doi: 10.3390/molecules29010161 (PMC10779897; doi:10.3390/molecules29010161)
Supplement: Supplementary file 1 [file molecules-29-00161-s001.zip › molecules-2775751-Supplementary data.docx]

*Supplementary Materials*

Development of self-assembling *bis*-1,4-dihydropyridines: detailed studies of bromination of four methyl groups and bromine nucleophilic substitution

**Martins Kaukulis ^1,2,^*, Martins Rucins ^1^, Davis Lacis ^1,2^, Aiva Plotniece ^1,3^, Arkadij Sobolev ^1^**

^1^ Latvian Institute of Organic Synthesis, Aizkraukles Str. 21, Riga LV-1006, Latvia; martins.kaukulis@osi.lv (M.K.), rucins@osi.lv (M.R.); davis.lacis@osi.lv (D.L.), aiva@osi.lv (A.P.), arkady@osi.lv (A.S.)

^2^ Faculty of Materials Science and Applied Chemistry, Riga Technical University, P. Valdena Str. 3, Riga LV-1048, Latvia;

^3^ Department of Pharmaceutical Chemistry, Faculty of Pharmacy, Riga Stradiņš University, Konsula 21, LV-1007, Riga, Latvia;

***** Correspondence: martins.kaukulis@osi.lv; Tel.: +371-67014852

Table of Contents

Characterization data of compound **10a** 3

Characterization data of compound **10b** 3

Characterization data of compound **11a** 4

Characterization data of compound **11b** 5

Characterization data of compound **22a** 6

Characterization data of compound **22b** 8

Characterization data of compound **23a** 9

Characterization data of compound **23b** 12

Characterization data of compound **24a** 14

Characterization data of compound **24b** 16

Characterization data of compound **25a** 18

Characterization data of compound **25b** 20

Characterization data of compound **26a** 22

Characterization data of compound **26b** 23

Characterization data of compound **27a** 24

Characterization data of compound **27b** 26

Dynamic light scattering measurement results of non-extruded samples 28

Graphs of the dynamic light scattering measurement results of compound **22a** 29


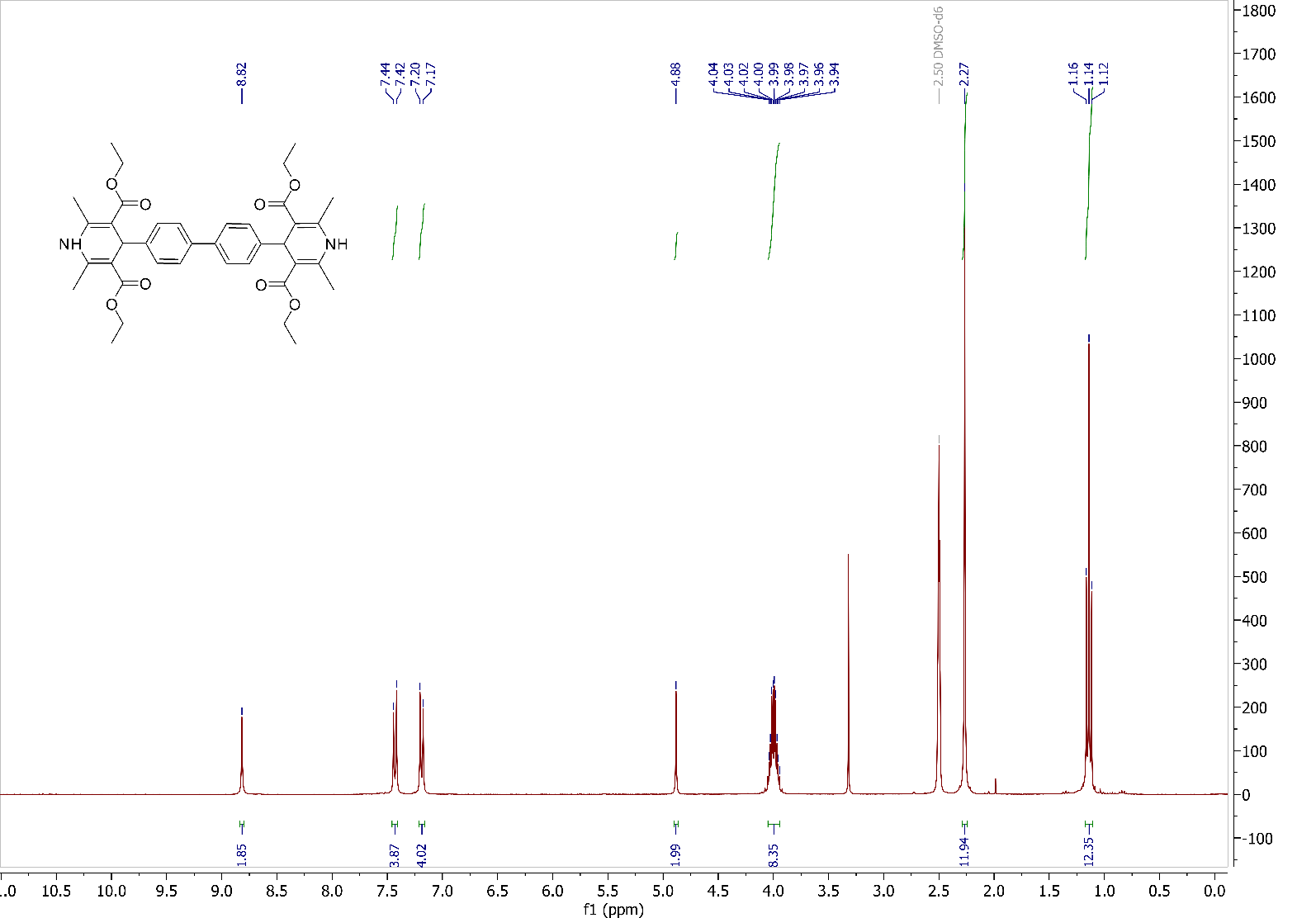


**Figure S1.** ^1^H NMR spectrum of tetraethyl 4,4'-([1,1'-biphenyl]-4,4'-diyl)*bis*(2,6-dimethyl-1,4-dihydropyridine-3,5-dicarboxylate) (**10a**).


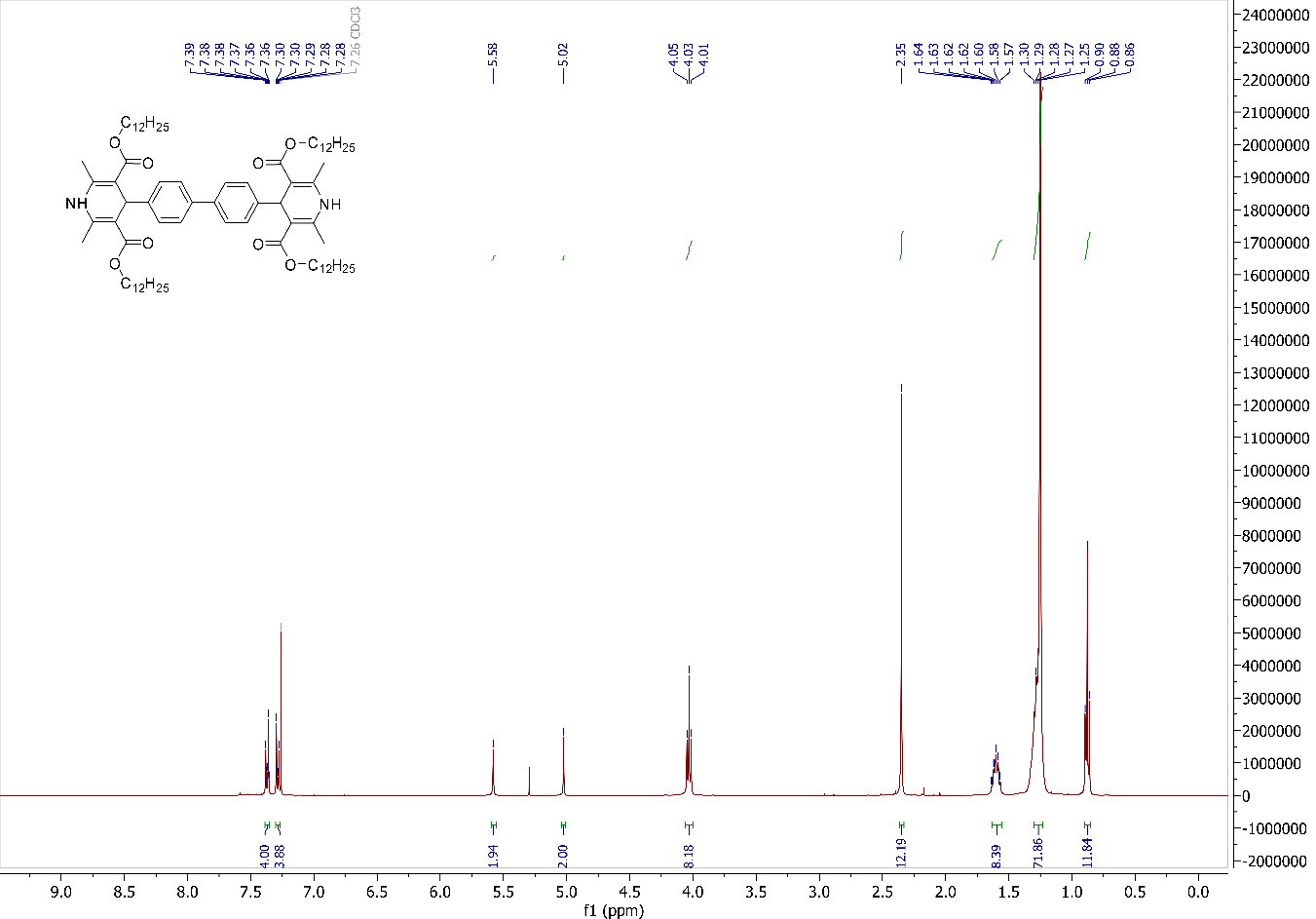


**Figure S2.** ^1^H NMR spectrum of tetradodecyl 4,4'-([1,1'-biphenyl]-4,4'-diyl)*bis*(2,6-dimethyl-1,4-dihydropyridine-3,5-dicarboxylate) (**10b**).


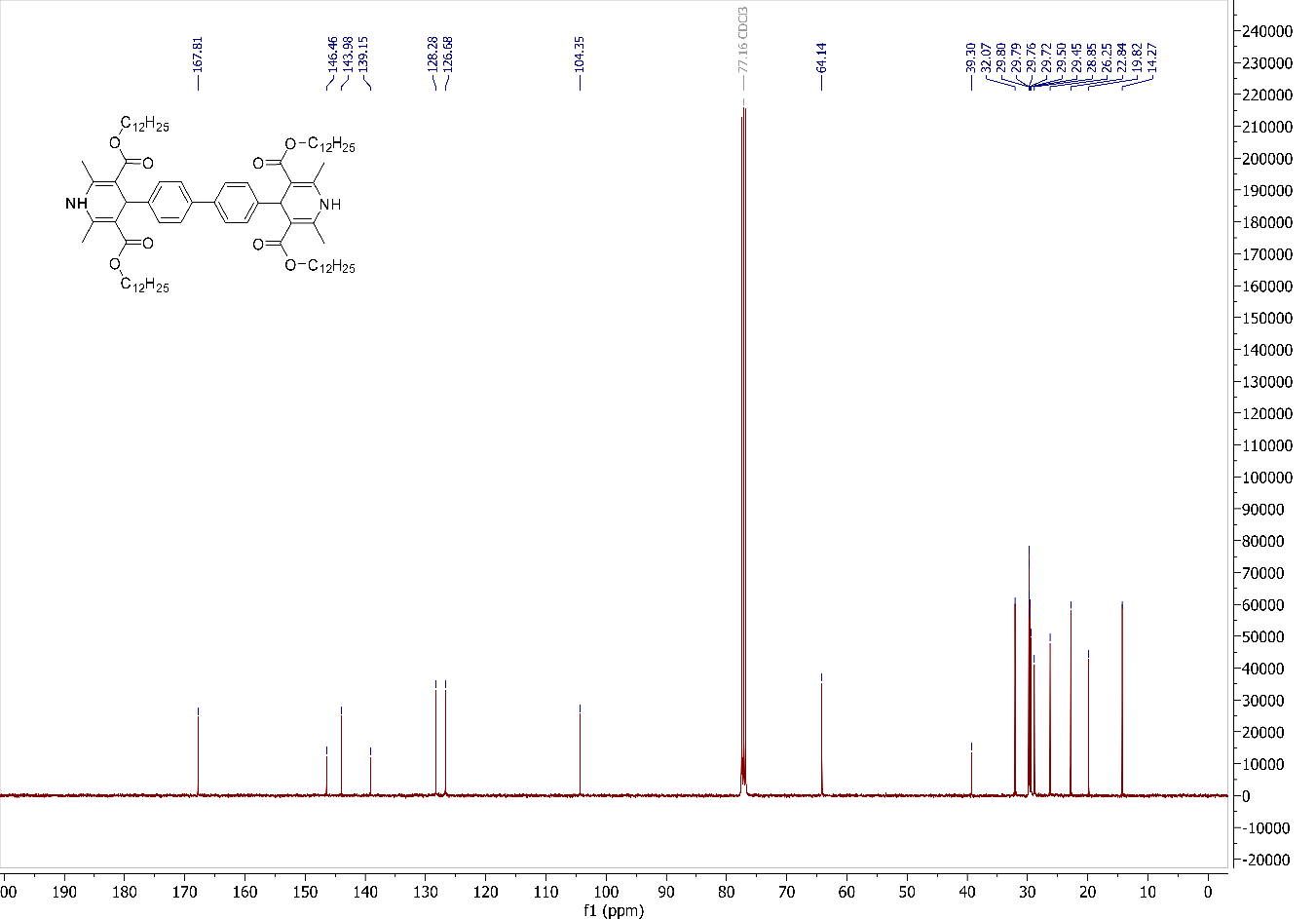


**Figure S3.** ^13^C NMR spectrum of tetradodecyl 4,4'-([1,1'-biphenyl]-4,4'-diyl)*bis*(2,6-dimethyl-1,4-dihydropyridine-3,5-dicarboxylate) (**10b**).


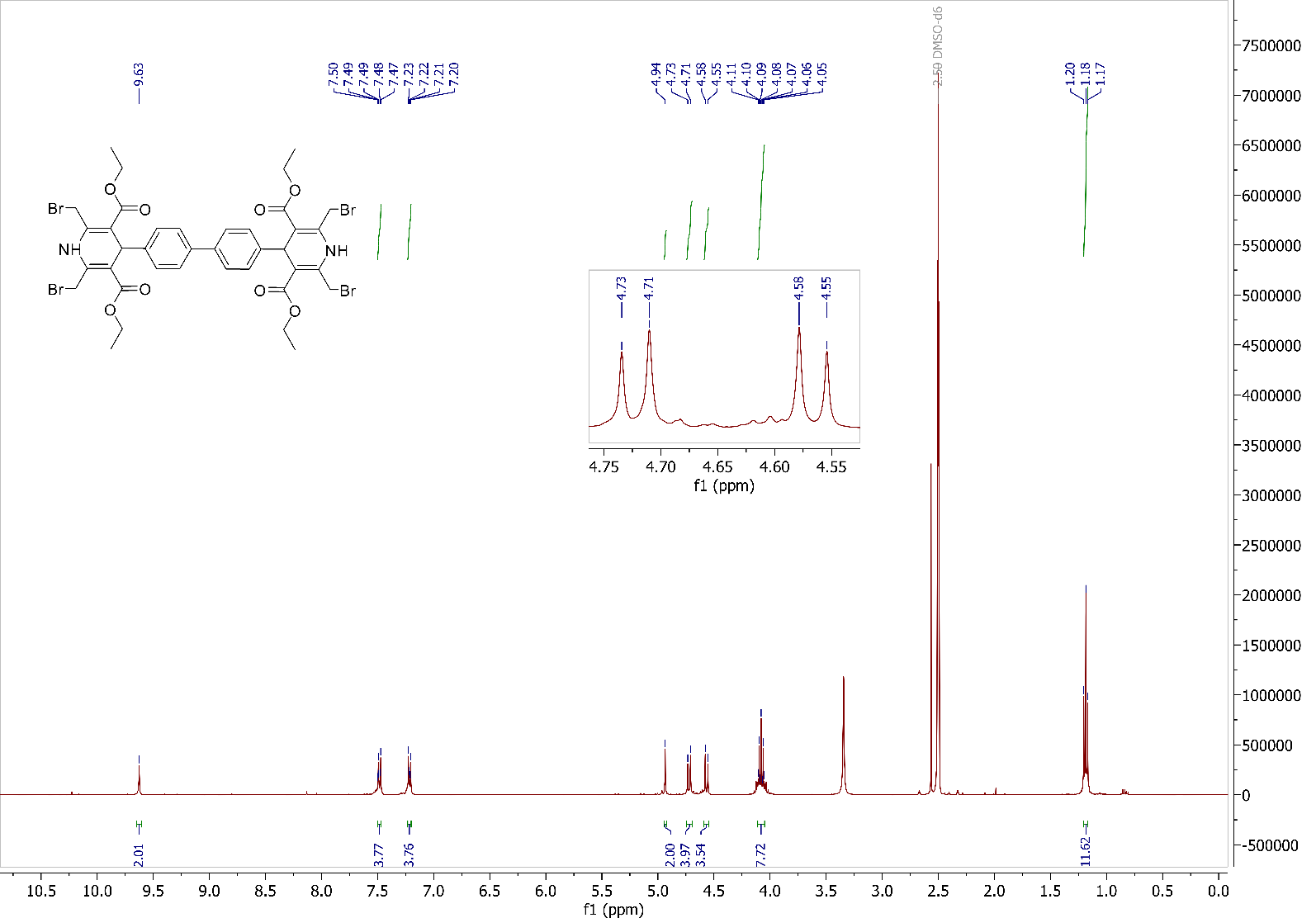


**Figure S4.** ^1^H NMR spectrum of tetraethyl 4,4'-([1,1'-biphenyl]-4,4'-diyl)*bis*(2,6-*bis*(bromomethyl)-1,4-dihydropyridine-3,5-dicarboxylate) (**11a**).


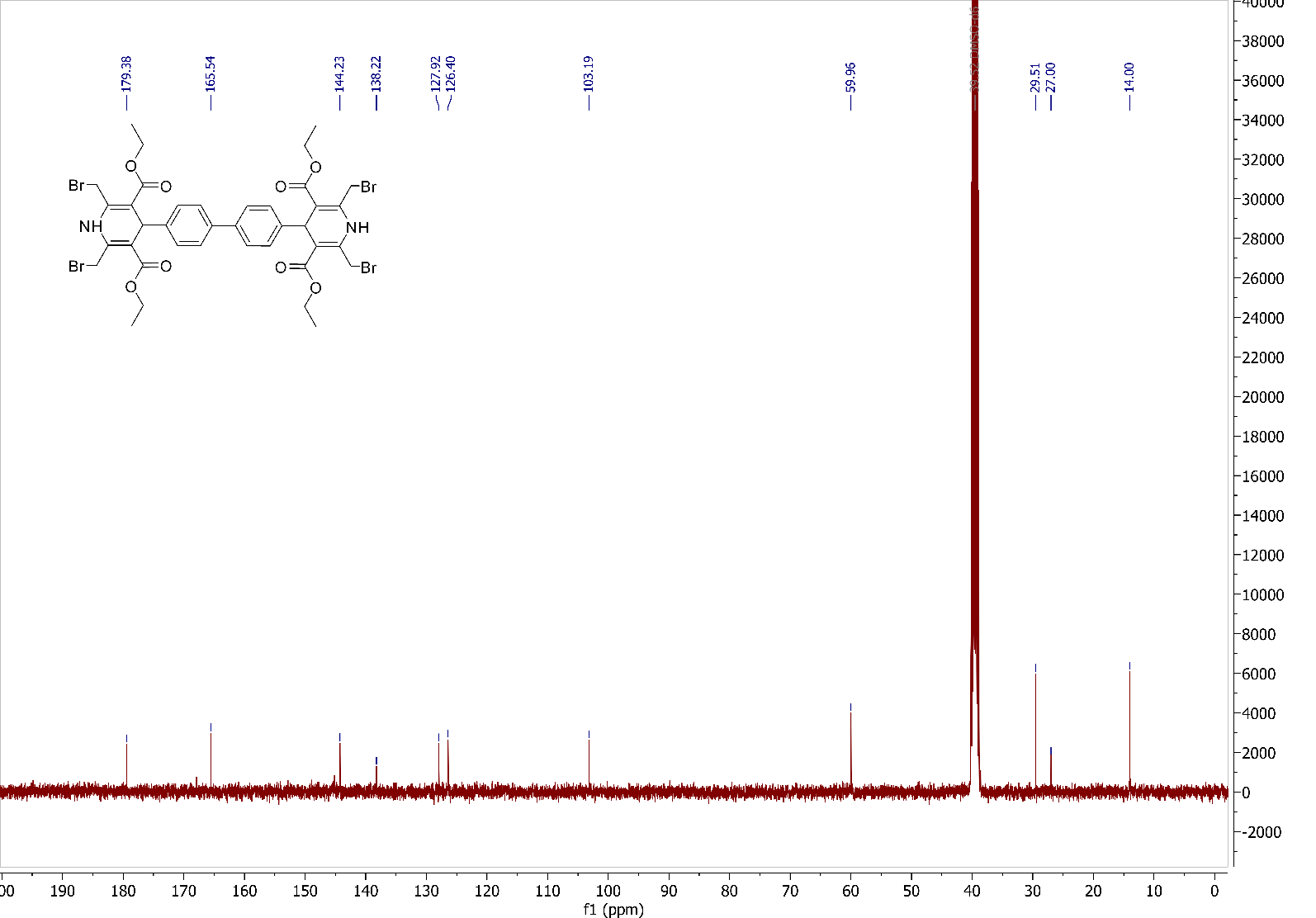


**Figure S5.** ^13^C NMR spectrum of tetraethyl 4,4'-([1,1'-biphenyl]-4,4'-diyl)*bis*(2,6-*bis*(bromomethyl)-1,4-dihydropyridine-3,5-dicarboxylate) (**11a**).


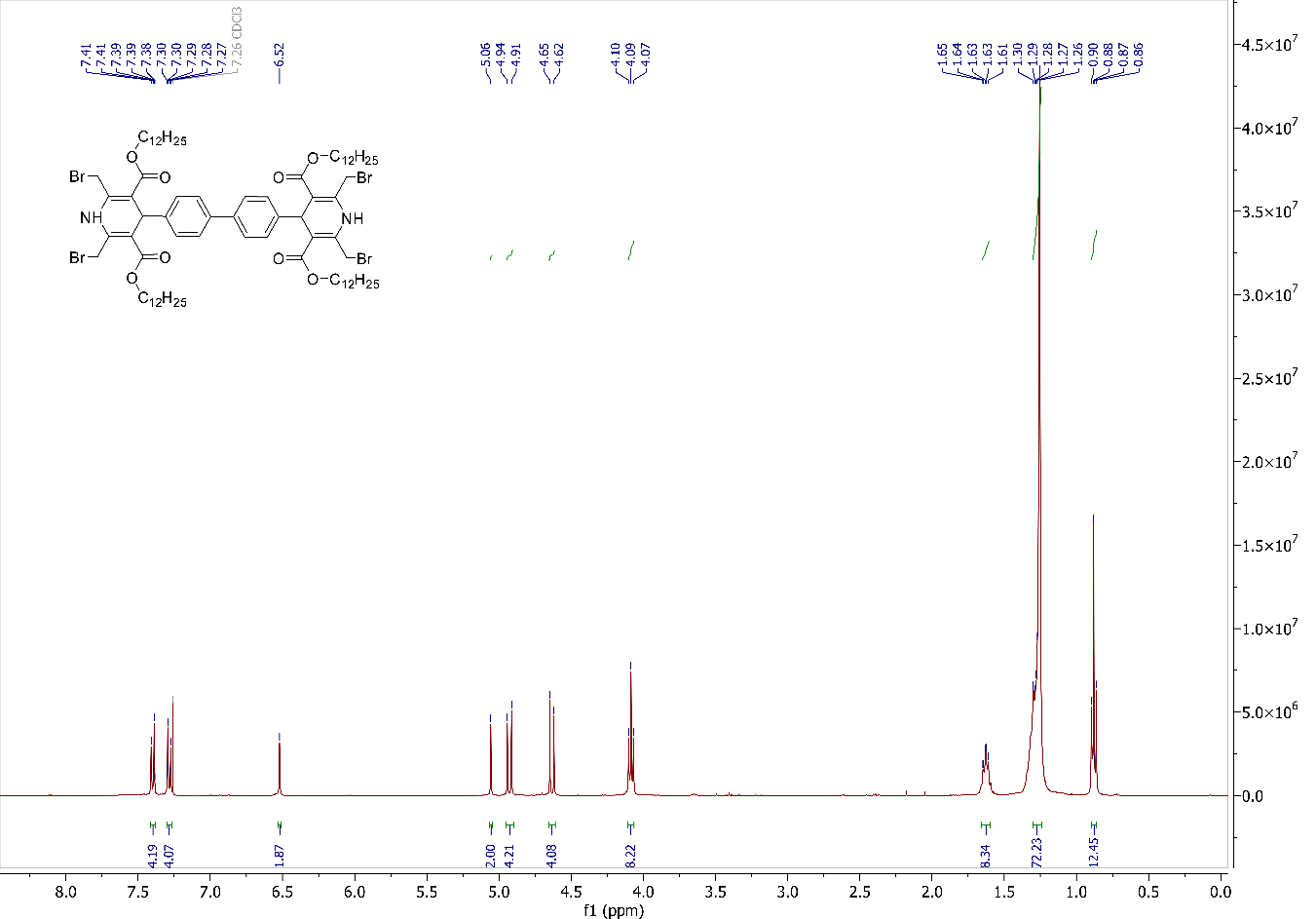


**Figure S6.** ^1^H NMR spectrum of tetradodecyl 4,4'-([1,1'-biphenyl]-4,4'-diyl)*bis*(2,6-*bis*(bromomethyl)-1,4-dihydropyridine-3,5-dicarboxylate) (**11b**).

**
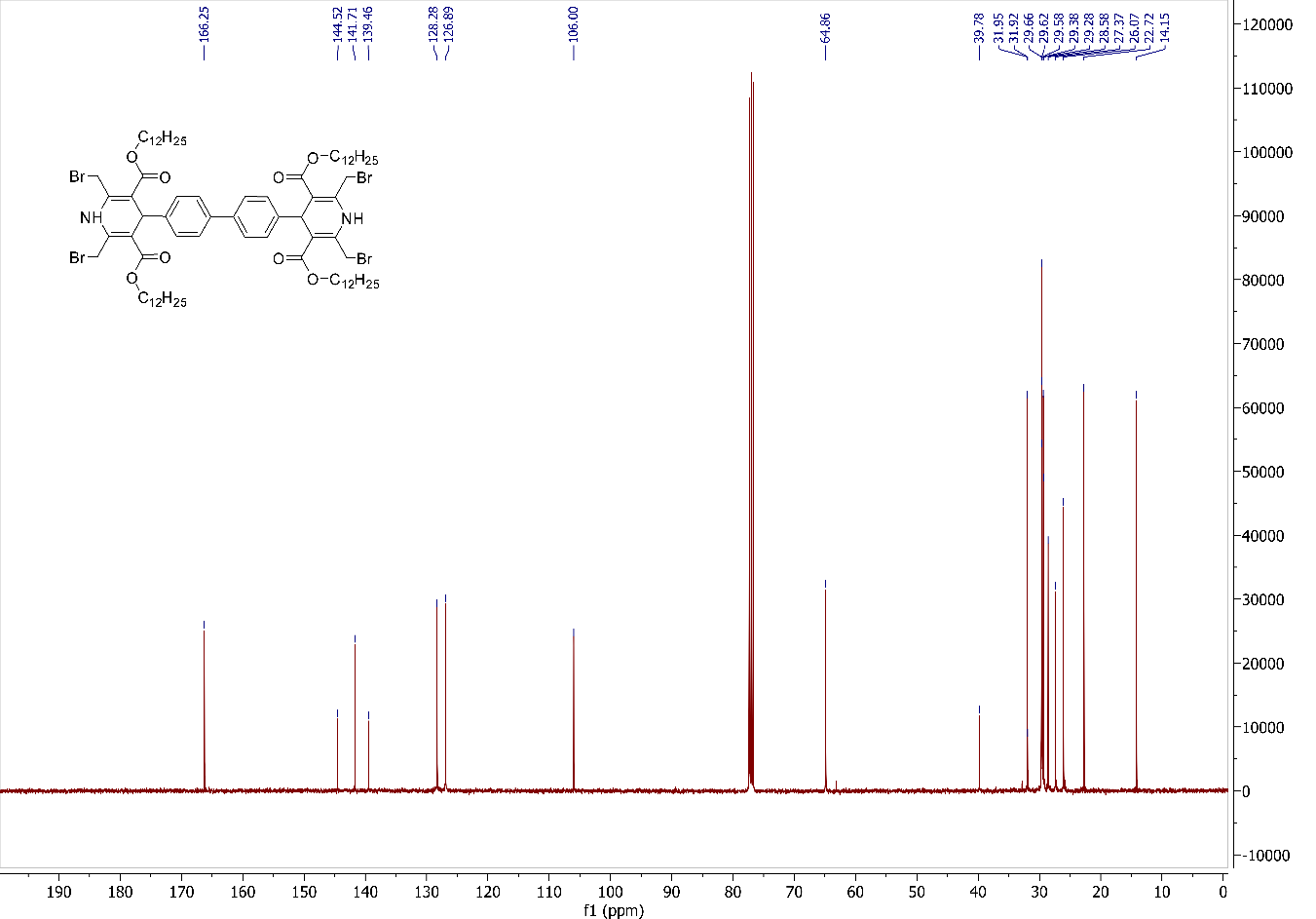
**

**Figure S7.** ^13^C NMR spectrum of tetradodecyl 4,4'-([1,1'-biphenyl]-4,4'-diyl)*bis*(2,6-*bis*(bromomethyl)-1,4-dihydropyridine-3,5-dicarboxylate) (**11b**).


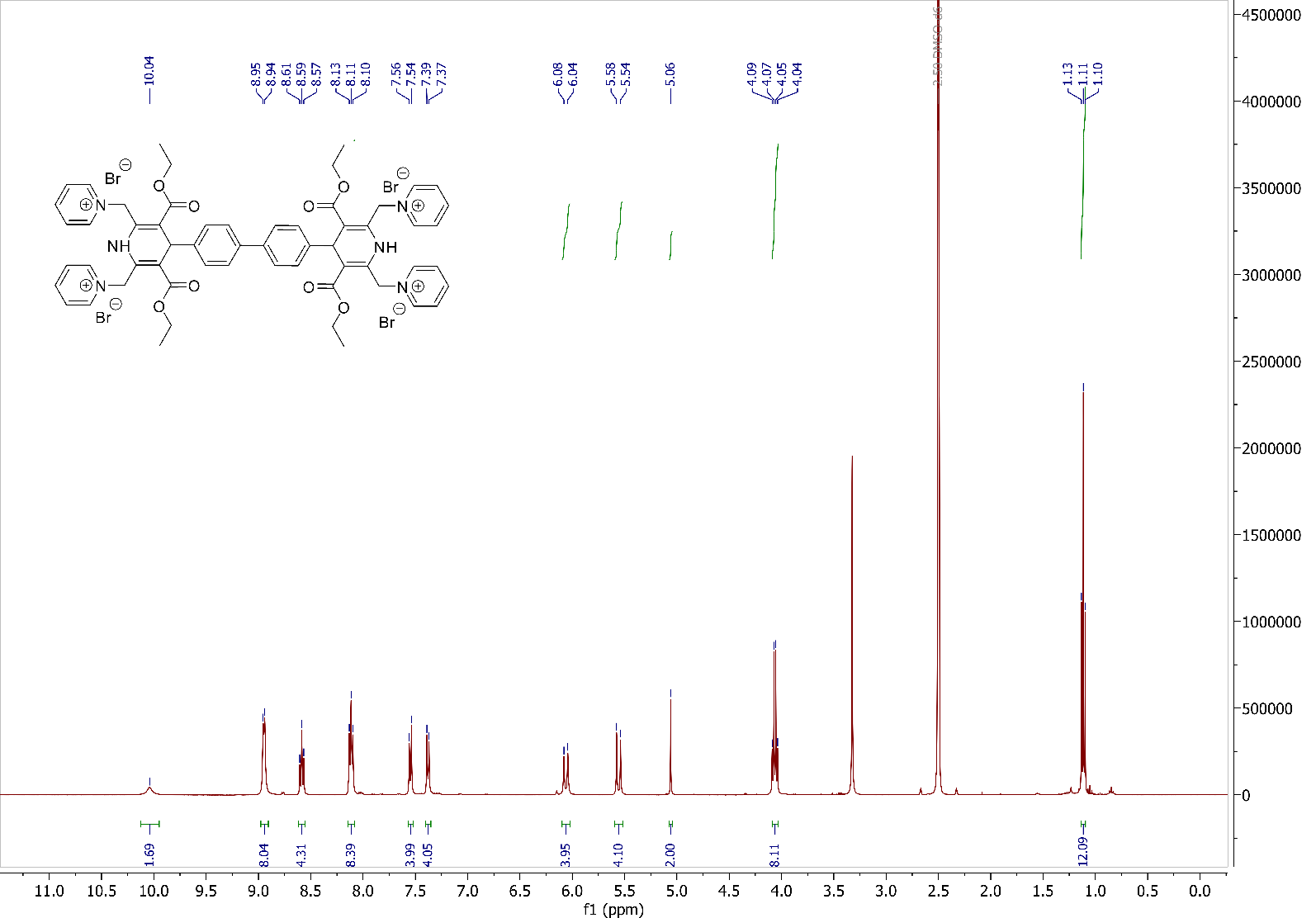


**Figure S8.** ^1^H NMR spectrum of diethyl 4-[4-[4-[3,5-*bis*(ethoxycarbonyl)-2,6-*bis*(pyridin-1-ium-1-ylmethyl)-1,4-dihydropyridin-4-yl]phenyl]phenyl]-2,6-*bis*(pyridin-1-ium-1-ylmethyl)-1,4-dihydropyridine-3,5-dicarboxylate tetrabromide (**22a**).


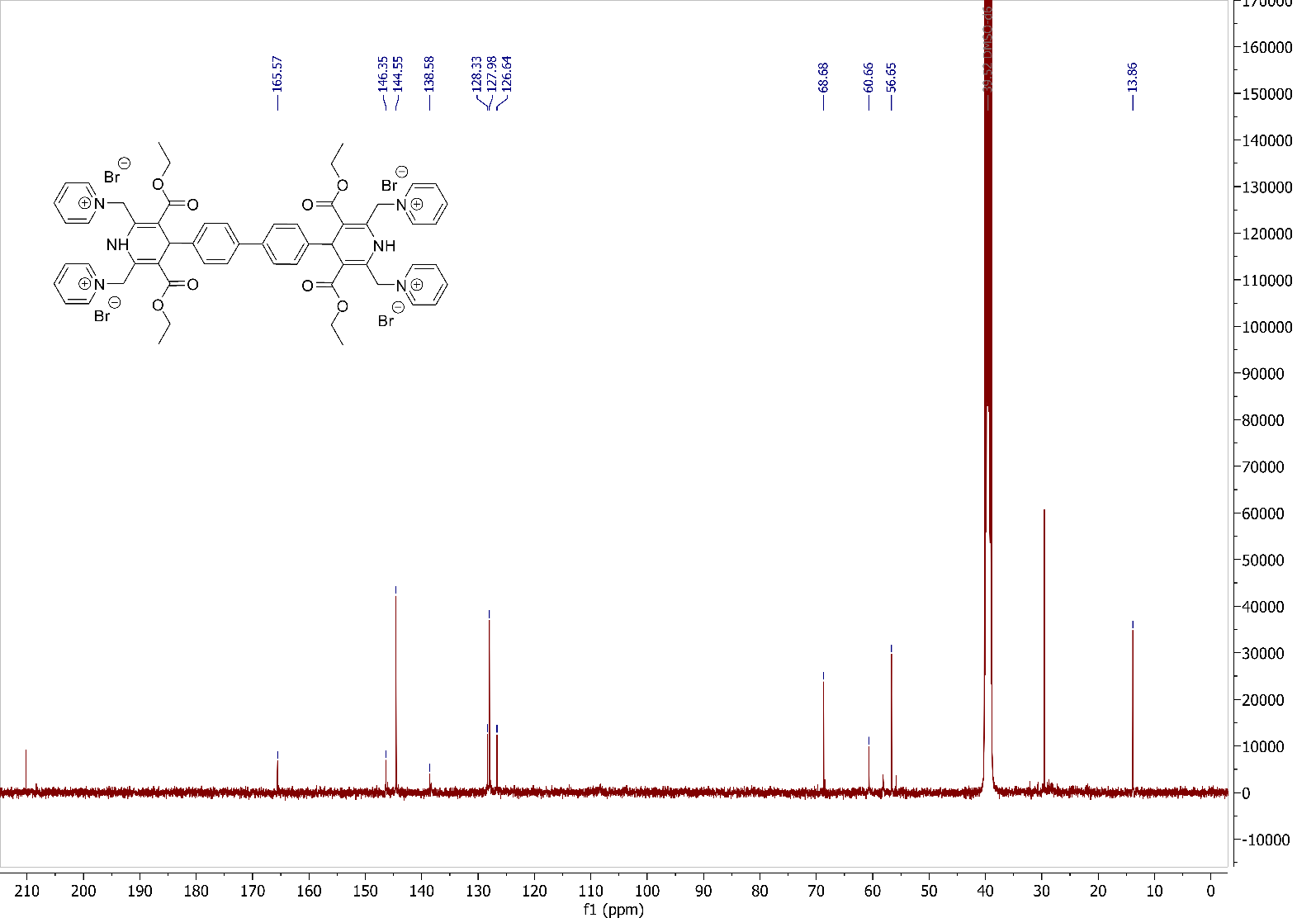


**Figure S9.** ^13^C NMR spectrum of diethyl 4-[4-[4-[3,5-*bis*(ethoxycarbonyl)-2,6-*bis*(pyridin-1-ium-1-ylmethyl)-1,4-dihydropyridin-4-yl]phenyl]phenyl]-2,6-*bis*(pyridin-1-ium-1-ylmethyl)-1,4-dihydropyridine-3,5-dicarboxylate tetrabromide (**22a**).


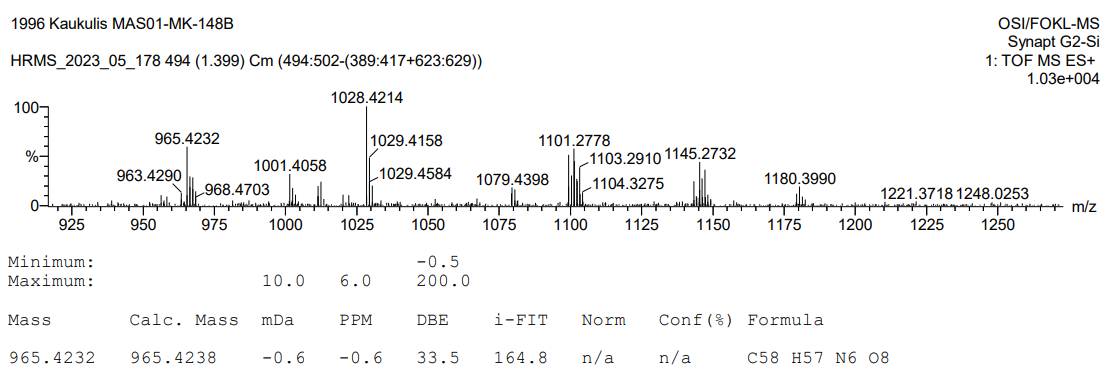


**Figure S10.** HRMS data of diethyl 4-[4-[4-[3,5-*bis*(ethoxycarbonyl)-2,6-*bis*(pyridin-1-ium-1-ylmethyl)-1,4-dihydropyridin-4-yl]phenyl]phenyl]-2,6-*bis*(pyridin-1-ium-1-ylmethyl)-1,4-dihydropyridine-3,5-dicarboxylate tetrabromide (**22a**).


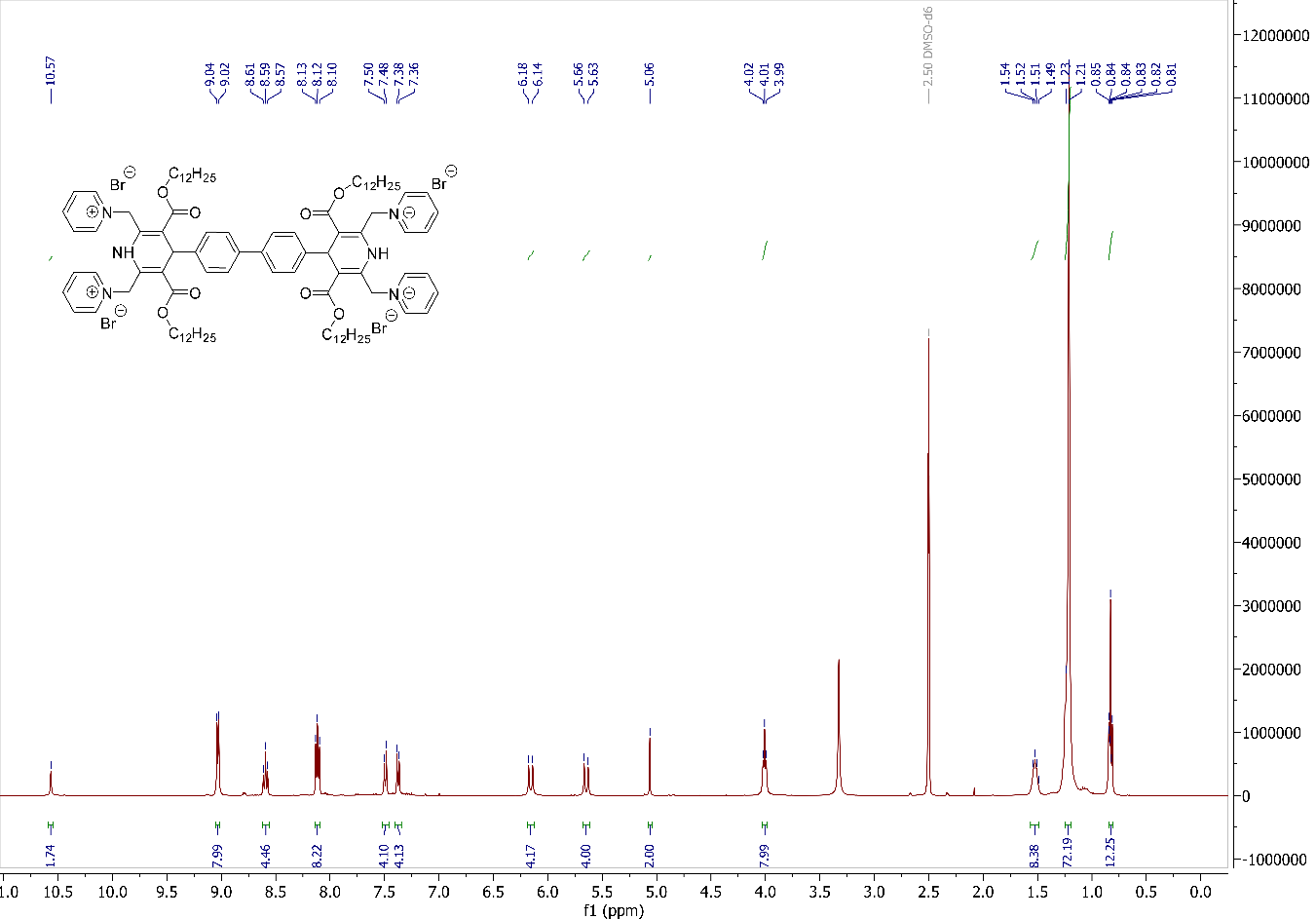


**Figure S11.** ^1^H NMR spectrum of didodecyl 4-[4-[4-[3,5-*bis*(dodecoxycarbonyl)-2,6-*bis*(pyridin-1-ium-1-ylmethyl)-1,4-dihydropyridin-4-yl]phenyl]phenyl]-2,6-*bis*(pyridin-1-ium-1-ylmethyl)-1,4-dihydropyridine-3,5-dicarboxylate tetrabromide (**22b**).


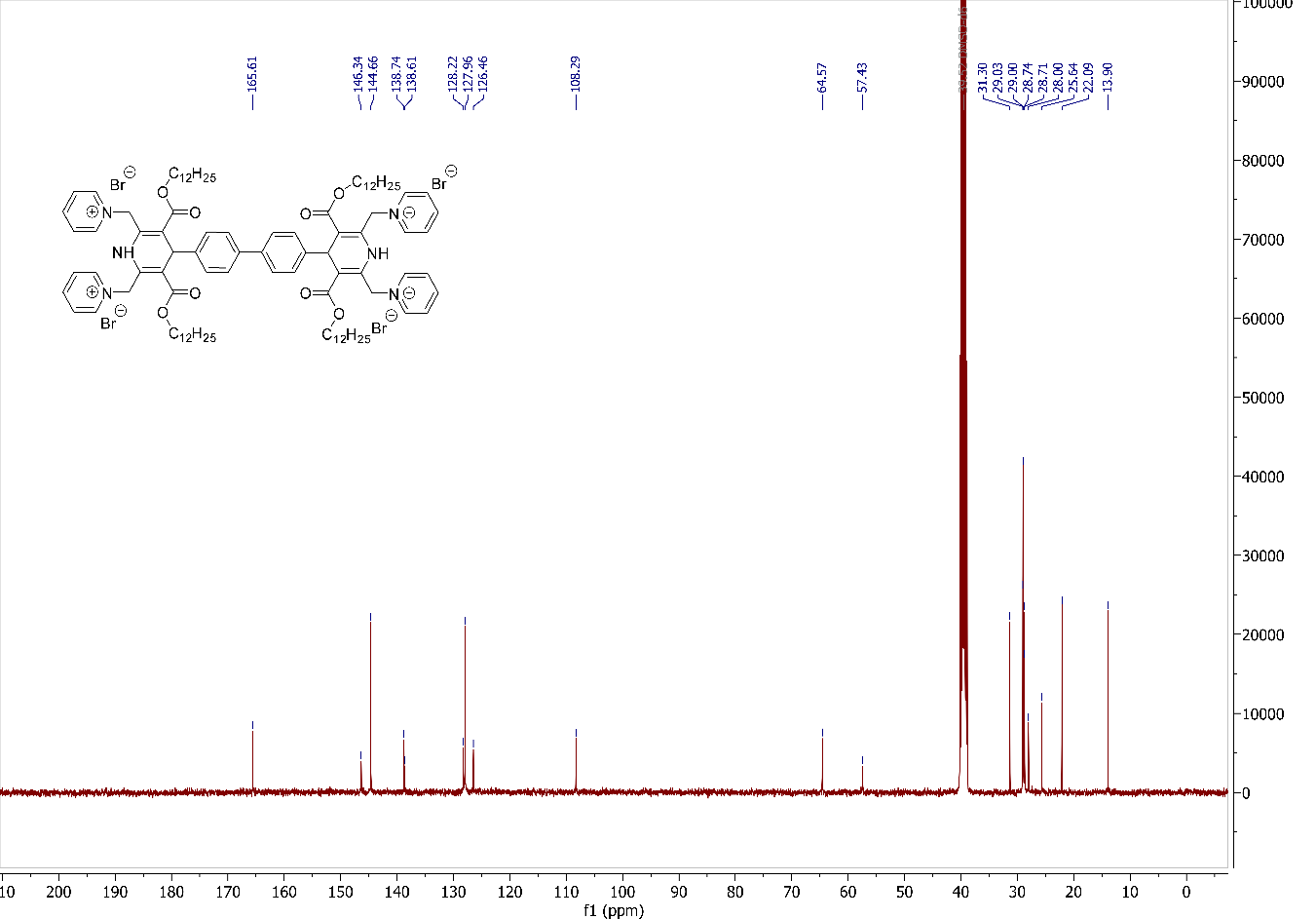


**Figure S12.** ^13^C NMR spectrum of didodecyl 4-[4-[4-[3,5-*bis*(dodecoxycarbonyl)-2,6-*bis*(pyridin-1-ium-1-ylmethyl)-1,4-dihydropyridin-4-yl]phenyl]phenyl]-2,6-*bis*(pyridin-1-ium-1-ylmethyl)-1,4-dihydropyridine-3,5-dicarboxylate tetrabromide (**22b**).


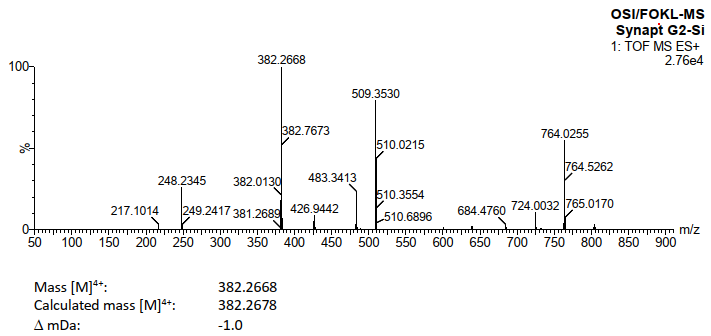


**Figure S13.** HRMS data of didodecyl 4-[4-[4-[3,5-*bis*(dodecoxycarbonyl)-2,6-*bis*(pyridin-1-ium-1-ylmethyl)-1,4-dihydropyridin-4-yl]phenyl]phenyl]-2,6-*bis*(pyridin-1-ium-1-ylmethyl)-1,4-dihydropyridine-3,5-dicarboxylate tetrabromide (**22b**).


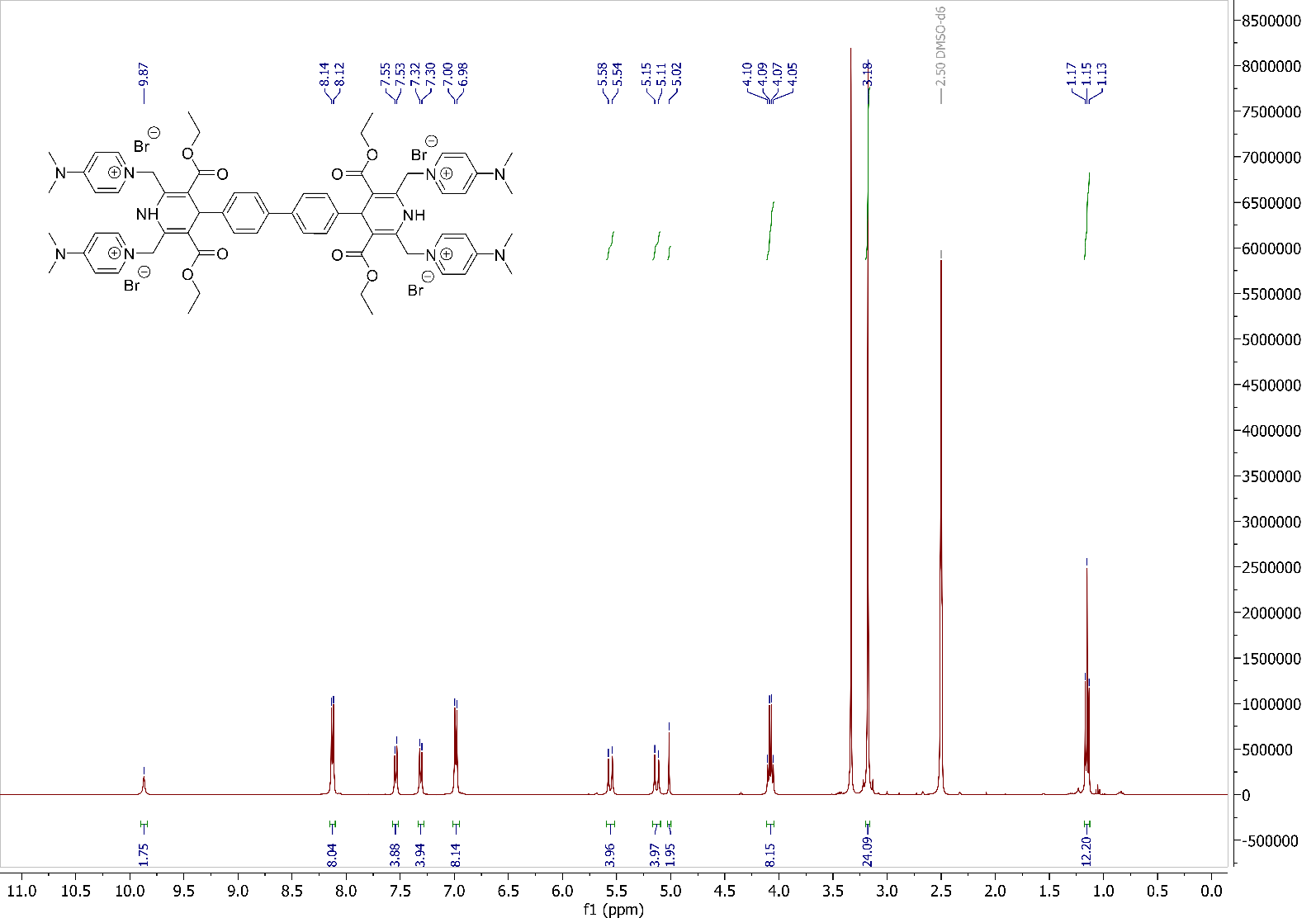


**Figure S14.** ^1^H NMR spectrum of diethyl 4-[4-[4-[2,6-*bis*[[4-(dimethylamino)pyridin-1-ium-1-yl]methyl]-3,5-*bis*(ethoxycarbonyl)-1,4-dihydropyridin-4-yl]phenyl]phenyl]-2,6-*bis*[[4-(dimethyl-amino)pyridin-1-ium-1-yl]methyl]-1,4-dihydropyridine-3,5-dicarboxylate tetrabromide (**23a**).


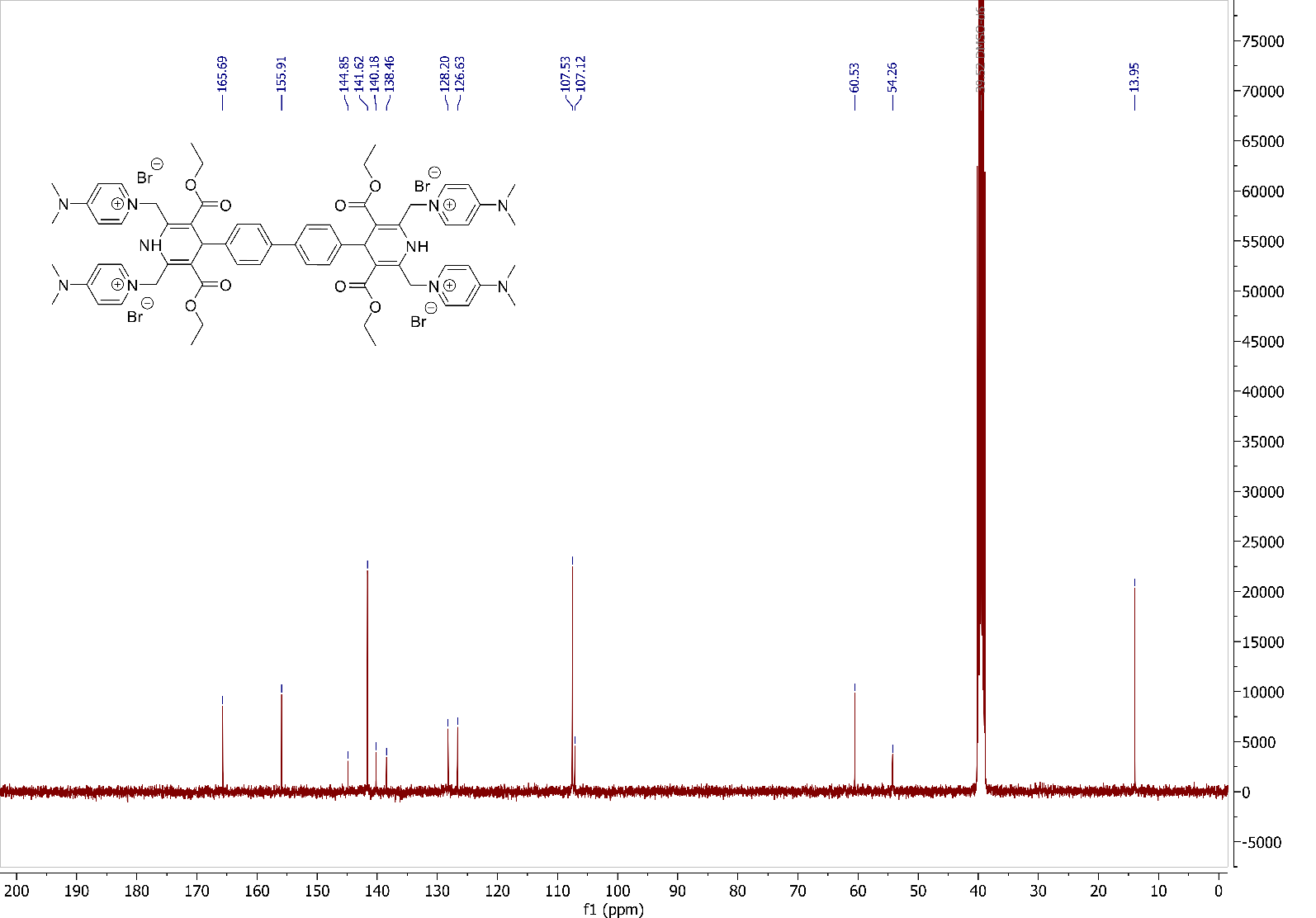


**Figure S15.** ^13^C NMR spectrum of diethyl 4-[4-[4-[2,6-*bis*[[4-(dimethylamino)pyridin-1-ium-1-yl]methyl]-3,5-*bis*(ethoxycarbonyl)-1,4-dihydropyridin-4-yl]phenyl]phenyl]-2,6-*bis*[[4-(dimethyl-amino)pyridin-1-ium-1-yl]methyl]-1,4-dihydropyridine-3,5-dicarboxylate tetrabromide (**23a**).


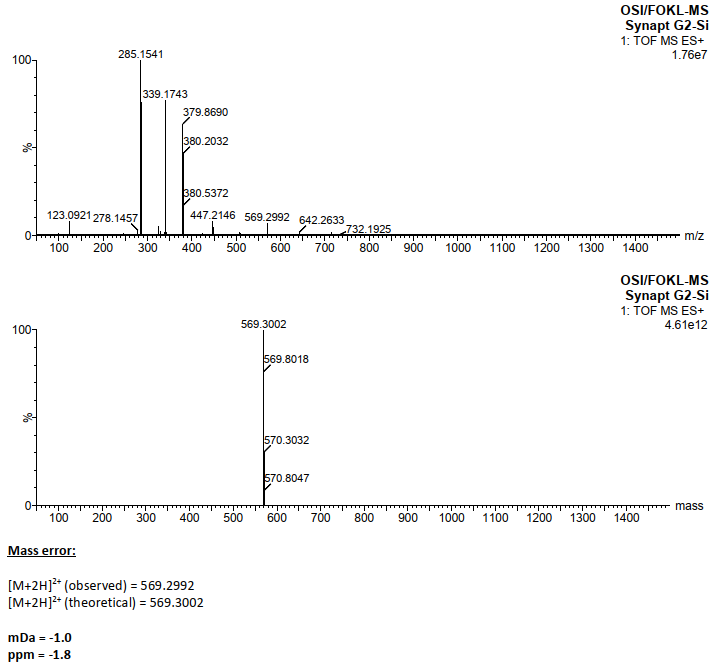


**Figure S16.** HRMS data of diethyl 4-[4-[4-[2,6-*bis*[[4-(dimethylamino)pyridin-1-ium-1-yl]methyl]-3,5-*bis*(ethoxycarbonyl)-1,4-dihydropyridin-4-yl]phenyl]phenyl]-2,6-*bis*[[4-(dimethylamino)pyridin-1-ium-1-yl]methyl]-1,4-dihydropyridine-3,5-dicarboxylate tetrabromide (**23a**).


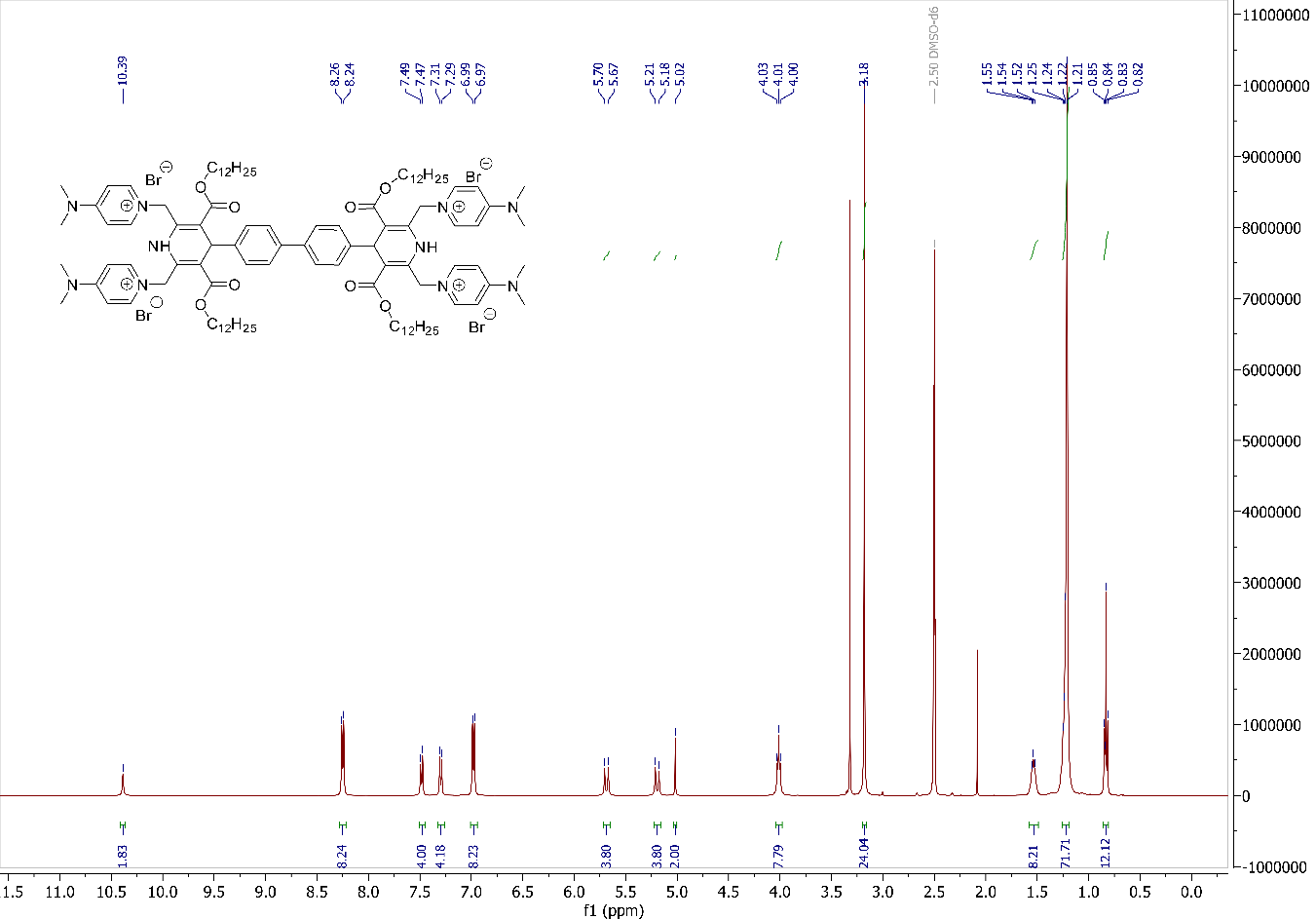


**Figure S17.** ^1^H NMR spectrum of didodecyl 4-[4-[4-[2,6-*bis*[[4-(dimethylamino)pyridin-1-ium-1-yl]methyl]-3,5-*bis*(dodecoxycarbonyl)-1,4-dihydropyridin-4-yl]phenyl]phenyl]-2,6-*bis*[[4-(dimethyl-amino)pyridin-1-ium-1-yl]methyl]-1,4-dihydropyridine-3,5-dicarboxylate tetrabromide (**23b**).


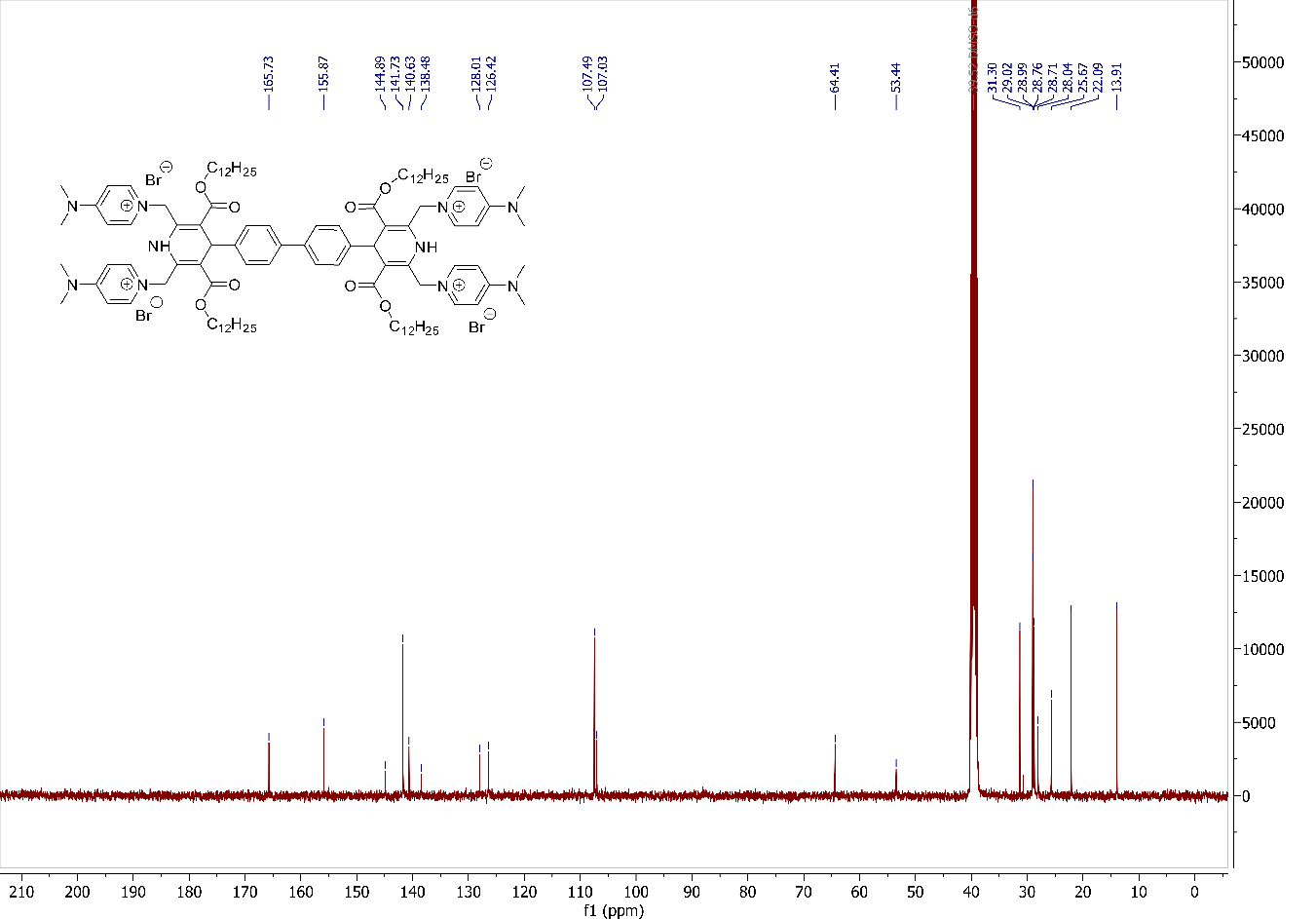


**Figure S18.** ^13^C NMR spectrum of didodecyl 4-[4-[4-[2,6-*bis*[[4-(dimethylamino)pyridin-1-ium-1-yl]methyl]-3,5-*bis*(dodecoxycarbonyl)-1,4-dihydropyridin-4-yl]phenyl]phenyl]-2,6-*bis*[[4-(dimethyl-amino)pyridin-1-ium-1-yl]methyl]-1,4-dihydropyridine-3,5-dicarboxylate tetrabromide (**23b**).


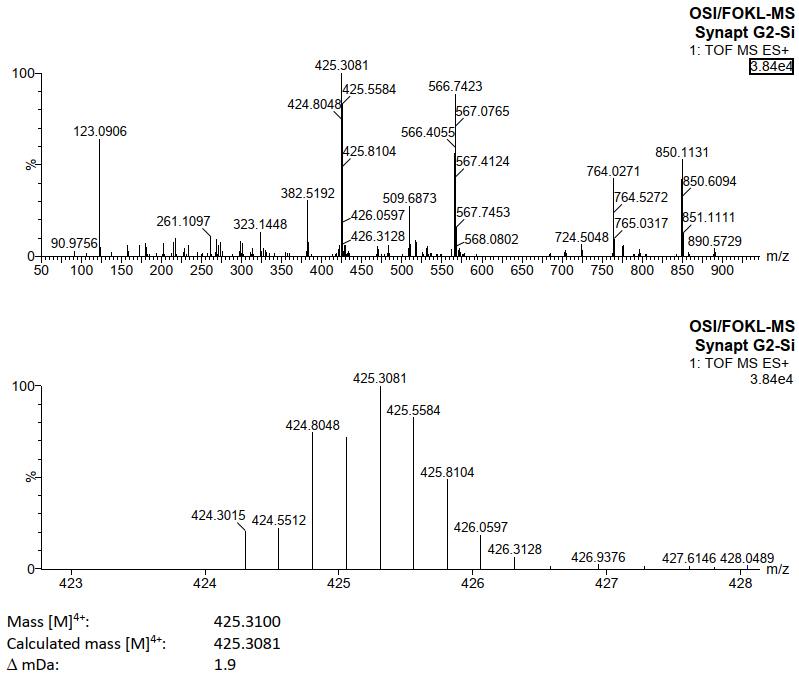


**Figure S19.** HRMS data of didodecyl 4-[4-[4-[2,6-*bis*[[4-(dimethylamino)pyridin-1-ium-1-yl]methyl]-3,5-*bis*(dodecoxycarbonyl)-1,4-dihydropyridin-4-yl]phenyl]phenyl]-2,6-*bis*[[4-(dimethylamino)pyridin-1-ium-1-yl]methyl]-1,4-dihydropyridine-3,5-dicarboxylate tetrabromide (**23b**).


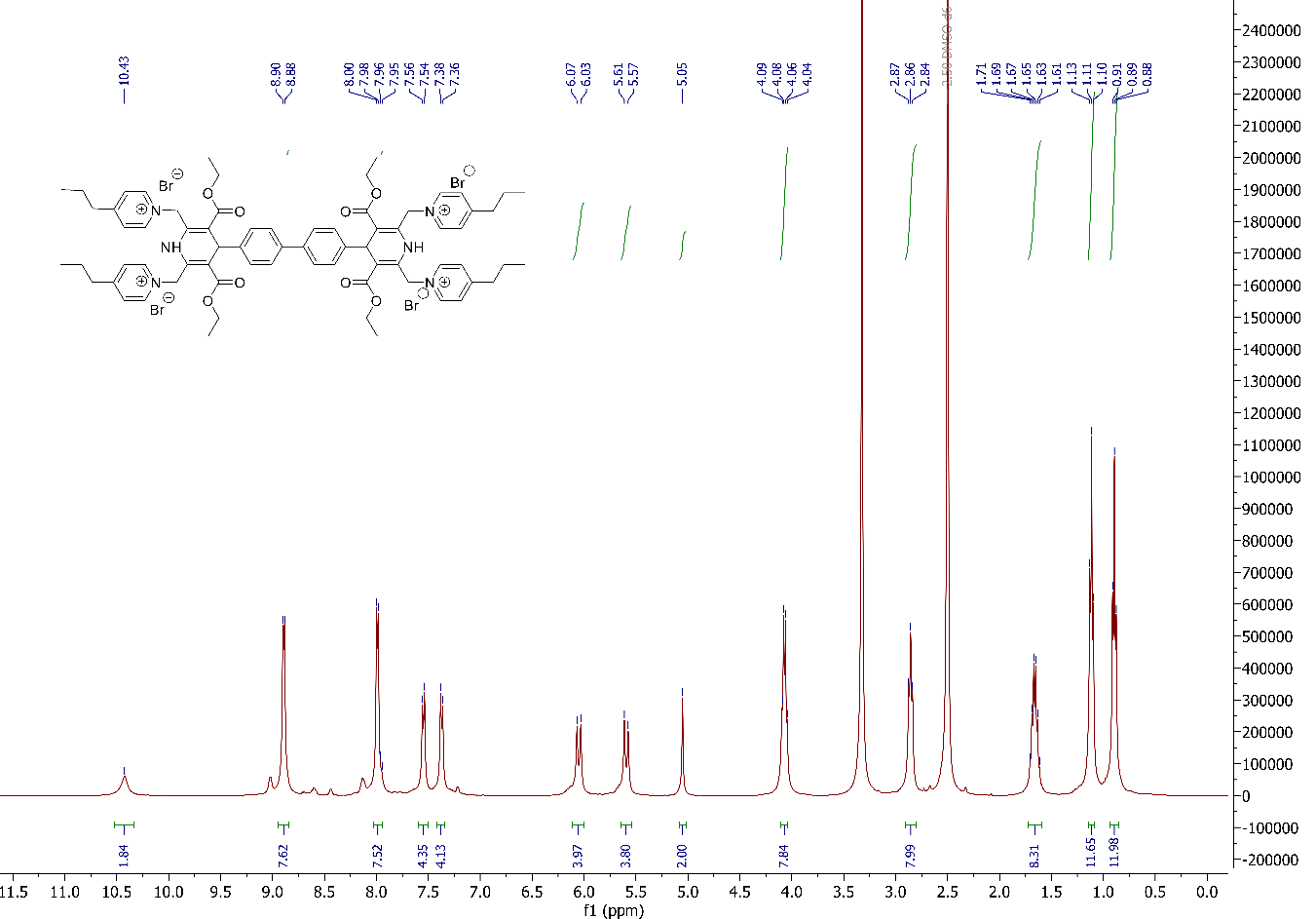


**Figure S20.** ^1^H NMR spectrum of diethyl 4-[4-[4-[3,5-*bis*(ethoxycarbonyl)-2,6-*bis*[(4-propylpyridin-1-ium-1-yl)methyl]-1,4-dihydropyridin-4-yl]phenyl]phenyl]-2,6-*bis*[(4-propylpyridin-1-ium-1-yl)methyl]-1,4-dihydro-pyridine-3,5-dicarboxylate tetrabromide (**24a**).


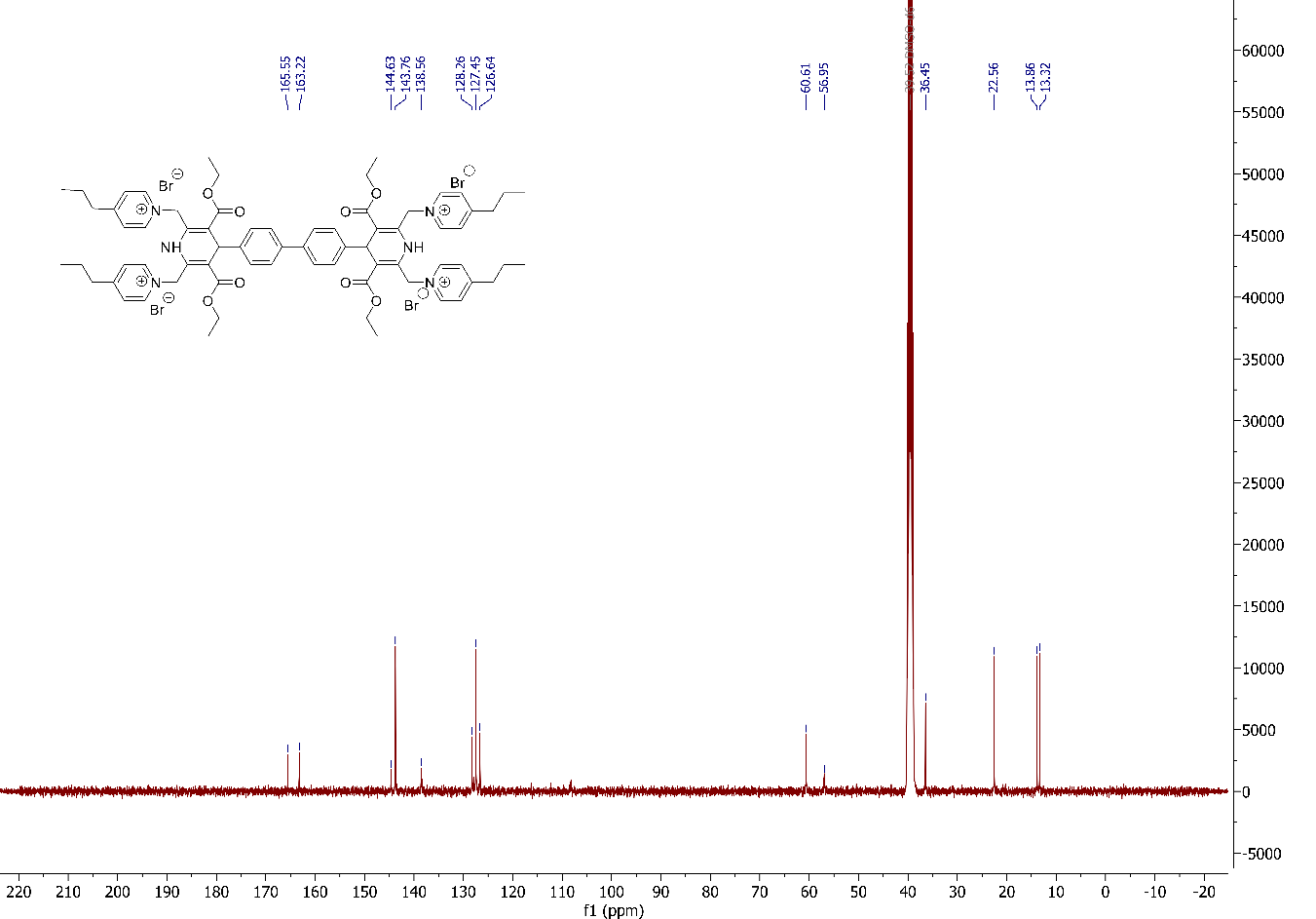


**Figure S21.** ^13^C NMR spectrum of diethyl 4-[4-[4-[3,5-*bis*(ethoxycarbonyl)-2,6-*bis*[(4-propylpyridin-1-ium-1-yl)methyl]-1,4-dihydropyridin-4-yl]phenyl]phenyl]-2,6-*bis*[(4-propylpyridin-1-ium-1-yl)methyl]-1,4-dihydro-pyridine-3,5-dicarboxylate tetrabromide (**24a**).


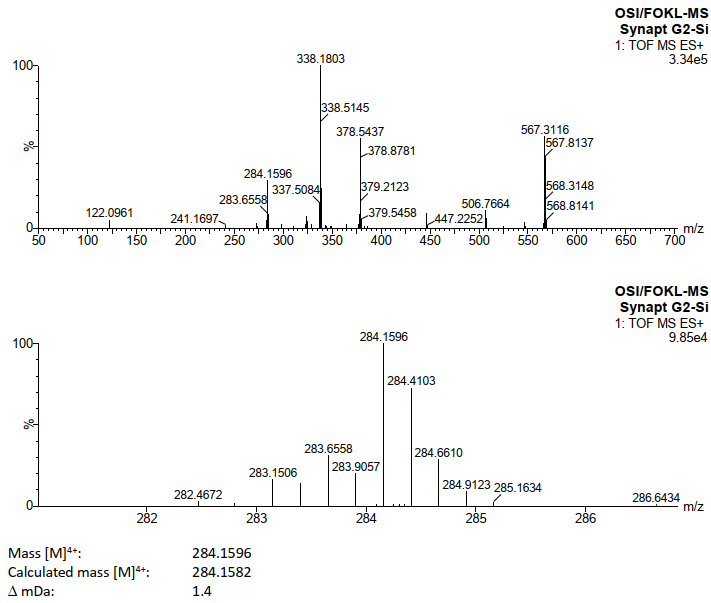


**Figure S22.** HRMS data of diethyl 4-[4-[4-[3,5-*bis*(ethoxycarbonyl)-2,6-*bis*[(4-propylpyridin-1-ium-1-yl)methyl]-1,4-dihydropyridin-4-yl]phenyl]phenyl]-2,6-*bis*[(4-propylpyridin-1-ium-1-yl)methyl]-1,4-dihydro-pyridine-3,5-dicarboxylate tetrabromide (**24a**).


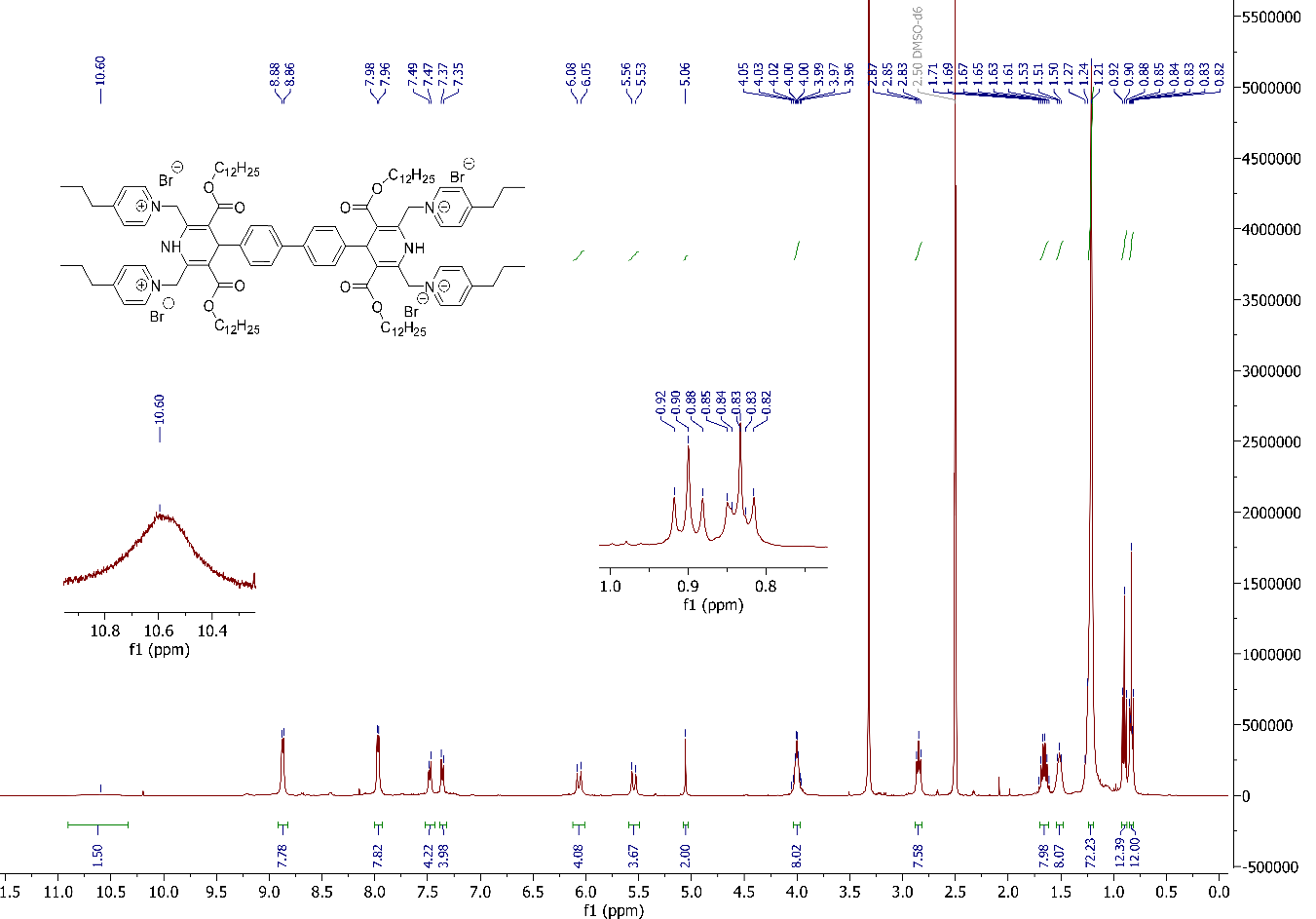


**Figure S23.** ^1^H NMR spectrum of didodecyl 4-[4-[4-[3,5-*bis*(dodecoxycarbonyl)-2,6-*bis*[(4-propylpyridin-1-ium-1-yl)methyl]-1,4-dihydropyridin-4-yl]phenyl]phenyl]-2,6-*bis*[(4-propylpyridin-1-ium-1-yl)methyl]-1,4-dihydro-pyridine-3,5-dicarboxylate tetrabromide (**24b**).


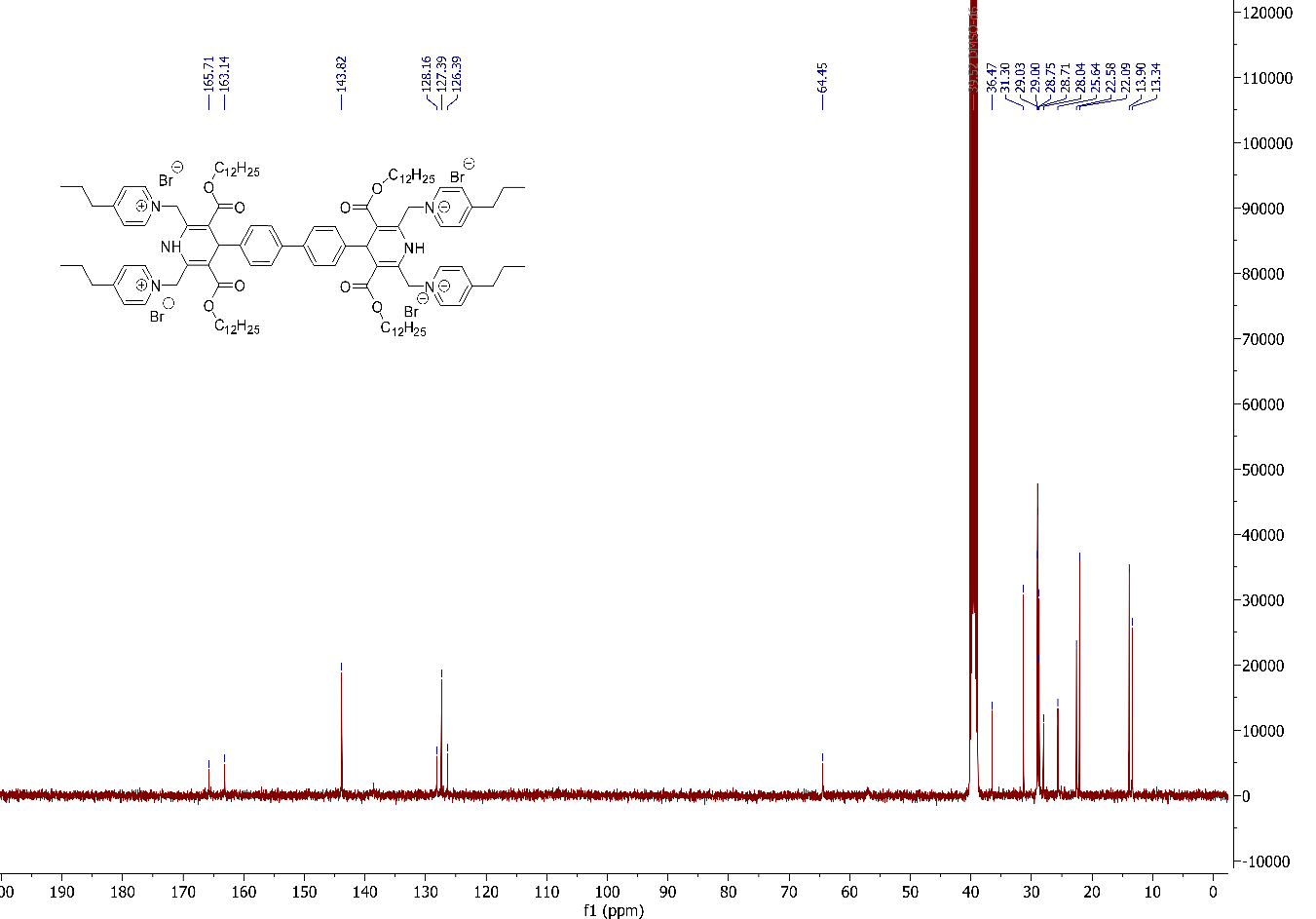


**Figure S24.** ^13^C NMR spectrum of didodecyl 4-[4-[4-[3,5-*bis*(dodecoxycarbonyl)-2,6-bis[(4-propylpyridin-1-ium-1-yl)methyl]-1,4-dihydropyridin-4-yl]phenyl]phenyl]-2,6-*bis*[(4-propylpyridin-1-ium-1-yl)methyl]-1,4-dihydro-pyridine-3,5-dicarboxylate tetrabromide (**24b**).


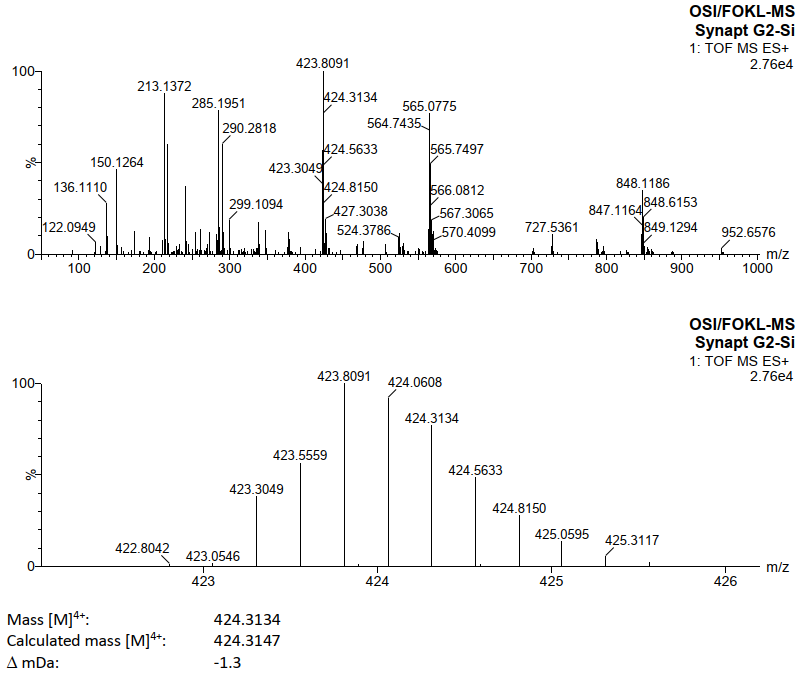


**Figure S25.** HRMS data of didodecyl 4-[4-[4-[3,5-*bis*(dodecoxycarbonyl)-2,6-*bis*[(4-propylpyridin-1-ium-1-yl)methyl]-1,4-dihydropyridin-4-yl]phenyl]phenyl]-2,6-*bis*[(4-propylpyridin-1-ium-1-yl)methyl]-1,4-dihydro-pyridine-3,5-dicarboxylate tetrabromide (**24b**).


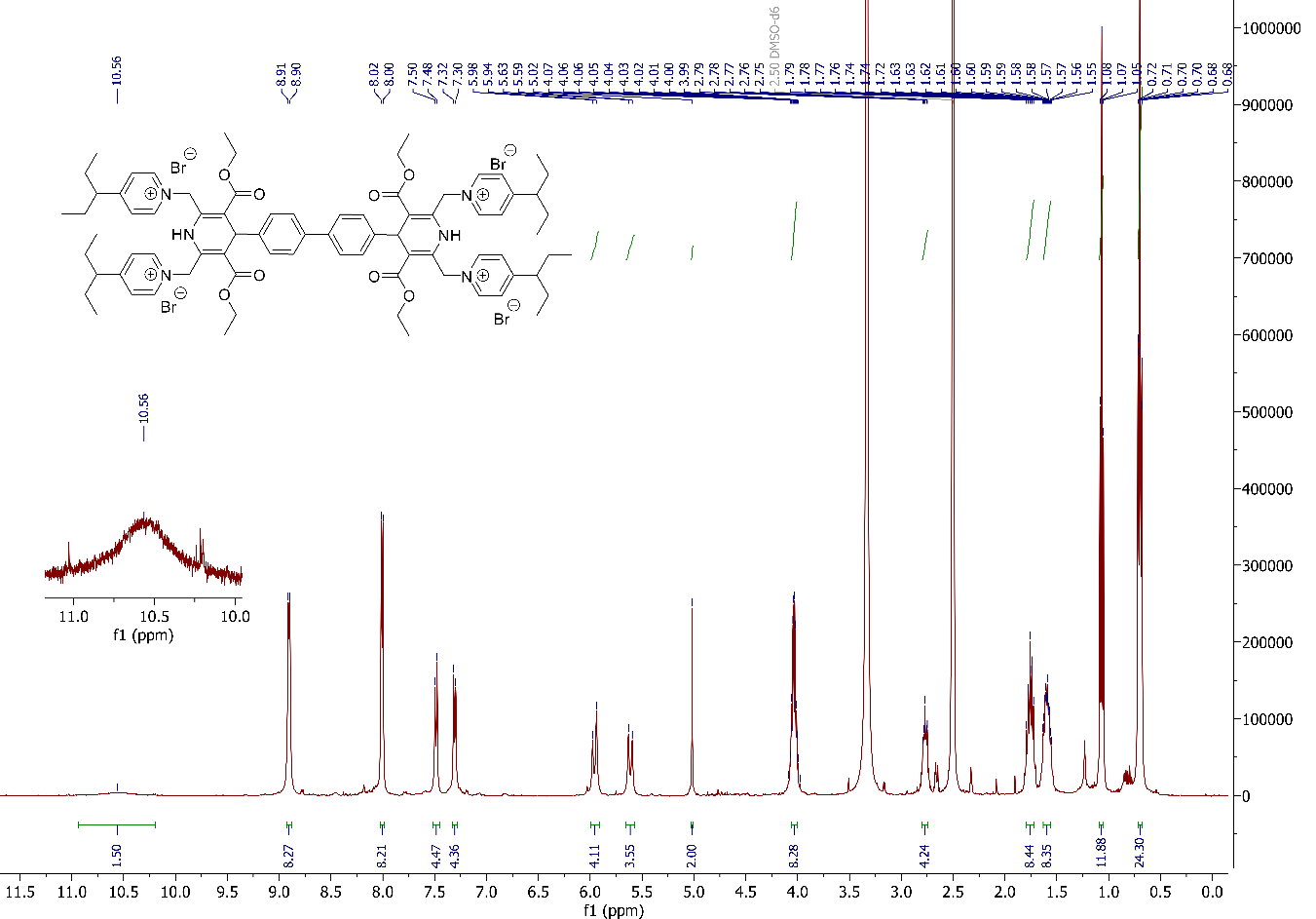


**Figure S26.** ^1^H NMR spectrum of diethyl 4-[4-[4-[3,5-*bis*(ethoxycarbonyl)-2,6-*bis*[[4-(1-ethylpropyl)pyridin-1-ium-1-yl]-methyl]-1,4-dihydropyridin-4-yl]phenyl]phenyl]-2,6-*bis*[[4-(1-ethyl-propyl)pyridin-1-ium-1-yl]methyl]-1,4-dihydropyridine-3,5-dicarboxylate tetrabromide (**25a**).


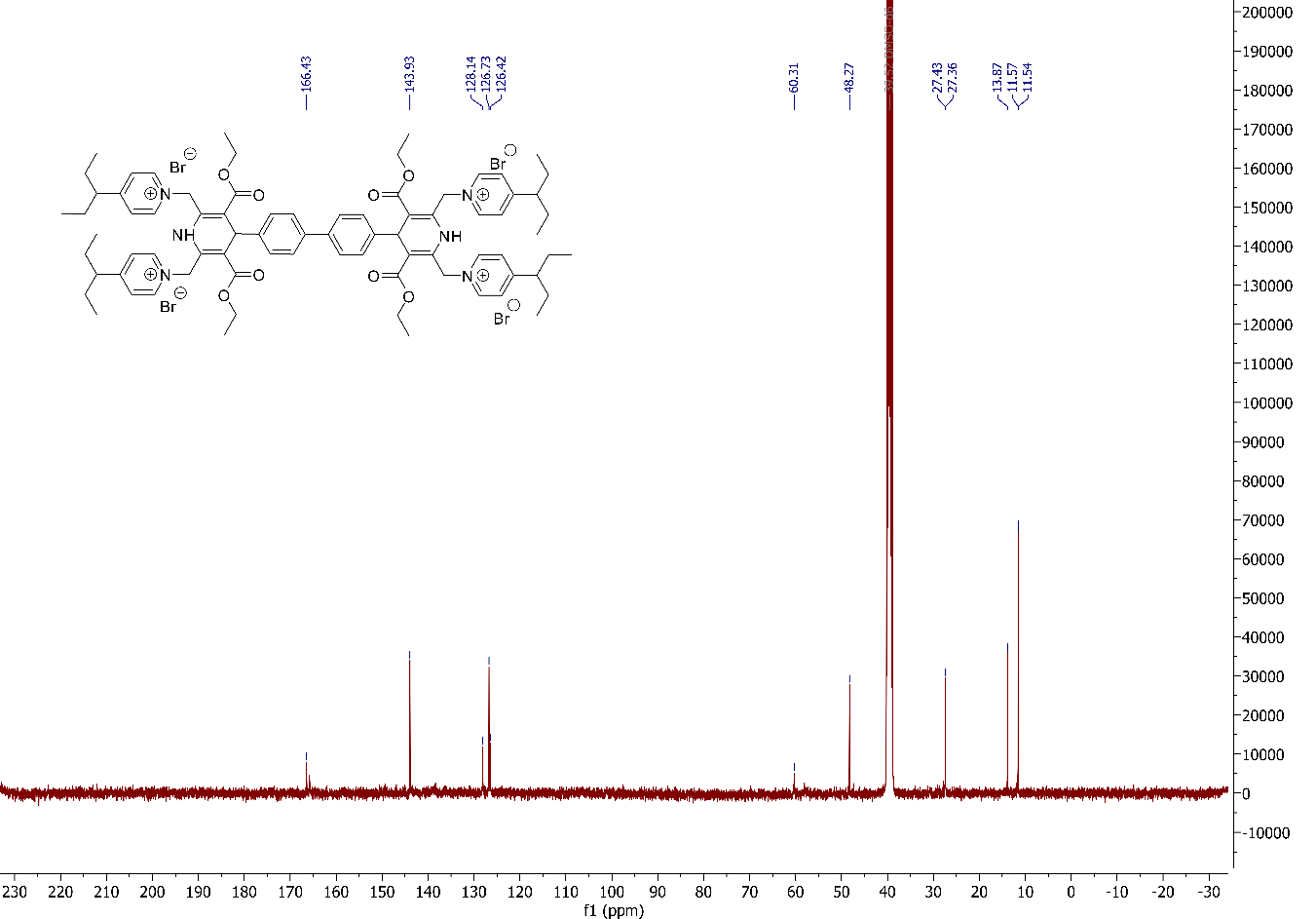


**Figure S27.** ^13^C NMR spectrum of diethyl 4-[4-[4-[3,5-*bis*(ethoxycarbonyl)-2,6-*bis*[[4-(1-ethylpropyl)pyridin-1-ium-1-yl]-methyl]-1,4-dihydropyridin-4-yl]phenyl]phenyl]-2,6-*bis*[[4-(1-ethyl-propyl)pyridin-1-ium-1-yl]methyl]-1,4-dihydropyridine-3,5-dicarboxylate tetrabromide (**25a**).


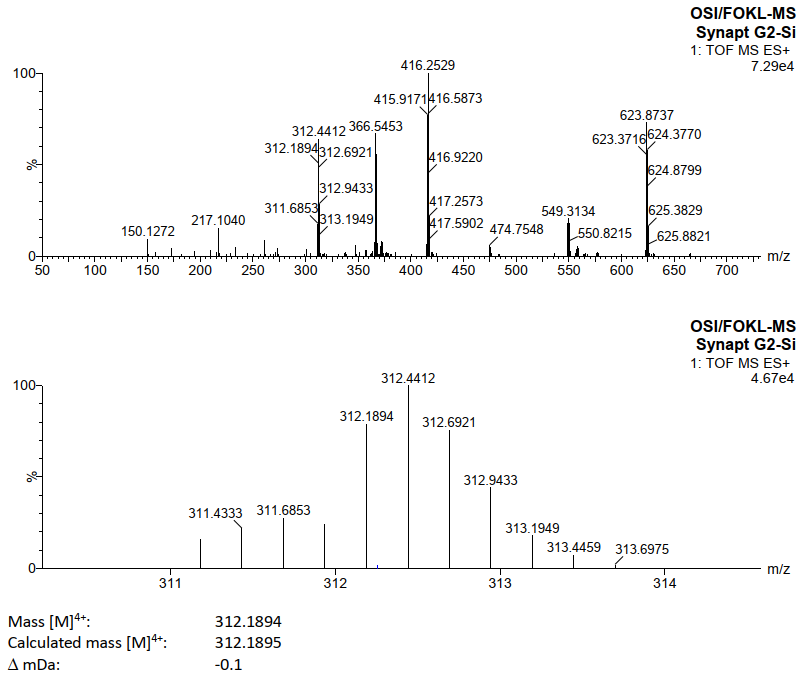


**Figure S28.** HRMS data of diethyl 4-[4-[4-[3,5-*bis*(ethoxycarbonyl)-2,6-*bis*[[4-(1-ethylpropyl)pyridin-1-ium-1-yl]-methyl]-1,4-dihydropyridin-4-yl]phenyl]phenyl]-2,6-*bis*[[4-(1-ethyl-propyl)pyridin-1-ium-1-yl]methyl]-1,4-dihydropyridine-3,5-dicarboxylate tetrabromide (**25a**).


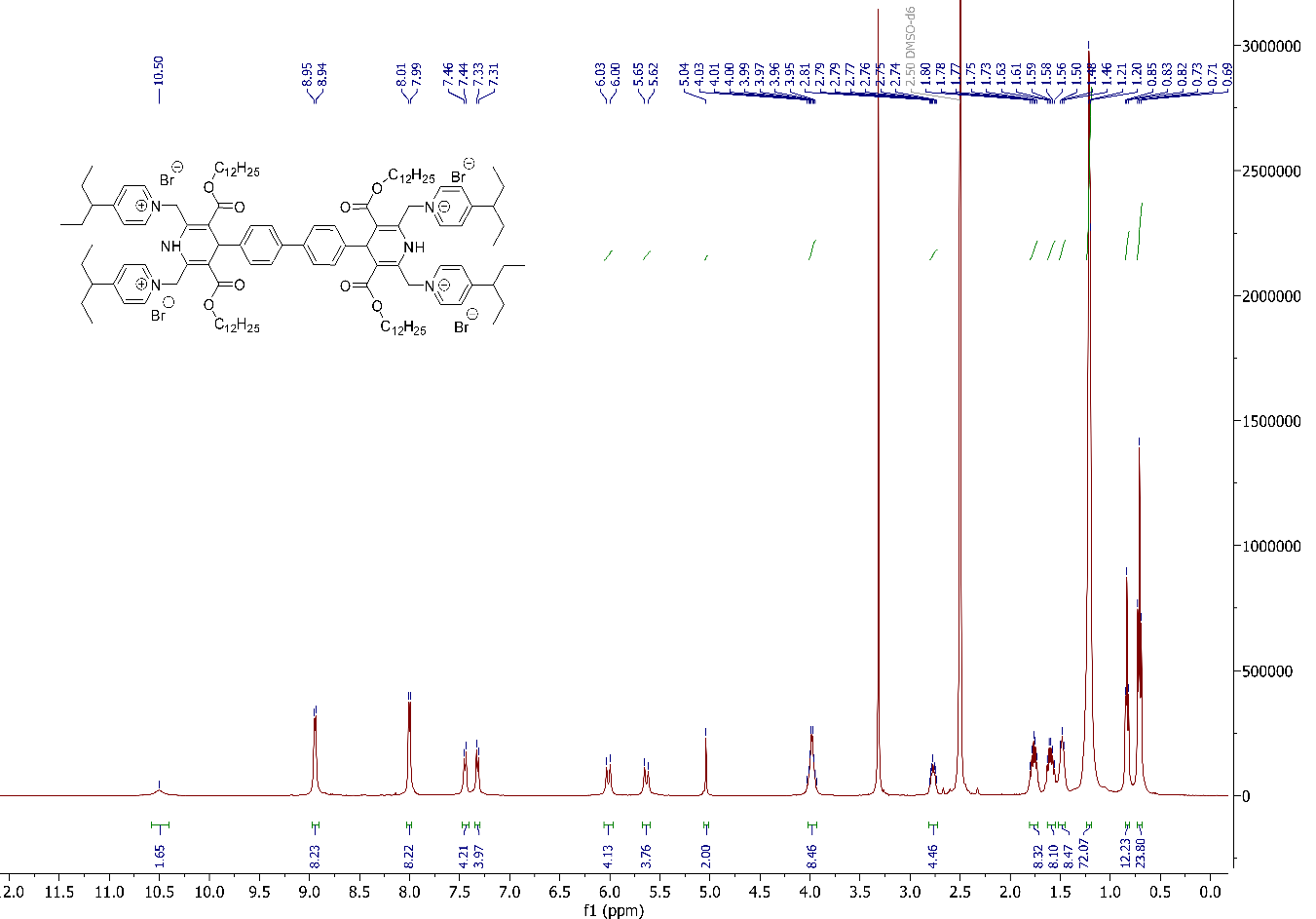


**Figure S29.** ^1^H NMR spectrum of didodecyl 4-[4-[4-[3,5-*bis*(dodecoxycarbonyl)-2,6-*bis*[[4-(1-ethylpropyl)pyridin-1-ium-1-yl]-methyl]-1,4-dihydropyridin-4-yl]phenyl]phenyl]-2,6-*bis*[[4-(1-ethyl-propyl)pyridin-1-ium-1-yl]methyl]-1,4-dihydropyridine-3,5-dicarboxylate (**25b**).


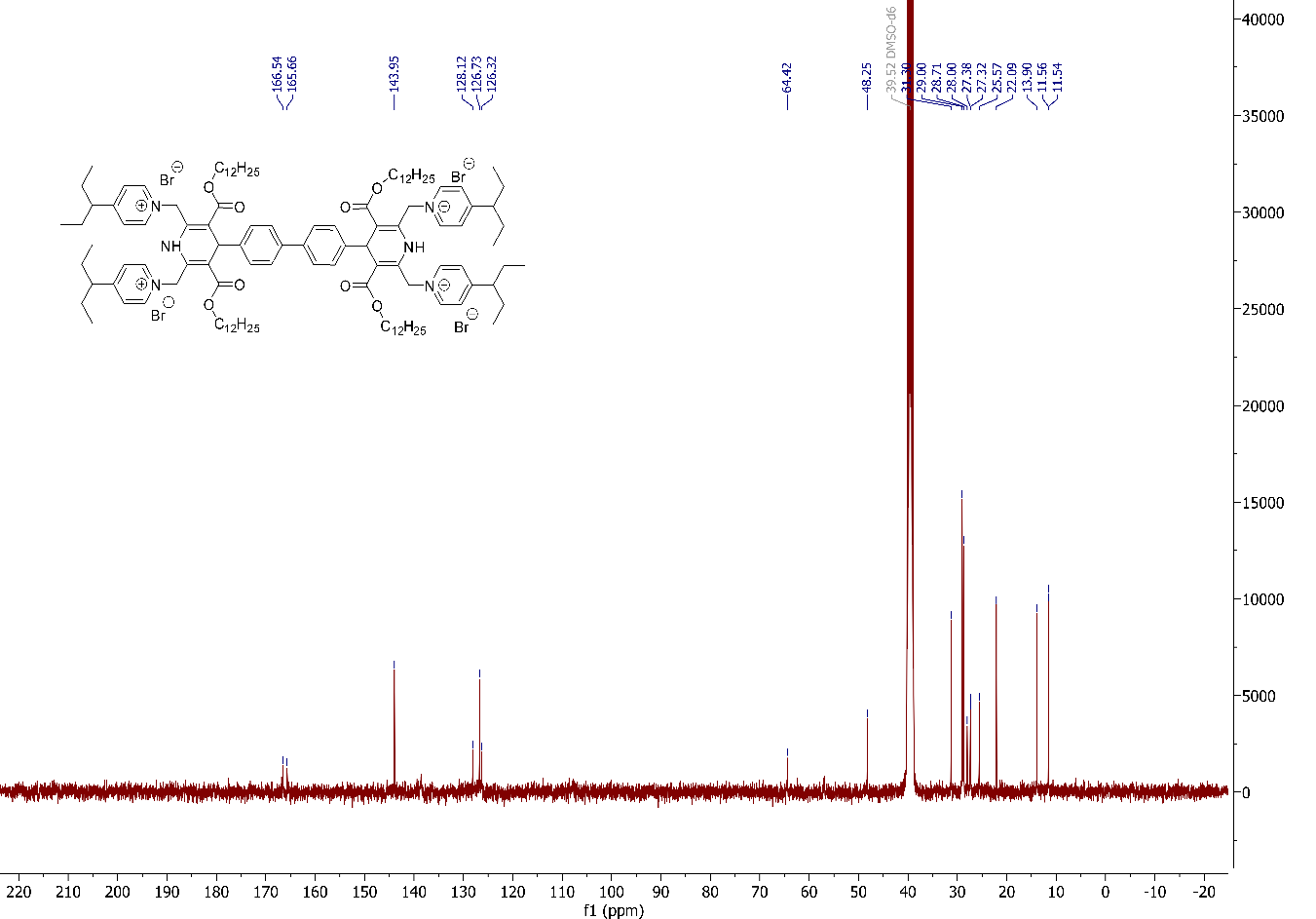


**Figure S30.** ^13^C NMR spectrum of didodecyl 4-[4-[4-[3,5-*bis*(dodecoxycarbonyl)-2,6-*bis*[[4-(1-ethylpropyl)pyridin-1-ium-1-yl]-methyl]-1,4-dihydropyridin-4-yl]phenyl]phenyl]-2,6-*bis*[[4-(1-ethyl-propyl)pyridin-1-ium-1-yl]methyl]-1,4-dihydropyridine-3,5-dicarboxylate (**25b**).


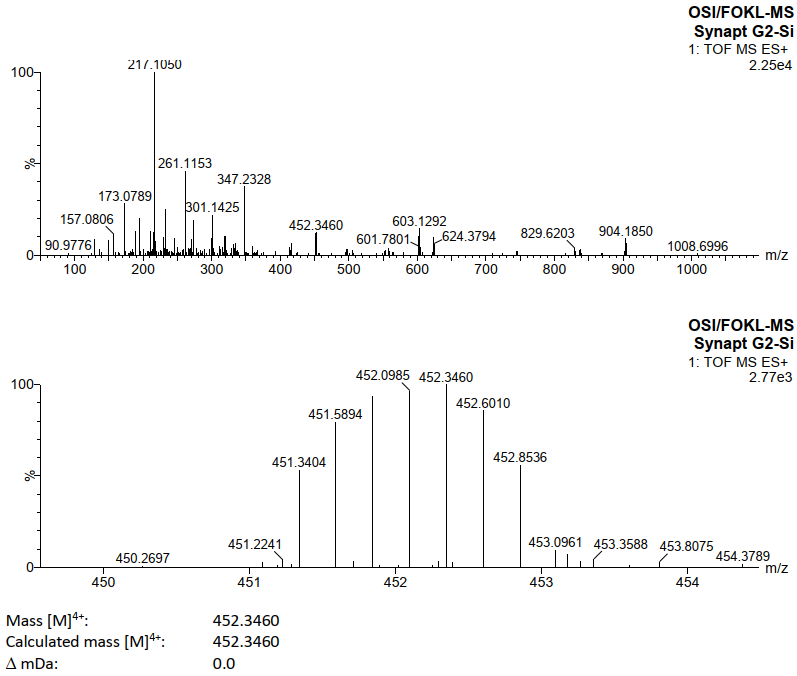


**Figure S31.** HRMS data of didodecyl 4-[4-[4-[3,5-*bis*(dodecoxycarbonyl)-2,6-*bis*[[4-(1-ethylpropyl)pyridin-1-ium-1-yl]-methyl]-1,4-dihydropyridin-4-yl]phenyl]phenyl]-2,6-*bis*[[4-(1-ethyl-propyl)pyridin-1-ium-1-yl]methyl]-1,4-dihydropyridine-3,5-dicarboxylate (**25b**).


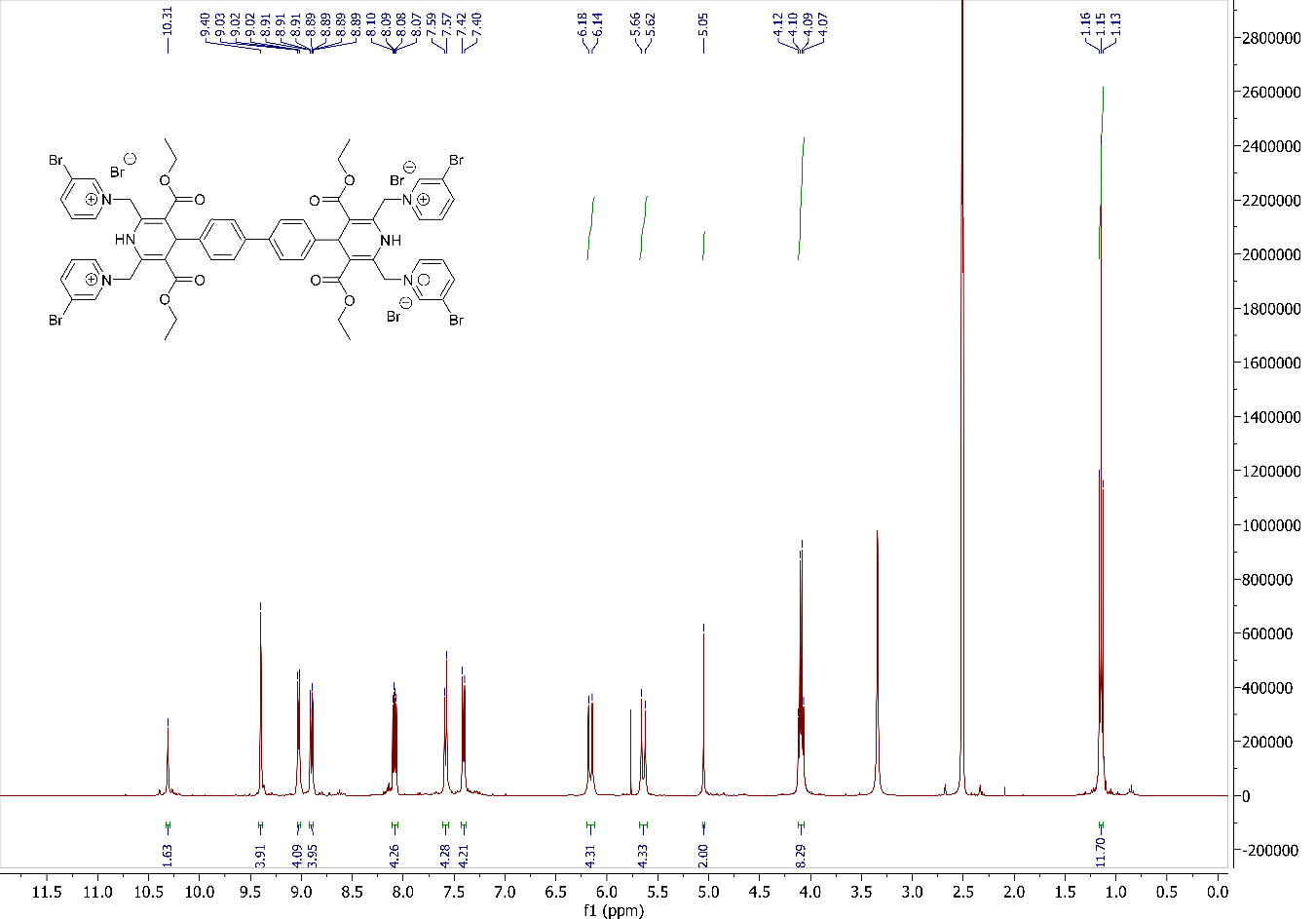


**Figure S32.** ^1^H NMR spectrum of diethyl 4-[4-[4-[2,6-*bis*[(3-bromopyridin-1-ium-1-yl)methyl]-3,5-*bis*(ethoxycarbonyl)-1,4-dihydropyridin-4-yl]phenyl]phenyl]-2,6-*bis*[(3-bromopyridin-1-ium-1-yl)methyl]-1,4-dihydro-pyridine-3,5-dicarboxylate tetrabromide (**26a**).


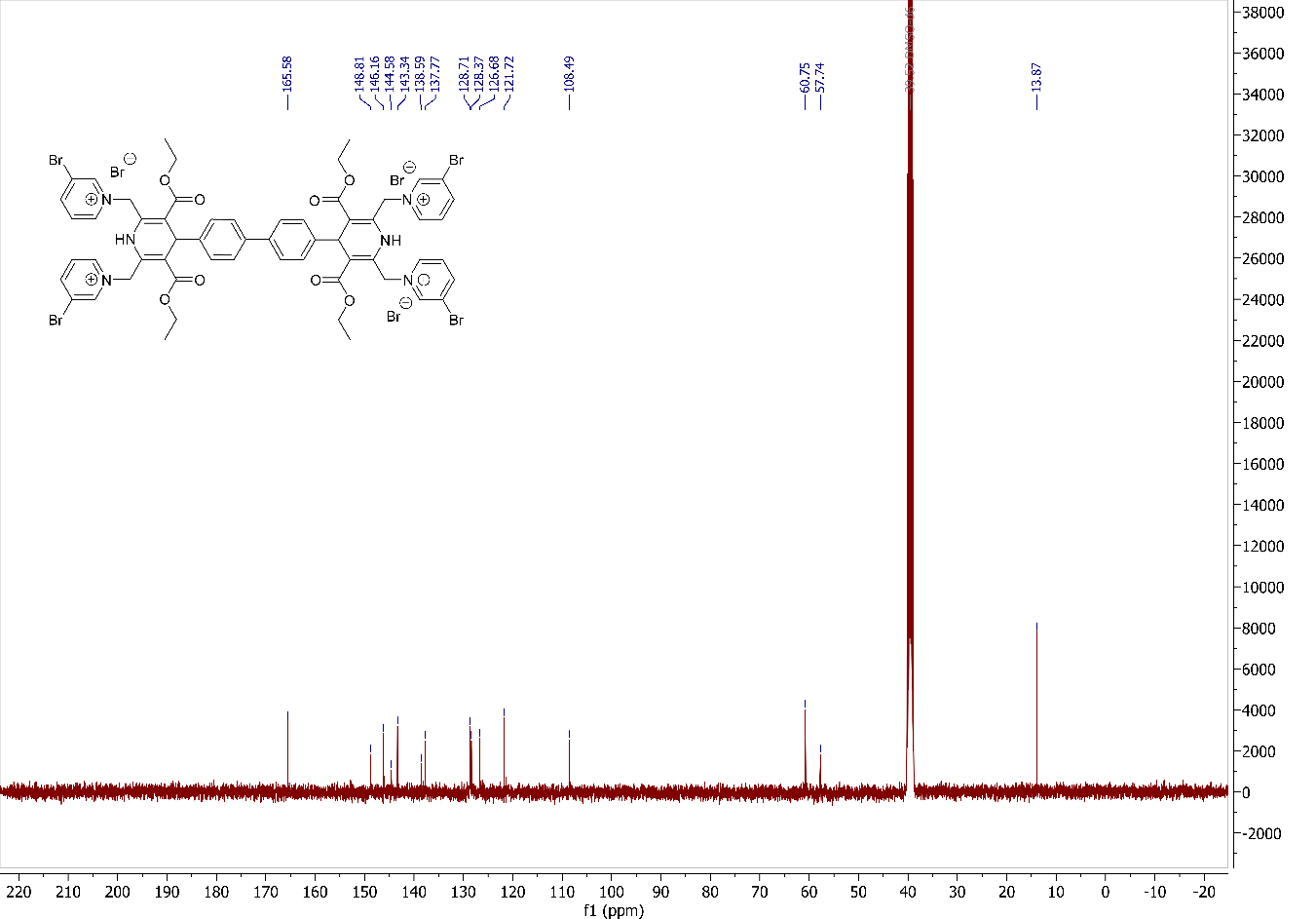


**Figure S33.** ^13^C NMR spectrum of diethyl 4-[4-[4-[2,6-*bis*[(3-bromopyridin-1-ium-1-yl)methyl]-3,5-*bis*(ethoxycarbonyl)-1,4-dihydropyridin-4-yl]phenyl]phenyl]-2,6-*bis*[(3-bromopyridin-1-ium-1-yl)methyl]-1,4-dihydro-pyridine-3,5-dicarboxylate tetrabromide (**26a**).


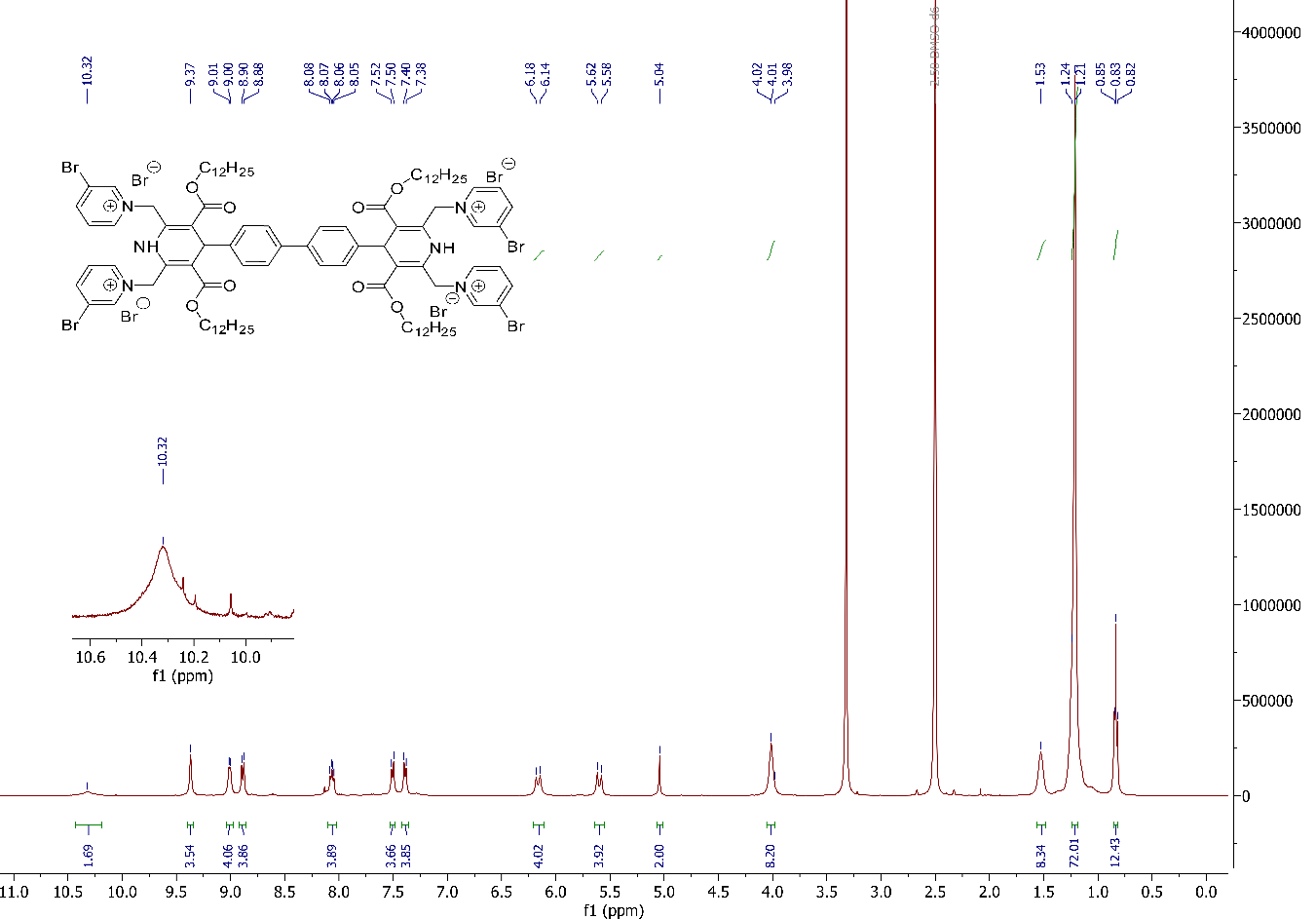


**Figure S34.** ^1^H NMR spectrum of didodecyl 4-[4-[4-[2,6-*bis*[(3-bromopyridin-1-ium-1-yl)methyl]-3,5-*bis*(dodecoxycarbonyl)-1,4-dihydropyridin-4-yl]phenyl]phenyl]-2,6-*bis*[(3-bromopyridin-1-ium-1-yl)methyl]-1,4-dihydro-pyridine-3,5-dicarboxylate tetrabromide (**26b**).


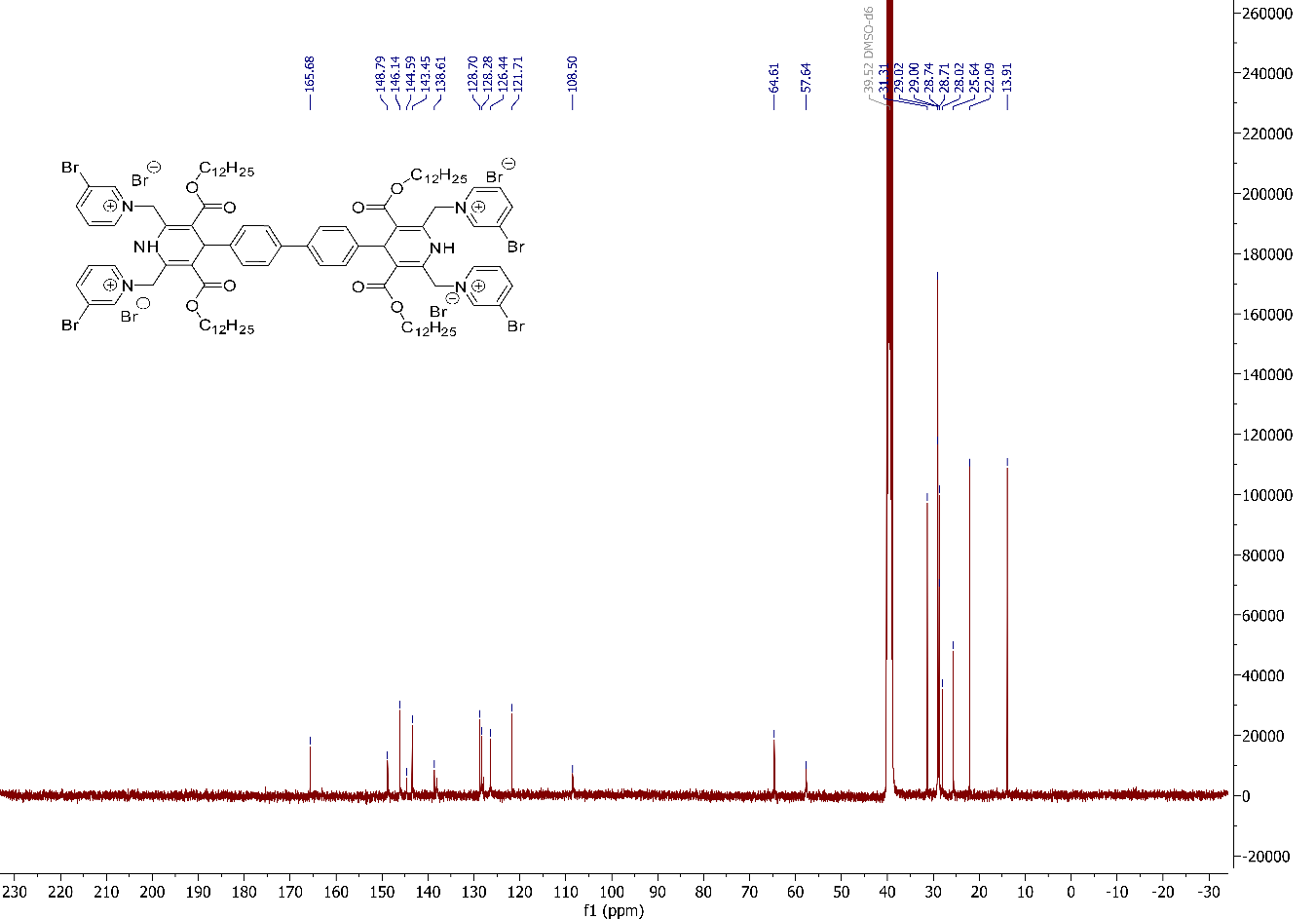


**Figure S35.** ^13^C NMR spectrum of didodecyl 4-[4-[4-[2,6-*bis*[(3-bromopyridin-1-ium-1-yl)methyl]-3,5-*bis*(dodecoxycarbonyl)-1,4-dihydropyridin-4-yl]phenyl]phenyl]-2,6-*bis*[(3-bromopyridin-1-ium-1-yl)methyl]-1,4-dihydro-pyridine-3,5-dicarboxylate tetrabromide (**26b**).


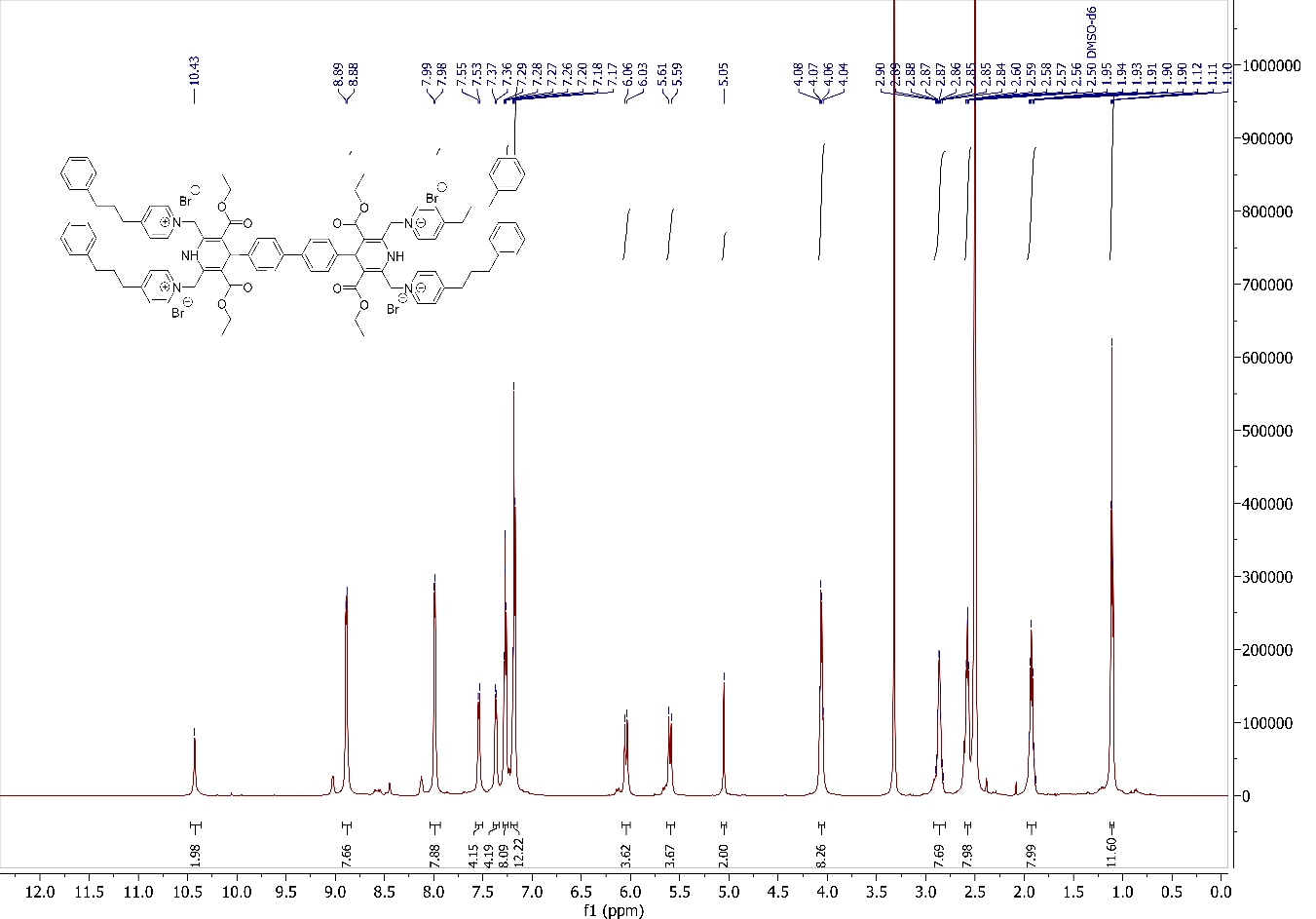


**Figure S36.** ^1^H NMR spectrum of diethyl 4-[4-[4-[3,5-bis(ethoxycarbonyl)-2,6-bis[[4-(3-phenylpropyl)pyridin-1-ium-1-yl]methyl]-1,4-dihydropyridin-4-yl]phenyl]phenyl]-2,6-bis[[4-(3-phenyl-propyl)pyridin-1-ium-1-yl]methyl]-1,4-dihydropyridine-3,5-dicarboxylate tetrabromide (**27a**).


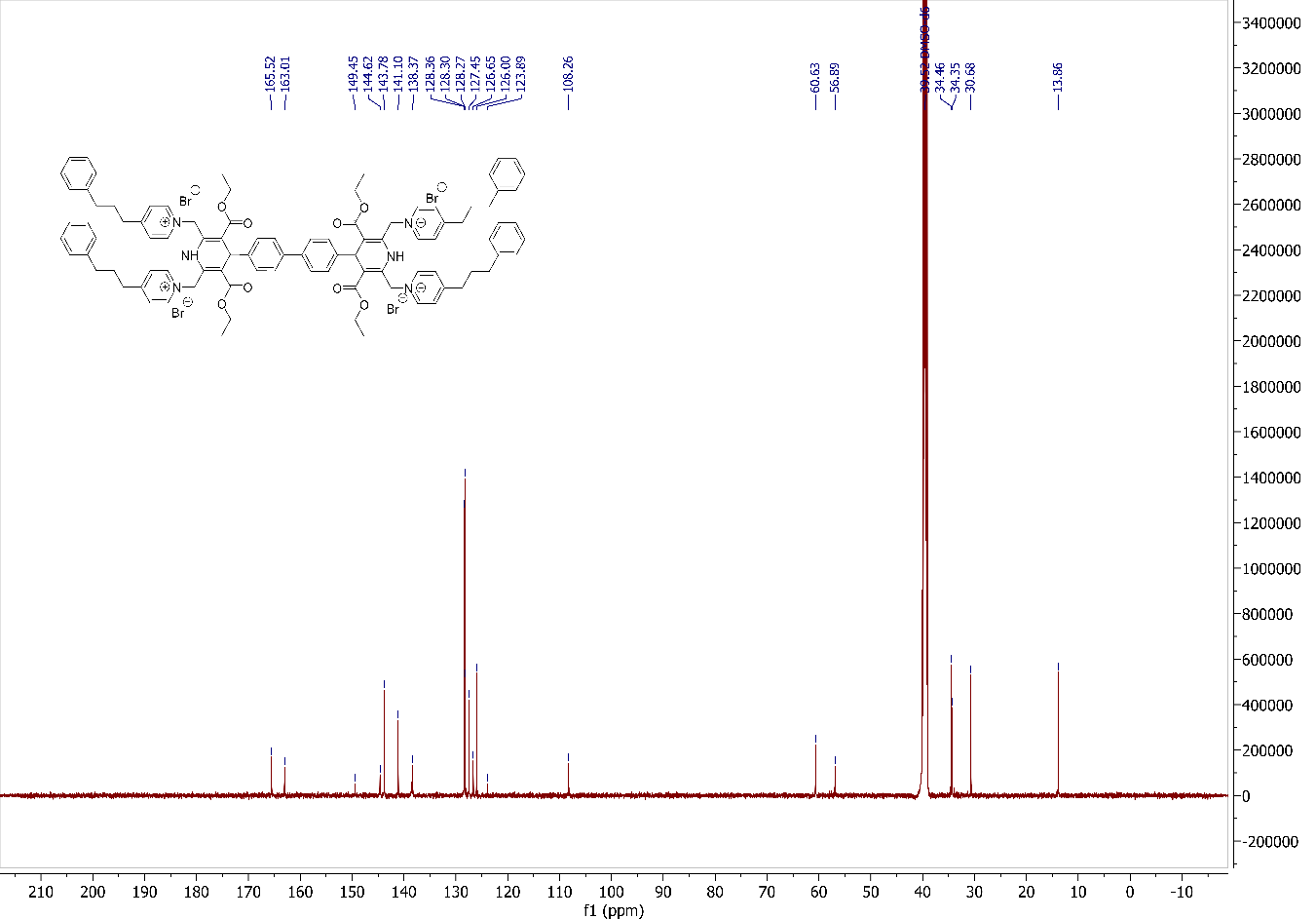


**Figure S37.** ^13^C NMR spectrum of diethyl 4-[4-[4-[3,5-bis(ethoxycarbonyl)-2,6-bis[[4-(3-phenylpropyl)pyridin-1-ium-1-yl]methyl]-1,4-dihydropyridin-4-yl]phenyl]phenyl]-2,6-bis[[4-(3-phenyl-propyl)pyridin-1-ium-1-yl]methyl]-1,4-dihydropyridine-3,5-dicarboxylate tetrabromide (**27a**).


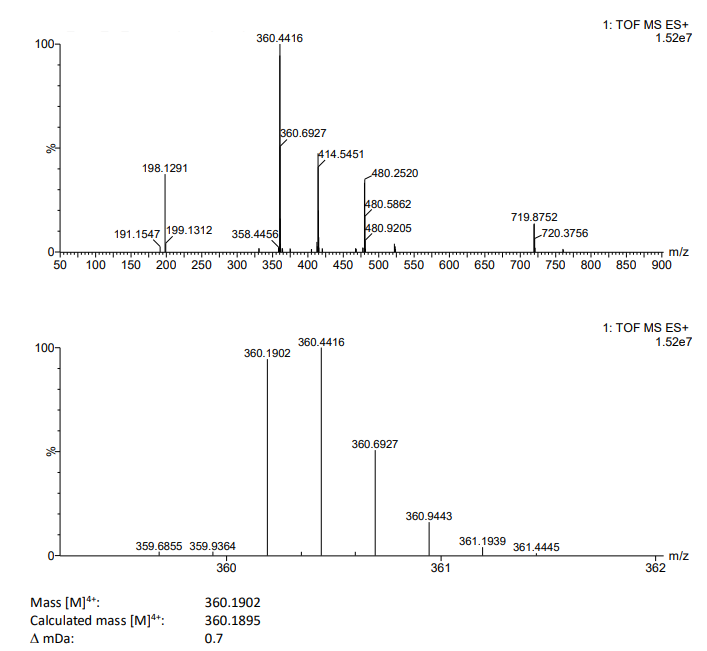


**Figure S38.** HRMS data of diethyl 4-[4-[4-[3,5-bis(ethoxycarbonyl)-2,6-bis[[4-(3-phenylpropyl)pyridin-1-ium-1-yl]methyl]-1,4-dihydropyridin-4-yl]phenyl]phenyl]-2,6-bis[[4-(3-phenylpropyl)pyridin-1-ium-1-yl]methyl]-1,4-dihydropyridine-3,5-dicarboxylate tetrabromide (**27a**).


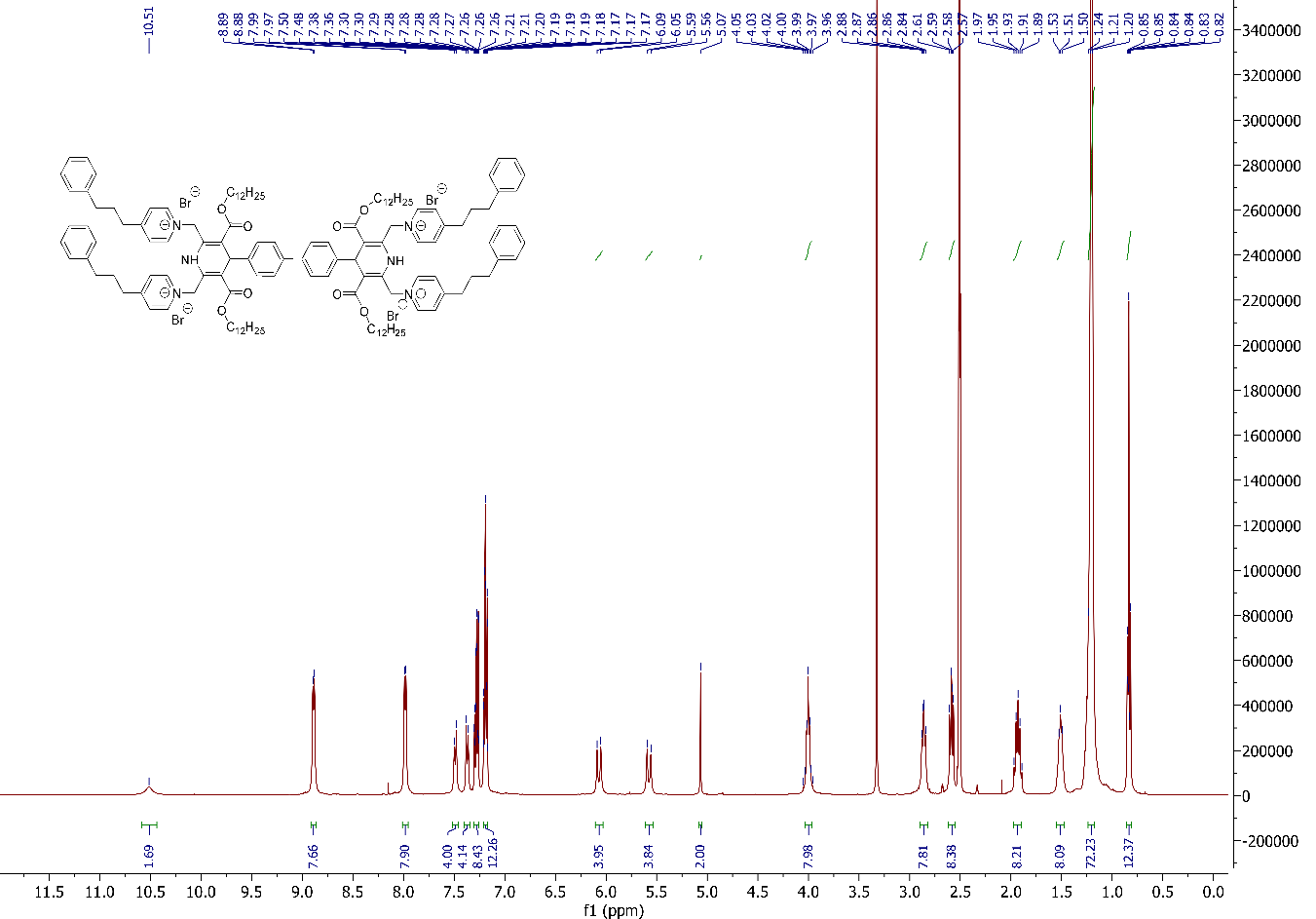


**Figure S39.** ^1^H NMR spectrum of didodecyl 4-[4-[4-[3,5-*bis*(dodecoxycarbonyl)-2,6-*bis*[[4-(3-phenylpropyl)pyridin-1-ium-1-yl]methyl]-1,4-dihydropyridin-4-yl]phenyl]phenyl]-2,6-*bis*[[4-(3-phenyl-propyl)pyridin-1-ium-1-yl]methyl]-1,4-dihydropyridine-3,5-dicarboxylate tetrabromide (**27b**).


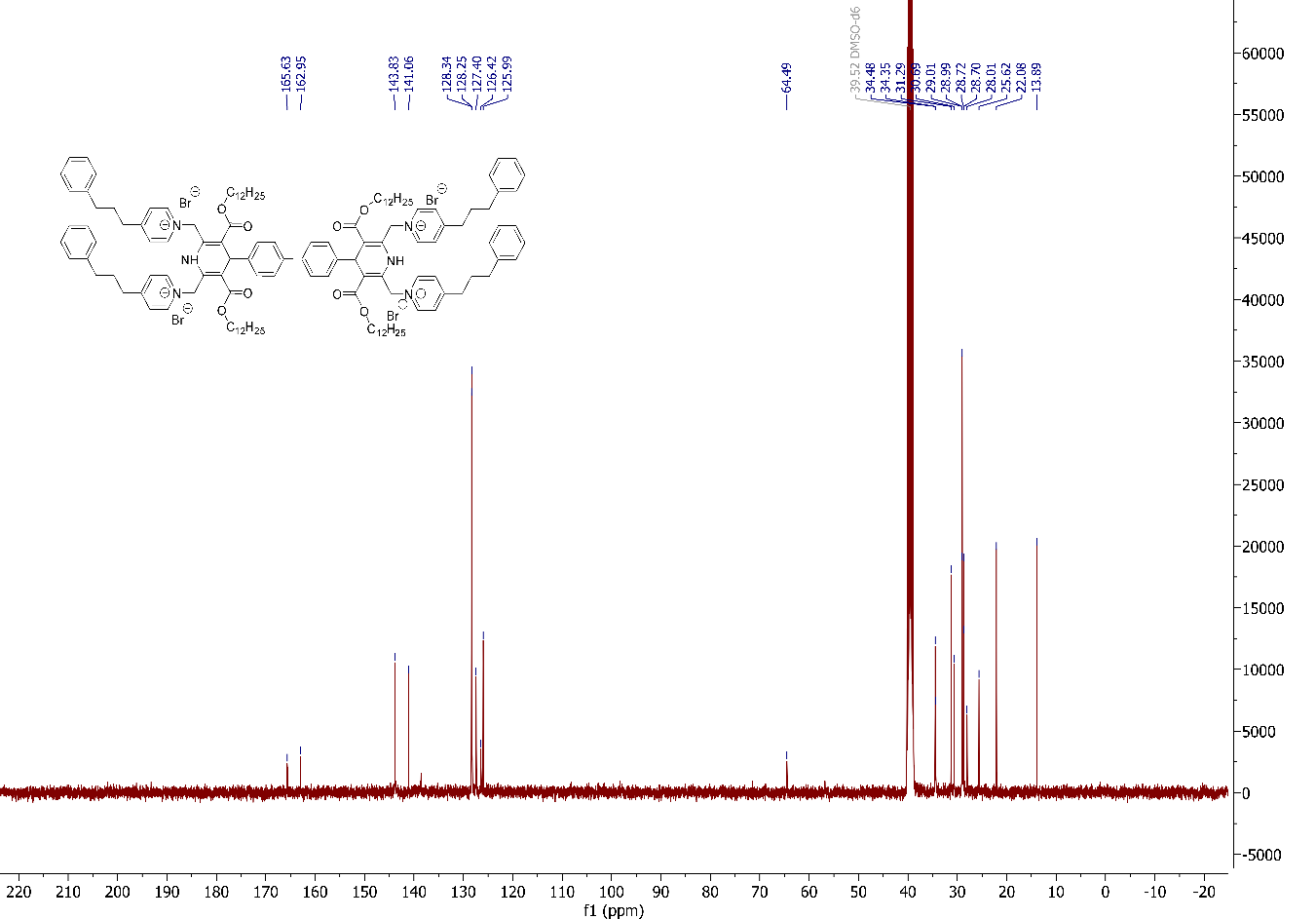


**Figure S40.** ^13^C NMR spectrum of didodecyl 4-[4-[4-[3,5-*bis*(dodecoxycarbonyl)-2,6-*bis*[[4-(3-phenylpropyl)pyridin-1-ium-1-yl]methyl]-1,4-dihydropyridin-4-yl]phenyl]phenyl]-2,6-*bis*[[4-(3-phenyl-propyl)pyridin-1-ium-1-yl]methyl]-1,4-dihydropyridine-3,5-dicarboxylate tetrabromide (**27b**).


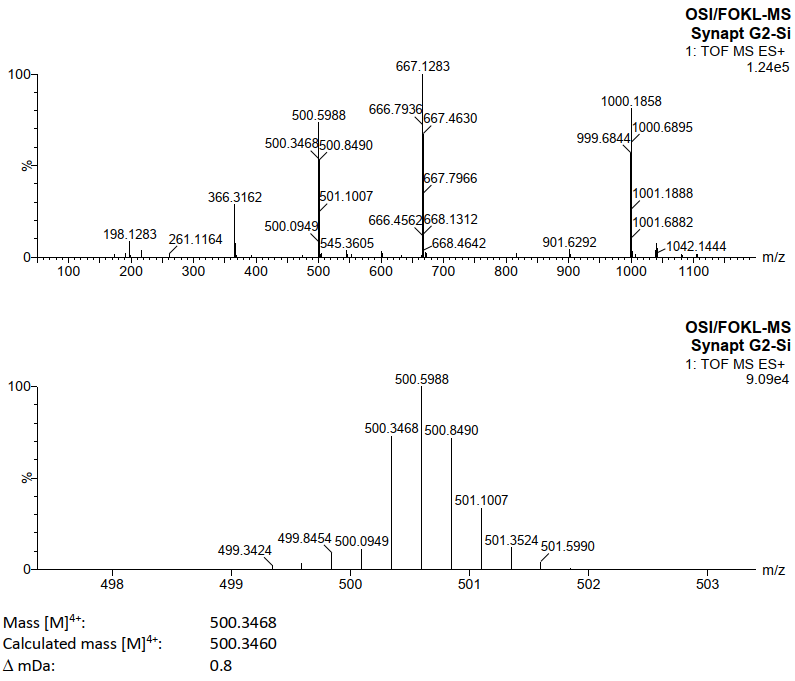


**Figure S41.** HRMS data of didodecyl 4-[4-[4-[3,5-*bis*(dodecoxycarbonyl)-2,6-*bis*[[4-(3-phenylpropyl)pyridin-1-ium-1-yl]methyl]-1,4-dihydropyridin-4-yl]phenyl]phenyl]-2,6-*bis*[[4-(3-phenyl-propyl)pyridin-1-ium-1-yl]methyl]-1,4-dihydropyridine-3,5-dicarboxylate tetrabromide (**27b**).

**Table S1**. Dynamic light scattering measurement results of non-extruded samples of compounds **22a**,**b**-**27a**,**b**.

| **Entry** | **Comp.** | **Freshly prepared** | | **3 days** | | | **7 days** | | **14 days** | |
| --- | --- | --- | --- | --- | --- | --- | --- | --- | --- | --- |
|  |  | **Z_av_ D_h_, nm** | **PDI** | **Z_av_ D_h_, nm** | **PDI** | **Z-Potential, mV** | **Z_av_ D_h_, nm** | **PDI** | **Z_av_ D_h_, nm** | **PDI** |
| 1 | **22a** | 61 ± 1 | 0.205 ± 0.014 | 100 ± 1 | 0.368 ± 0.013 | 24.6 ± 0.5 | 70 ± 1 | 0.263 ± 0.014 | 71 ± 1 | 0.269 ± 0.029 |
| 2 | **22b** | 1142 ± 30 | 0.887 ± 0.037 | 412 ± 11 | 0.403 ± 0.086 | 31.0 ± 1.4 | 358 ± 40 | 0.392 ± 0.022 | 351 ± 21 | 0.260 ± 0.090 |
| 3 | **23a** | 156 ± 11 | 0.783 ± 0.093 | 301 ± 6 | 0.560 ± 0.009 | 24.8 ± 0.5 | 471 ± 25 | 0.829 ± 0.136 | 328 ± 1 | 0.232 ± 0.017 |
| 4 | **23b** | 1039 ± 102 | 0.900 ± 0.089 | 635 ± 360 | 0.599 ± 0.244 | 18.0 ± 1.9 | 221 ± 31 | 0.506 ± 0.192 | 465 ± 185 | 0.498 ± 0.149 |
| 5 | **24a** | 3111 ± 675 | 1 ± 0 | 589 ± 127 | 0.589 ± 0.099 | 10.9 ± 0.4 | 1875 ± 716 | 0.765 ± 0.284 | 423 ± 84 | 0.474 ± 0.209 |
| 6 | **25a** | 364 ± 35 | 0.521 ± 0.085 | 376 ± 2 | 0.141 ± 0.028 | 9.7 ± 0.8 | 443 ± 19 | 0.139 ± 0.045 | 395 ± 7 | 0.028 ± 0.029 |
| 7 | **25b** | 639 ± 90 | 0.516 ± 0.091 | 431 ± 94 | 0.481 ± 0.019 | 24.9 ± 1.1 | 599 ± 42 | 0.724 ± 0.112 | 307 ± 6 | 0.298 ± 0.123 |
| 8 | **26a** | 1168 ± 342 | 0.850 ± 0.171 | 585 ± 118 | 0.580 ± 0.148 | 23.4 ± 1.1 | 656 ± 62 | 0.549 ± 0.121 | 337 ± 50 | 0.773 ± 0.197 |
| 9 | **26b** | 622 ± 12 | 0.500 ± 0.012 | 341 ± 7 | 0.340 ± 0.049 | 11.9 ± 0.9 | 365 ± 6 | 0.306 ± 0.023 | 349 ± 3 | 0.215 ± 0.162 |
| 10 | **27a** | 536 ± 138 | 0.544 ± 0.108 | 376 ± 14 | 0.549 ± 0.121 | 20.9 ± 1.0 | 264 ± 11 | 0.365 ± 0.030 | 285 ± 7 | 0.167 ± 0.085 |
| 11 | **27b** | 1351 ± 455 | 0.880 ± 0.207 | 1098 ± 112 | 0.533 ± 0.110 | 23.8 ± 0.2 | 2102 ± 79 | 0.573 ± 0.074 | - | - |


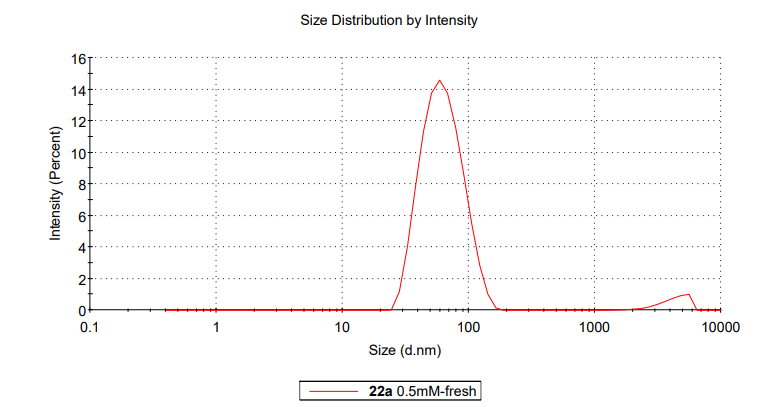


**Figure S42.** Dynamic light scattering measurement results of a freshly prepared sample of **22a**.


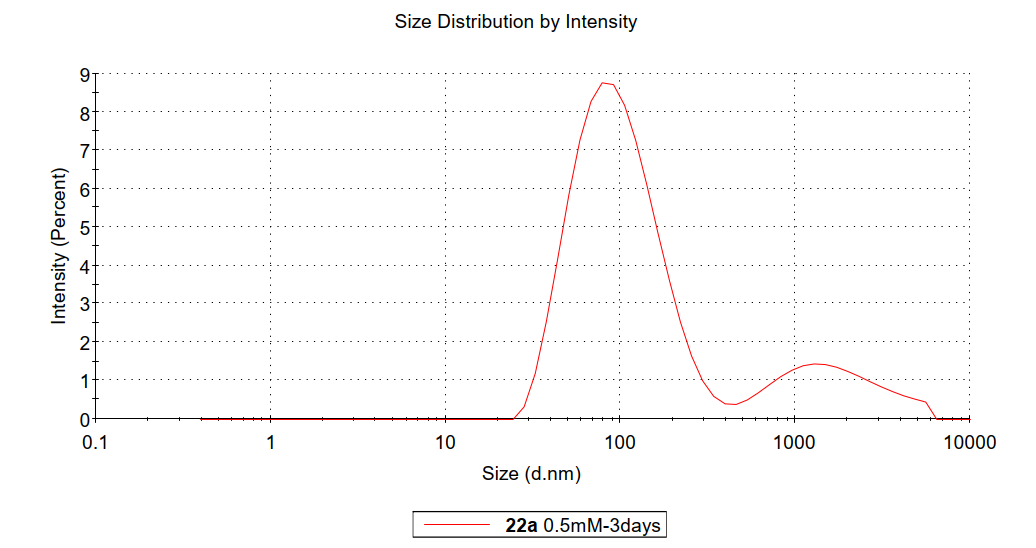


**Figure S43.** Dynamic light scattering measurement results of compound **22a** sample after 3 days of storage.


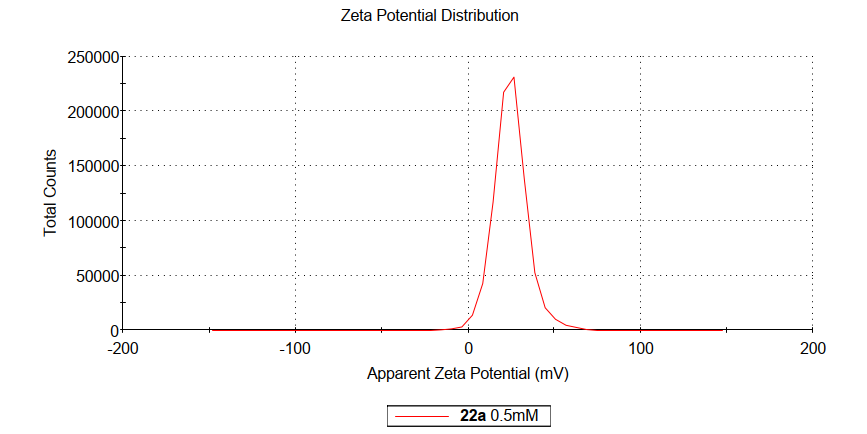


**Figure S44.** Zeta potential measurement results of compound **22a**.


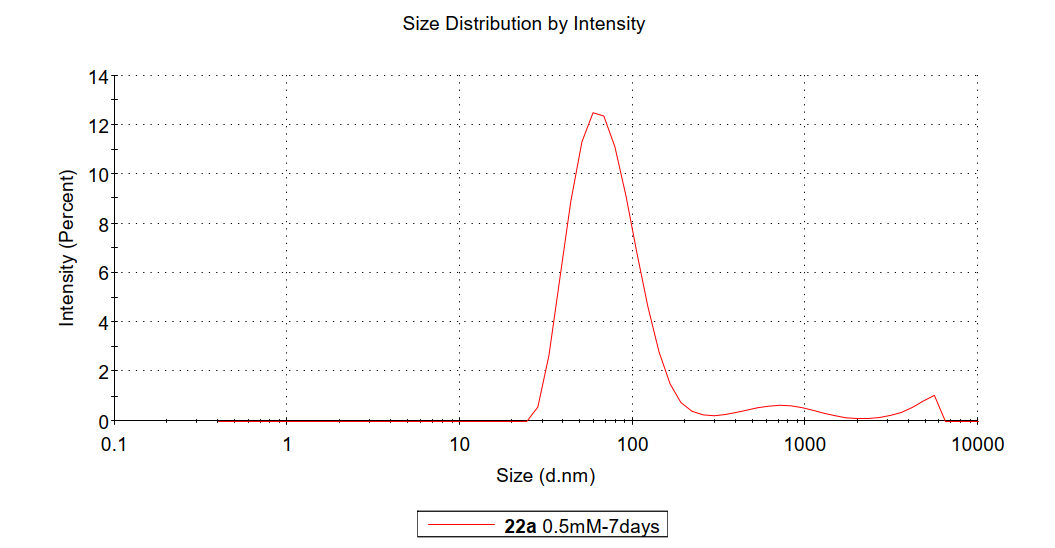


**Figure S45.** Dynamic light scattering measurement results of compound **22a** sample after 7 days of storage.


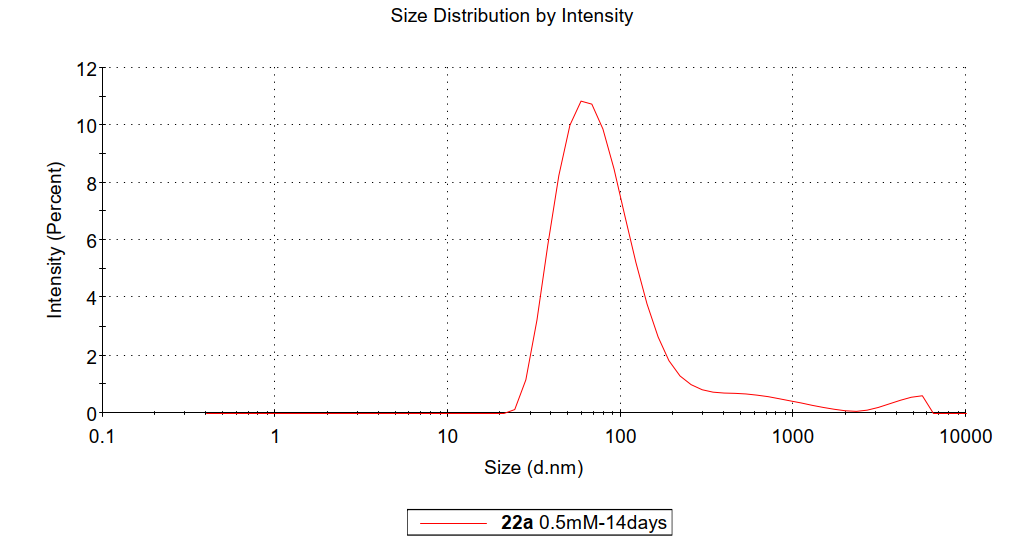


**Figure S46.** Dynamic light scattering measurement results of compound **22a** sample after 14 days of storage.
